# reString: an open-source Python software to perform automatic functional enrichment retrieval, results aggregation and data visualization

**DOI:** 10.1038/s41598-021-02528-0

**Published:** 2021-12-06

**Authors:** Stefano Manzini, Marco Busnelli, Alice Colombo, Elsa Franchi, Pasquale Grossano, Giulia Chiesa

**Affiliations:** 1 Department of Pharmacological and Biomolecular Sciences, Università Degli Studi Di Milano, Milan, Italy; 2 Fondazione IRCCS Ca’ Granda Ospedale Maggiore Policlinico, Milan, Italy

**Keywords:** Data mining, Data processing, Gene ontology, Software

## Abstract

Functional enrichment analysis is an analytical method to extract biological insights from gene expression data, popularized by the ever-growing application of high-throughput techniques. Typically, expression profiles are generated for hundreds to thousands of genes/proteins from samples belonging to two experimental groups, and after ad-hoc statistical tests, researchers are left with lists of statistically significant entities, possibly lacking any unifying biological theme. Functional enrichment tackles the problem of putting overall gene expression changes into a broader biological context, based on pre-existing knowledge bases of reference: database collections of known expression regulation, relationships and molecular interactions. STRING is among the most popular tools, providing both protein–protein interaction networks and functional enrichment analysis for any given set of identifiers. For complex experimental designs, manually retrieving, interpreting, analyzing and abridging functional enrichment results is a daunting task, usually performed by hand by the average wet-biology researcher. We have developed reString, a cross-platform software that seamlessly retrieves from STRING functional enrichments from multiple user-supplied gene sets, with just a few clicks, without any need for specific bioinformatics skills. Further, it aggregates all findings into human-readable table summaries, with built-in features to easily produce user-customizable publication-grade clustermaps and bubble plots. Herein, we outline a complete reString protocol, showcasing its features on a real use-case.

## Introduction

In the recent years, -omics techniques—which allow the analysis of samples “as a whole”, including genomics and epigenomics, transcriptomics, proteomics, metabolomics and lipidomics—have rapidly transitioned from niche commodities to widespread tools^1^. Array- or next generation sequencing-based methods allow for the simultaneous analysis of the expression profile of tens of thousands of genes^2^, and mass-spec based methods can quantitatively resolve hundreds of different proteins within a sample^3,4^.

In a typical experimental workflow, the abundance of mRNA transcripts or proteins are obtained for hundreds to thousands of analytes at the same time, and their increased or decreased abundance with respect to a reference experimental group is evaluated with *ad-hoc* statistical tools. The analytes that survive the false discovery rate (FDR) threshold face researchers with the challenge of extracting biologically relevant information from them.

Usually, genes with the most extreme fold change between experimental groups (likely mirrored by lower FDR) are investigated to a deeper level in search of a biological mechanism, yet often the vast majority of leftover genes are so numerous, showing only moderated fold changes, that the challenge of extracting more information requires more advanced tools. Noteworthily, a meager ~ 20% difference in the expression levels of genes all members of the same pathway may hold more clues than a 20-fold change in the expression of a single gene^5^.

To put all the changes in the abundance of analytes into a wider biological context, these are arranged into sets on the basis of pre-existing knowledge about their biological function, including—but not limited to—functional properties, known interactions, shared regulation, phenotype associations and molecular products. An incredible variety of different methods has been generated to analyze these sets as a whole^6^. Further, there is a broad choice of knowledge bases, including the famed Kyoto Encyclopedia of Genes and Genomes (KEGG)^7–10^, Gene Ontology (GO) terms^11^ and Reactome pathways^12^. By tapping these knowledge bases, functional enrichment analysis can really extract biological insights from -omics data, in their literal meaning of “as a whole”.

Many websites offer friendly frontends to provide users with the possibility of upload custom lists of identifiers and retrieve functional enrichment info, such as DAVID^13,14^ (https://david.ncifcrf.gov/), Enrichr^15,16^ (https://maayanlab.cloud/Enrichr/), WebGIVI^17^ (http://raven.anr.udel.edu/webgivi/), g:Profiler^18^ (https://biit.cs.ut.ee/gprofiler/gost) and STRING^19^ (https://string-db.org/). While this approach might be handy for a few searches, this manual approach rapidly becomes extensively tiring and time-consuming. While it is possible to automatize the process by interacting with most of these services via custom APIs (application programming interface), this approach is viable only to those researchers with specific bioinformatics skills.

We have developed reString, a software that leverages STRING APIs to automatically perform functional enrichment analysis on multiple user-provided gene/protein lists, rendering the handling of even the most complicate experimental layout a lightweight task. One key feature of reString is the ability of aggregating the results from functional enrichment of different experimental comparisons, and produce human-readable summaries that can be furthermore tailored to the user’s needs, visualized and saved as publication-grade clustermaps and bubble plots. reString is cross-platform and all its features can be easily accessed via a graphical user interface (GUI).

Herein, we outline reString features by outlining a detailed step-by-step protocol that can be easily practiced with included sample files. Further, we process differentially expressed gene lists from an own, real-world dataset to further showcase the reString application.

## Materials and methods

### Software

Data was processed with SciPy (version 1.3.1)^20^, Numpy (version 1.15.4)^21^, and Pandas (version 0.23.4)^22^. Tabular data images were made with LibreOffice’s Calc (https://www.libreoffice.org/). Data visualization was performed with matplotlib (version 3.0.2)^23^ and seaborn (version 0.9.0)^24^ libraries for the Python programming language. reString can be automatically installed via pip, the Python package installer (as detailed in “Procedure”), downloaded from GitHub (https://github.com/Stemanz/restring) or from the Python package Index, PyPI (https://pypi.org/project/restring/).

### Software setup

reString is a Python program that needs a working Python 3.6 + environment. In its GUI form it leverages tkinter^25^, the Python binding to the Tk GUI toolkit, which is included in most distributions. reString will work out of the box (when installed via pip, see below) within a standard Python installation that can be obtained at https://www.python.org/ for Windows, macOS, GNU/linux distributions and other platforms.

### Statistical and analyses

Statistical analyses are detailed for each individual analysis in the appropriate figure or table caption, and were performed with GraphPad Prism software version 9.1.1 (223).

### Procedure

The following protocol illustrates how to prepare an environment to use reString, as well as how to analyze sample RNAseq results. An active internet connection is required to download software and data. Further detailed and up-to-date information on additional features of reString can be found at the project’s repository (https://github.com/Stemanz/restring).

### Procedure–installation

The following describes a general protocol to set up Python 3.x in the system as well as to install reString.

For a more specific protocol, please refer to the online documentation or Supplementary Materials and Methods at “Installation in depth” to watch YouTube videos with step-by-step instructions tailored for Windows 10, Ubuntu GNU/linux, Raspberry Pi OS and Mac OS fresh installs, or search YouTube for “How to install reString in” followed by your OS name. A following section, “Installation troubleshooting”, will cover and provide solutions to most common hiccups that might happen during the installation.

Some commands need to be executed from the UNIX shell (the Terminal app) and are prefixed by the “$” symbol, which shall not be inputted with the commands. The same commands can be inputted in the Windows Command Prompt (Users would see a “>” symbol at the end instead of a “$” symbol).

1. *Download and install Python.* Head over to https://www.python.org/downloads/ and get the latest Python release for your operating system. Windows users can also install Python through the Microsoft store. Please note that Python might already be installed in your system; check whether this is the case and also whether its version is 3.8 or greater (open a terminal—see below—and type ‘python --version’; inspect the output. Windows users should alternatively search for Python in the Start Menu, or type ‘py’ in a terminal). Install the software by following on screen instructions.

An alternative easy way to obtain an up-to-date Python distribution, packed with pre-installed scientific libraries, is installing the free and open source Individual Edition of Anaconda, available for Windows, Linux and Mac (https://www.anaconda.com/), as outlined here^26^.

1. *Optional for GNU/linux*: While all libraries needed by reString are included by default (or being installed) in MacOS and Windows, GNU/Linux distros vary greatly in this respect.We can assume that the average GNU/Linux user will be able to address any installation/dependency issue that may incur. A few tips: many distros may bundle both Python 2.x and 3.x: in this case, python3 and pip3 should be used instead of python and pip commands. Additionally, not all Debian-based distro include tkinter, a library which reString rely upon. This can be fixed with this terminal command (please otherwise refer to updated or specific distros documentation for any issue):
  $ sudo apt-get install python3-tk
2. *Open a terminal*. On macOS, this is done by running the Terminal app from the Utilities (to access Utilities, from Finder ⌘ + ⇧ + U, or Go > Utilities). On Windows, bring up the Start menu and type ‘cmd’ in the search field, then run it.
3. *Install reString*. reString and its dependencies can be automatically installed from the command line. Type:
  $ pip install restring
4. *Optional troubleshooting for Windows*: it is possible that after the installation the system does not know where to find pip, the Python package manager. Should this occur, locate the folder containing it by typing in a terminal:
  > cd\
    > dir pip.exe /a /s
  The folder containing it (for example: C:\Users\username\AppData\Local\Programs\Python39\Scripts) needs to be added to the environment PATH variable. Start typing “environment variables” in the Windows search box, and click on “Edit the system environment variables”. Open a dialog by clicking on “Environment variables”, then double-click on PATH. Add the folder to the list, OK and exit. Close and reopen the terminal.
5. *Run reString*. The installation takes care of creating a script that automatically runs the graphical user interface (GUI), that can be invoked directly from the terminal:
  $ restring-gui
  reString should launch and the User should see the program’s main window (Supplementary Fig. S1). Steps 1 and 3 will not be needed anymore to run reString.
  If the program fails to start (this might happen on some GNU/linux systems and Windows), alternatively type:
  $ python -c "import restring; restring.restring_gui()"
  Please note that on some Windows setups the antivirus might scan restring-gui.exe for threats. This is normal and should not take longer than a few seconds. Also on Windows, if the system fonts are scaled to 125% or above, reString fonts might be displayed too large. Reduce system fonts scaling to 100% to solve the issue.
  *Update reString*. To periodically ensure that reString is up to date, type in the terminal:
  $ pip install restring --upgrade

### Procedure–analysis

The protocol is illustrated through an example experiment which makes use of sample files, that Users can analyze to familiarize themselves with the file format accepted by reString. Ideally, each file should have a name that serves as the label for the experimental condition. The file structure is detailed in Supplementary Fig. S2.

1. *Download sample files*: in the program’s main app, choose “File > Download sample data”. Alternatively, download them from https://github.com/Stemanz/restring/raw/main/data/restring_sample_tables.zip.
2. *Prep sample files:* After downloading sample data (the file is called restring_sample_tables.zip and is found in the default browser’s download folder, usually Downloads), unzip the folder and copy it over to any desired location. In our example, we will be using the home directory (on the Mac, Finder > Go > Home, or ⌘ + ⇧ + H).
3. *Create the output folder*. Create a folder of choice to store the results. In this example, we will create the folder output within the sample data folder.
4. *Choose the input files*. Tell reString what input files to process, choose “File > Open...” or click “Open files..”. The file choosing dialog will open (Supplementary Fig. S3). Select all of them and click “Open”. For each file successfully opened, reString prints a message on the textual output frame (Supplementary Fig. S4). The frame is scrollable so that Users can always inspect each step of the analysis. When working with your files, put all input files you want to process together, in one or more analyses, in the same folder. Input files can be individually selected from any one folder, but each time input files are added, the input files list is reset.
5. *Choose the output folder.* Choose a previously created folder to store the analysis results. Choose “File > Set output folder” or click “Set folder” to open the dialog (Supplementary Fig. S5).
6. *Run the analysis with defaults settings*. Choose “Analysis > New analysis” or click the “New analysis” button to start retrieving and aggregating results automatically. Please note that the computer must be connected to the internet. Refer to “Procedure–analysis parameters” to learn about all settings.

**Figure 1:**
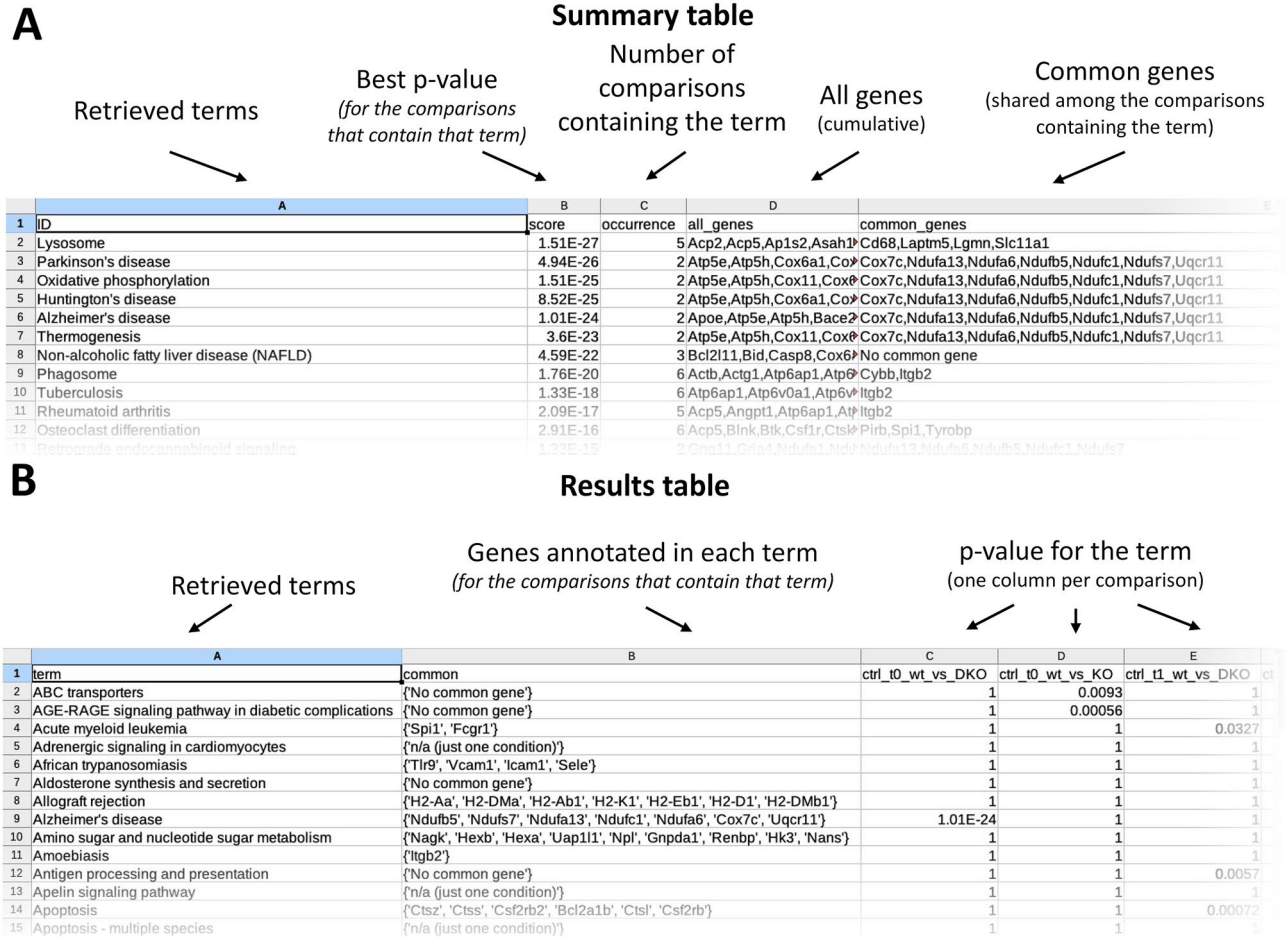
Structure of the summaries generated by reString. When running an analysis, reString will look for identifiers in the files specified, interrogate STRING to get functional enrichment data back (these tables, analogous to the ones that would be manually retrieved, will be saved into subfolders of the output folder), then write aggregated results and summaries. These are found in the specified output directory, and take the form of “results”- or “summary”-type tables, in .tsv (tab separated values) format, that can be opened out-of-the-box by Excel or Calc.

reString will automatically retrieve from String functional enrichment information for statistically significant terms from KEGG Pathways, Gene Ontology (Biological Processes, Molecular Function and Cellular Component), and Reactome Pathways knowledge bases. These will be stored in a subfolder of the output folder with the same name as the input file and are equivalent to those that Users can manually download from STRING (Supplementary Fig. S6). Depending which genes were selected in the analysis, file names are prepended with “UP_”, “DOWN_” or “ALL_” (upregulated, downregulated and all genes simultaneously, see “Procedure–analysis parameters”). reString details all steps it takes to retrieve functional enrichment analysis information automatically from STRING (Supplementary Fig. S7). reString will then aggregate retrieved results (Supplementary Fig. S8) and, for each functional enrichment searched (KEGG, Function, Component, Process and RCTM), produce two tables that contain the abridged version of the whole analysis: results and summary (Fig. 1).

### Procedure–analysis parameters

The retrieval of functional enrichment information and the following aggregation is performed by reString with default settings, that can be adjusted.

#### Species

reString defaults to *Mus musculus*, but a different species can be selected: “Analysis > Set species” will open the species selection dialog (Supplementary Fig. S9). For species that are not listed, a taxonomy identifier can be manually set.

#### Upregulated and/or downregulated genes

reString knows from the input files whether genes/proteins are up- or downregulated in any given comparison between two conditions (Supplementary Figs. S2 and S10). This allows four types of different analyses to be selected via “Analysis > DE genes settings”: (i) “Upregulated genes only”: Functional enrichment info is searched for upregulated genes only; (ii) “Downregulated genes only”: Functional enrichment info is searched for downregulated genes only; (iii) “Upregulated and Downregulated, separately”: This is the default option. For every comparison, both upregulated and downregulated genes are considered, but separately. This means that functional enrichment info is retrieved for upregulated and downregulated genes separately, but the terms are aggregated from both. If a term shows up in both UP and DOWN gene lists, then the lowest P-value is recorded; (iv) “All genes together”: Functional enrichment info is searched for all genes together, and the resulting aggregation will reflect the functional enrichment analysis retrieved with all genes combined.

#### STRING API version

reString defaults to using the latest STRING version. To maintain compatibility with previous versions, it is possible to choose a specific version via “Analysis > Choose STRING version”.

#### Setting a statistical background

The enrichment analysis on the input is performed by STRING, which will list any term observed more frequently than expected against a background^19^. This defaults to the entire genome of the selected organism, but it can otherwise be set to a user-specified list of identifiers. A headerless, one-column table or plain textfile with one identifier per line can be set via “Analysis > Custom background”. To clear the custom background and revert to the default setting, “Analysis > Clear custom background”.

### Procedure–data visualization

reString integrates a flexible data visualization tool that aids Researchers to visualize the aggregated results and produce publication-quality heatmaps and clustermaps with a few clicks. The window can be opened via “Analysis > Draw clustermap” (Supplementary Fig. S11), and it is intended to work with reString results-type tables (Fig. 1). The tool produces customizable heatmaps or clustermaps for each enriched term across selected experimental conditions.

Another built-in tool has been designed to work with reString summary-type tables (Fig. 1), accessed through “Analysis > Draw bubble plot” (Supplementary Fig. S12). This produces a bubble plot, where for all enriched terms three stats are graphically displayed: the number of all genes annotated for that term in all experimental conditions (*x* axis), the number of common genes across all experimental conditions (bubble size) and the lower FDR score of the term in all experimental condition (color).

Detailed information on each tool and option can be found in the Supplementary Materials and Methods or in the online documentation (https://github.com/Stemanz/restring/blob/main/README.md).

### Animals and experimental procedures

Procedures involving animals and their care were conducted in accordance with institutional guidelines, in compliance with national (D.L. No. 26, March 4, 2014, G.U. No. 61 March 14, 2014), international (EEC Council Directive 2010/63, September 22, 2010: Guide for the Care and Use of Laboratory Animals, United States National Research Council, 2011) laws and policies and the ARRIVE guidelines^27^. The experimental protocol was approved by the Italian Ministry of Health (Protocollo 2012/4).

Apoe knockout (EKO) mice (https://www.jax.org/strain/002052) in the C57BL/6J background were purchased from Charles River Laboratories (Calco, Italy); double Apoe and Apoa1 knockouts (DKO) were generated as previously described^28,29^.

Eight weeks old male mice were randomly divided, genotype-wise, into 8 groups and fed either a normal laboratory diet (NLD, 4RF21, Mucedola, Italy) or a Western-type diet (WD, TD.88137, Envigo, Italy) for 6 or 22 weeks, and sacrificed as described^30^. Briefly, mice were euthanized by exsanguination under general anesthesia with 2% isoflurane (Merial Animal Health, Woking, UK), where blood was removed by perfusion with 1 × PBS. Aortas were then snap-frozen in liquid nitrogen for RNA-seq analyses (n = 3) or longitudinally opened, pinned flat on a black wax surface in ice-cold PBS and photographed unstained for en face analysis (n = 6–7)^30–36^.

### RNA extraction

Total RNA was isolated from mouse aorta and extracted as previously described^36^. RNA was quantified and purity was checked, and 1 μg RNA was retrotranscribed to cDNA, as described^37^. Possible gDNA contamination was ruled out by running a PCR on 20 ng of cDNA/RNA with a primer pair producing two amplicons of different size on cDNA (193 bp) and gDNA (677 bp), see Supplementary Table S1 and Supplementary Fig. S13.

### Quantitative PCR

Twenty ng of cDNA were used as template for each qPCR reaction, performed on a CFX Connect thermal cycler with iTAQ Universal Sybr Green Supermix (Bio-Rad, Segrate, Italy). Conditions and primers are detailed in Supplementary Table S1. A final melting curve analysis was always performed. Fold changes relative to the control group were calculated with the ΔΔCt method^38^. The gene cyclophilin A (Ppia) was used as reference gene^39^.

### RNA-seq analyses

The quality of the mRNA was tested using the Agilent 2100 Bioanalyzer (Agilent Technologies, Santa Clara, CA, USA) prior to RNAseq; samples with RIN < 7.0 were discarded. RNA samples were processed using the RNA-Seq Sample Prep kit from Illumina (Illumina, Inc., CA, USA). Clusters of tagged libraries (8–9 per single Illumina flowcell, created using the Illumina Cluster Station) were sequenced on a Genome Analyzer IIx (Illumina, Inc., CA, USA) to produce 50 nt-long, unpaired reads. Reads were mapped on the UCSC genome assembly mm10 (reference strain C57BL6/J) using the classic tuxedo suite bowtie and tophat programs^40^. Estimation of gene expression levels was performed using cufflinks^40^. Genes with an adjusted *P* value lower than 0.05 were considered differentially expressed (DE). All data and materials have been made publicly available at NCBI GEO. Data sets can be accessed at (https://www.ncbi.nlm.nih.gov/geo/query/acc.cgi?acc=GSE173974)﻿.

### Data processing and visualization

Gene ontology analyses were performed with reString by querying STRING^41^ as thoroughly described in this manuscript; and terms with adjusted P-values lower than 0.05 were considered significant. Principal Component Analysis was performed with Scikit-learn^42^. Data visualization was performed with reString, as well as SciPy^43^, matplotlib^23^ and seaborn^24^ libraries for the Python programming language.

## Results and discussion

We have developed reString as a tool to help Researchers without prior bioinformatics knowledge tackle the issue of retrieving, analyzing, and summarizing functional enrichment results from complex experimental layouts.

**Figure 2:**
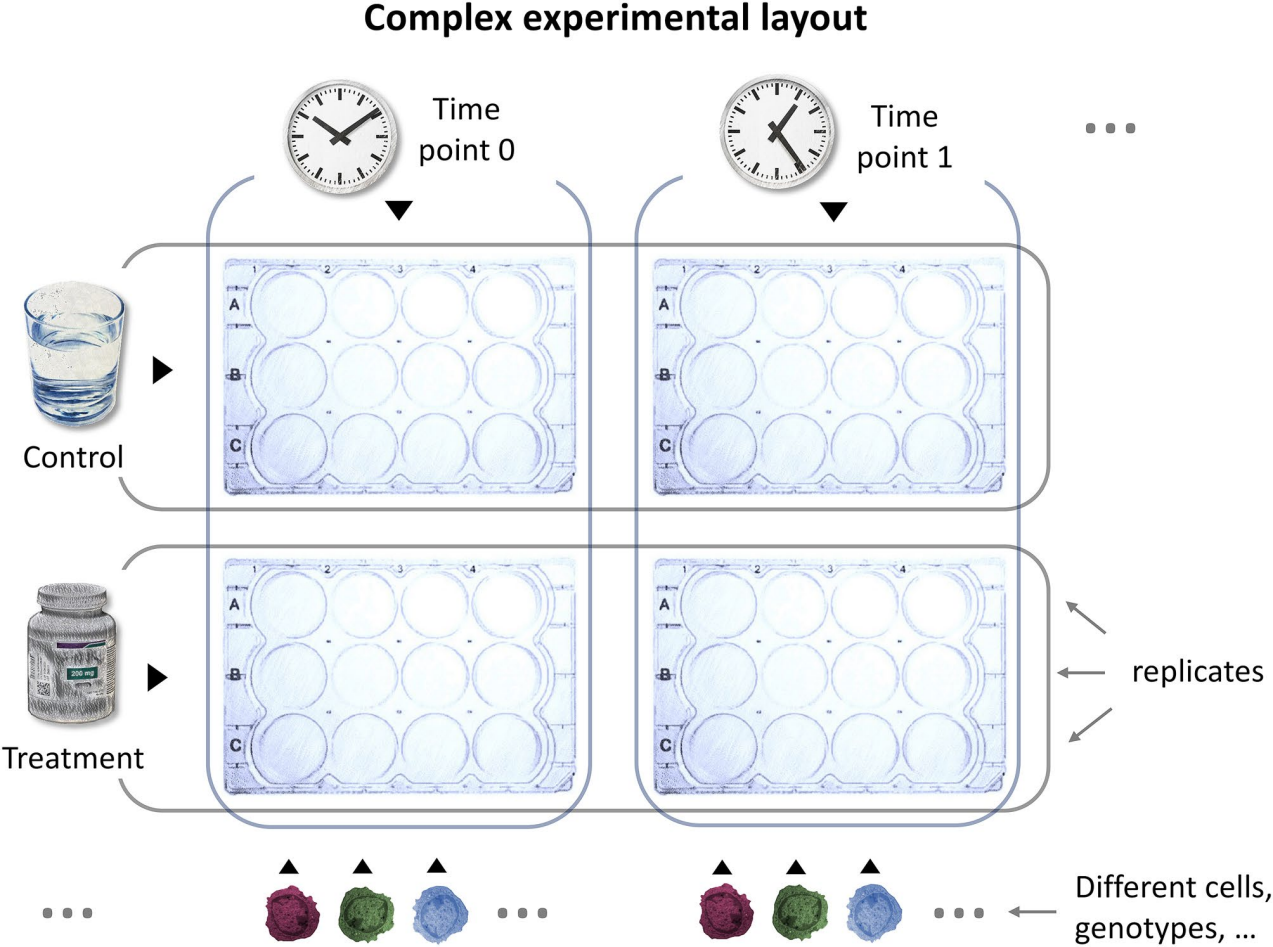
Hypothetical scheme of an experimental setup. Modern high-throughput -omic approaches generate huge lists of differentially expressed (DE) genes/proteins, which can in turn be used for functional enrichment studies. Manually reviewing a large number of such analyses is time consuming, especially for experimental designs with more than a few groups. A prototypical example is shown in the picture. This example setup has two treatments, given at two time points to three different sample types (represented with different colors). While this represents a fairly common experimental design, the inspection of functional enrichment results for such all possible combinations would require substantial effort. reString makes it easy to automatically deal with this situation.

**Figure 3:**
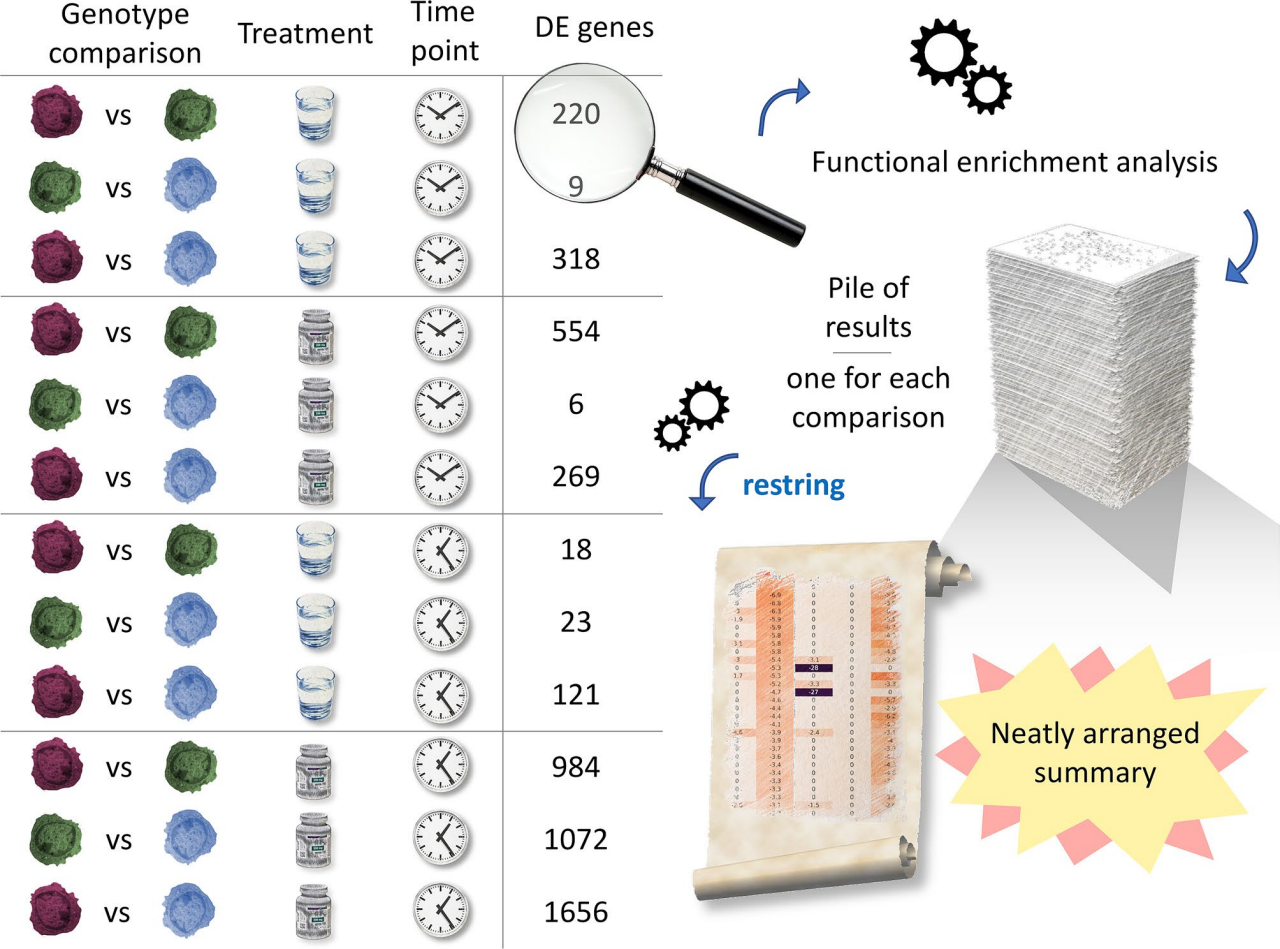
Comparisons generated from the example experimental setup. The example experimental setup has two treatments, given at two time points to three different sample types (represented with different colors). After quantifying gene expression by RNAseq, the experimental workflow (either managed in-house or sent to the Researcher by a company) generates DE genes lists for every comparison. As in many experimental pipelines, each list of DE genes is investigated with functional enrichment tools, such as STRING. Every comparison generates one or more tables (depending on the criteria the genes of interested are selected with), and reString makes it easy to generate summary reports from all of them, automatically.

A typical, moderately sized experimental layout is shown in Fig. 2. Therein, three different sample types (in our example, identified with different colors: red, blue and green) are subjected to two different treatments, whose impact is assayed at two different time points by RNAseq. This translates to 12 different comparisons, if the focus of the experiment is prioritizing the differences among the genotypes, as schematized in Fig. 3. The task of manually retrieving, inspecting and analyzing functional enrichment results from all these analyses looks daunting, yet that is how it is managed in a sizeable number of research groups.

reString was designed to work with input tables that are only required to contain the desired genes and the fold change information of each gene (Supplementary Fig. S2). To obtain a list of genes or proteins to work with, a conventional approach is filtering the full list of entries, that can comprise hundreds to thousands of identifiers, by means of P-value, false discovery rate or desired fold change threshold. RNAseq or proteomics services provide tabular data that contain this information which can be easily handled by simple spreadsheets, such as Microsoft Office’s Excel or LibreOffice’s Calc.

With reString, retrieving functional enrichment data and producing summaries from the 12 sample data tables takes no longer than ~ 5 min. With a few further clicks, results can be visualized and saved as clustermaps and bubble plots.

**Figure 4:**
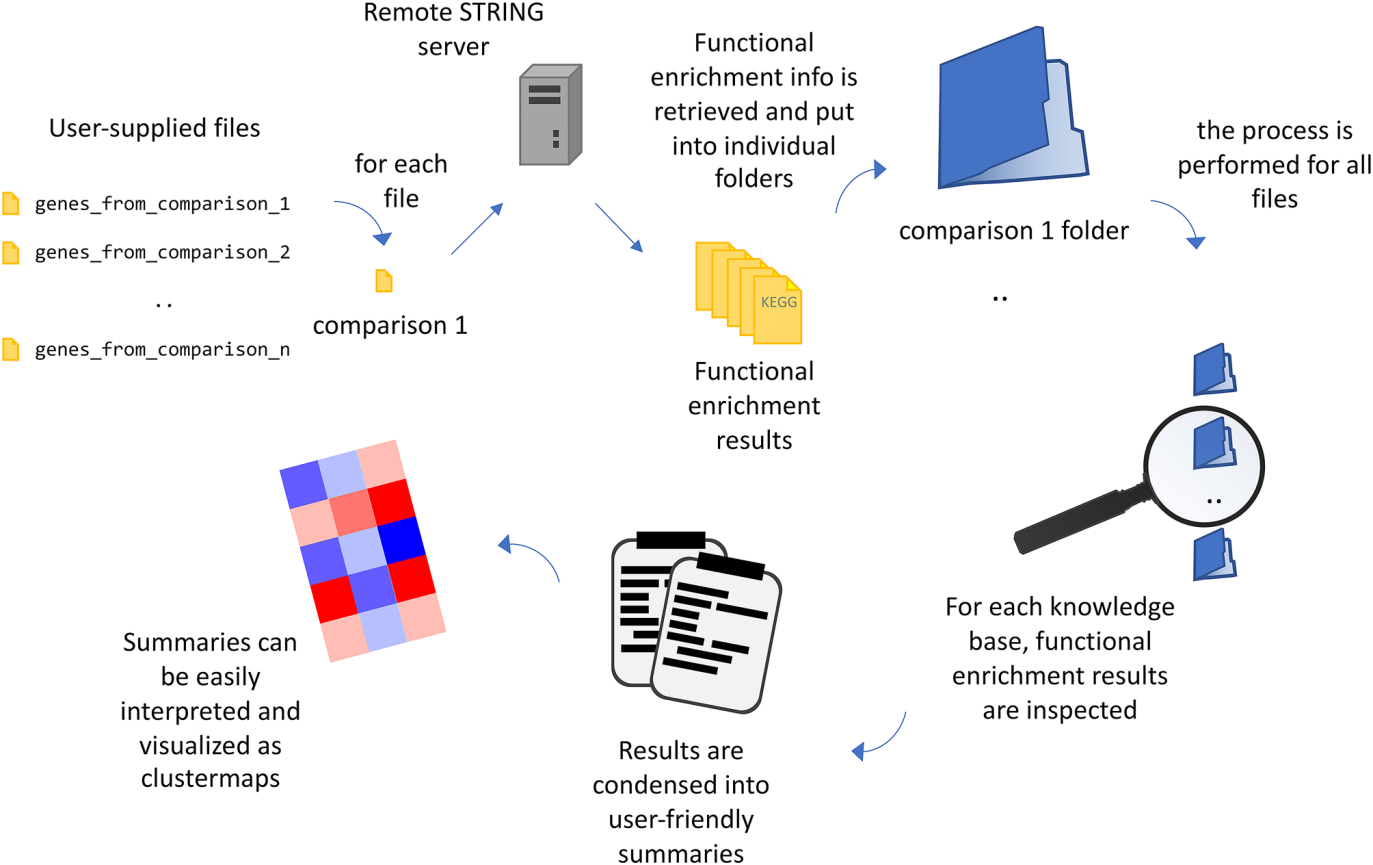
Schematics of reString workflow. reString works with user-supplied files that contain gene or protein identifiers, along with information of their expression in two experimental conditions in the form of fold change. For each input file, reString retrieves functional enrichment from the remote STRING server, then creates a folder where it stores the results (for KEGG Pathways, Reactome Pathways, and gene Ontology Biological Processes, Molecular Function and Cellular Component). Depending on the analysis settings, results can be retrieved for upregulated or downregulated genes only, for both up- and downregulated genes taken separately, or from all genes simultaneously. Then, results are condensed and abridged into user-friendly summaries.

reString leverages STRING APIs to automatically retrieve functional enrichment information from KEGG^7^, GO^11^ (Biological Processes, Molecular Function, Cellular Component), and Reactome^12^ pathways knowledge bases. For each User-supplied file, reString (depending on the analysis settings) either splits the gene list into up- or downregulated genes, or takes all genes, and queries the remote server retrieving functional annotation information, if the gene lists contain sufficient elements to produce statistically significant results. The five knowledge bases considered produce tab-delimited text files that are each saved in a folder bearing the same name of the input file from which it was generated, and are available to the Researcher for further inspection. These files are analogous to the ones that would be downloaded by hand from the STRING website (Supplementary Fig. S6). File names are prepended by reString with either “UP_, “DOWN_” or “ALL_” as a way to identify whether they were produced with upregulated, downregulated or all genes of each input file, respectively. After fetching functional enrichment data, for each knowledge base (internally referred to as *kind* within the application code and documentation), reString produces two types of abridged summaries by aggregating information from files of all comparisons (Fig. 1). The whole procedure is summarized in Fig. 4.

Summary-type tables contain, for each term (the nature of the term depends on the type of knowledge base queried, for instance KEGG pathways or Reactome pathways), the lower P-value score ever observed across all comparisons, the number of comparisons where the term has been found enriched in a statistically significant way (indicated with “occurrence” in the table), a list of unique identifiers gathered from all comparisons (“all_genes” column), and a list of identifiers that is shared between all comparisons where the term is statistically significant (“common_genes” column). In the case there are no shared genes, even though the term is found across multiple comparisons, reString marks the condition as “No common gene”. Conversely, if the term shows up in only one comparison, reString marks the condition as “n/a: just one condition”, and the gene identifiers of that one condition can be found under “all_genes” (Fig. 1A). This summary is useful to rapidly identify the strongest (by statistical significance) terms with respect to all comparisons simultaneously—or a subset thereof, as well as to readily identify the underlying genes.

Results-type tables contain, for each term, the genes that were found common among all comparisons, as well as the corresponding FDR-value of that term in every comparison (one column per comparison, Fig. 1B). In the case a comparison failed to enrich in a statistically significant way for the term being considered, a *P* value of 1 is assigned by default. In case the aggregation is set to consider upregulated and downregulated genes separately (see “Procedure–analysis parameters” in Materials and methods), in the unlikely case the term is enriched in both, the lowest P-value is taken into account. This summary is especially useful to map the distribution of the statistical significance of each term across all comparisons, and to present this result graphically with the reString built-in clustermap drawing tool (Supplementary Fig. S11).

**Figure 5:**
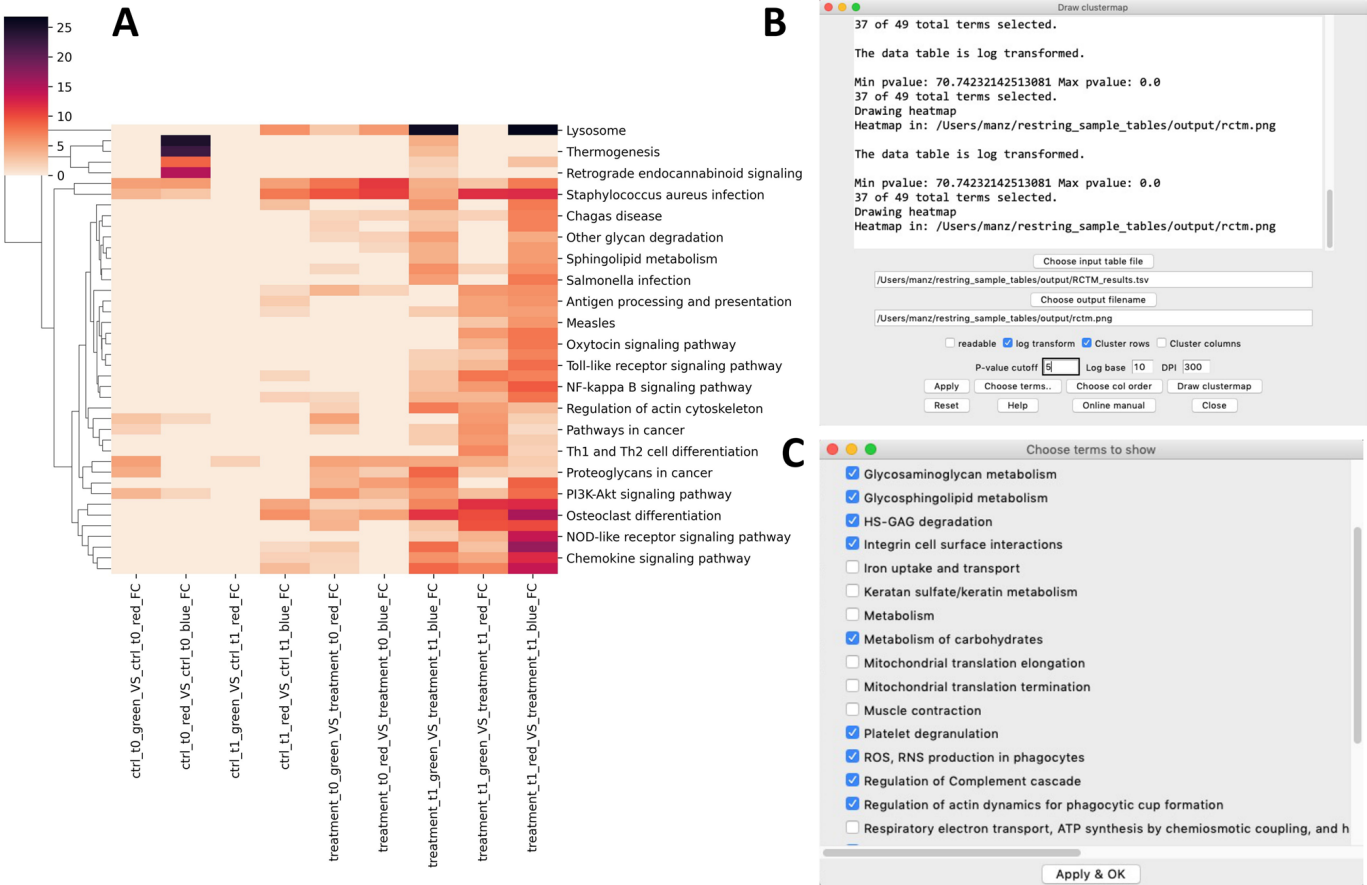
Visualization of a “results”-type table with built-in clustermap tool. The clustermap showing the results for Reactome Pathways obtained with the sample files is shown in (**A**). After running reString with sample input files, it is possible to quickly personalize and draw a clustermap of the results via the built-in clustermap tool (**B**). In this example, the FDR values have been log-transformed, and a cutoff of 5 (corresponding to FDR < 0.00001) has been applied to restrict the plot to the most significant enriched terms. Further, it is possible to remove form the plot terms that are deemed unfit, as shown in (**C**).

An example of a clustermap drawn from aggregated results of Reactome pathways from sample data is shown in Fig. 5. In addition to rapidly conveying the message of which terms are enriched in a particular comparison, with respect to the entirety of experimental conditions, the way reString organizes the functional enrichment files makes it easy to retrieve which genes are contained in a clustermap block. For instance, to investigate the genes related to Immune System term in the Reactome Pathways, it is sufficient to open the corresponding folder of the desired experimental condition (for instance, treatment_t1_red_VS_treatment_t1_blue_FC), and pick the corresponding RCTM tables (Supplementary Fig. S14). Furthermore, if the analysis was run with up- and downregulated genes separately (as per default), it is easy to determine what list the term is contained in.

**Figure 6:**
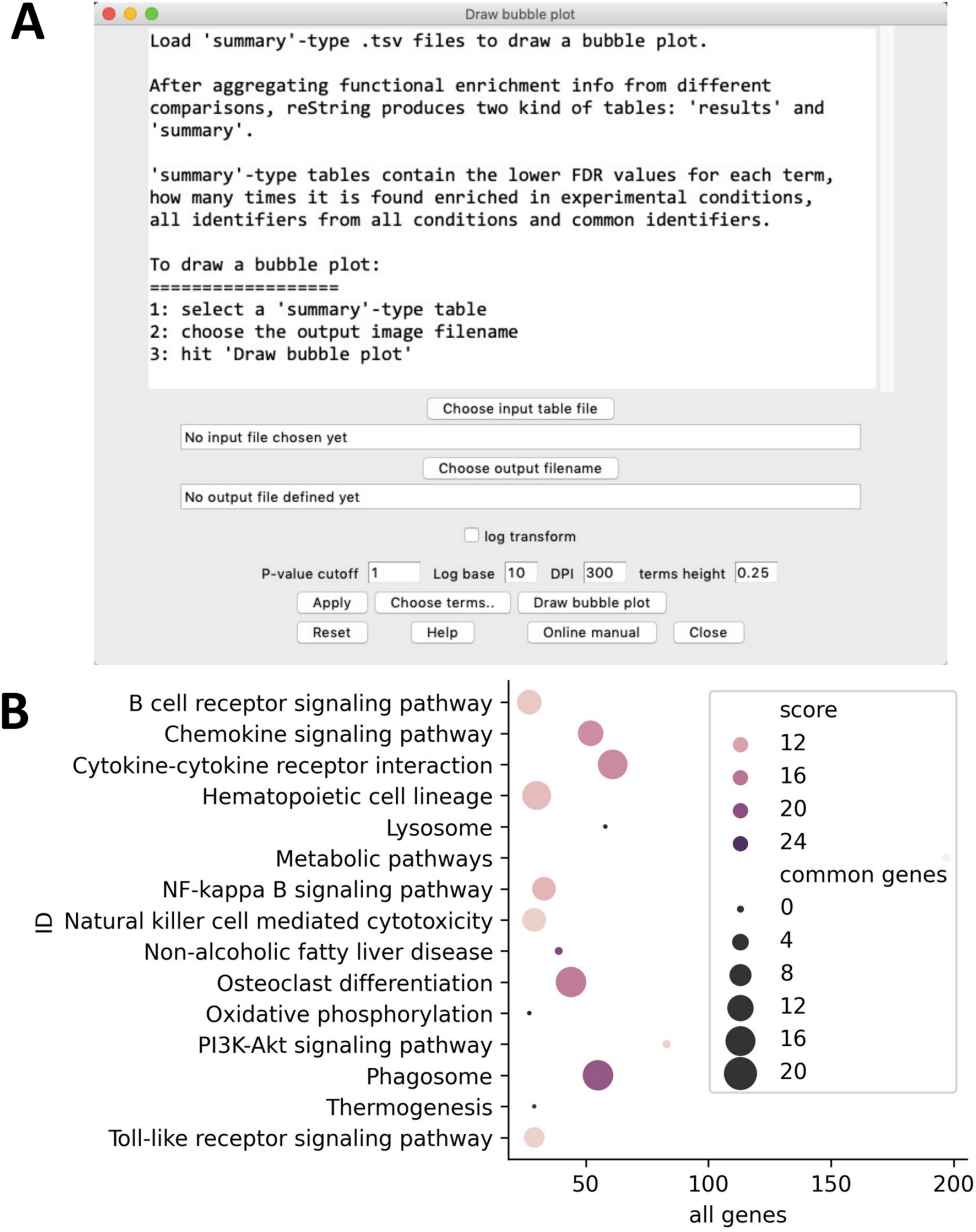
Visualization of a “summary”-type table with built-in bubble plot tool. An example bubble plot showing the results for selected KEGG Pathways is shown in (**A**). After running reString with sample input files, it is possible to quickly personalize and draw a bubble plot of the results via the built-in bubble plot tool (**B**). Similarly to the clustermap tool, customization options are available.

An example of a bubble plot drawn from aggregated results of KEGG pathways is shown in Fig. 6. This kind of visualization helps identifying, at a glance, the terms that contain the highest number of enriched genes, the number of shared genes across considered experimental conditions and the corresponding FDR. This tool, smoothly integrating with the clustermap tool, helps providing a clearclut graphical visualization of relevant information of common elements of the functional enrichment of a large number of experimental conditions.

The software tool described in the present manuscript has already been employed previously^30^. To further demonstrate the usefulness of reString in the handling of functional enrichment results from complex experimental layouts in just a few clicks, we used it to process RNAseq data generated in our laboratory. To this aim, we performed RNAseq on mouse aortas of two different mouse strains, knockout for Apoe (EKO), the gene encoding for apolipoprotein E, and double knockout for Apoe and Apoa1, the gene encoding for apolipoprotein A-I (DKO). Both genotypes are severely dyslipidemic (EKO mice are strongly hyperlipidemic, DKO almost completely lack high-density lipoproteins) and are prone to atherosclerosis development. For each mouse line, the experimental plan envisaged two different time points, and two different dietary treatments, a standard laboratory diet (NLD), extremely low in dietary fats and without cholesterol, or a Western-type diet, enriched in fats and cholesterol (WD—Supplementary Fig. S15).

**Figure 7:**
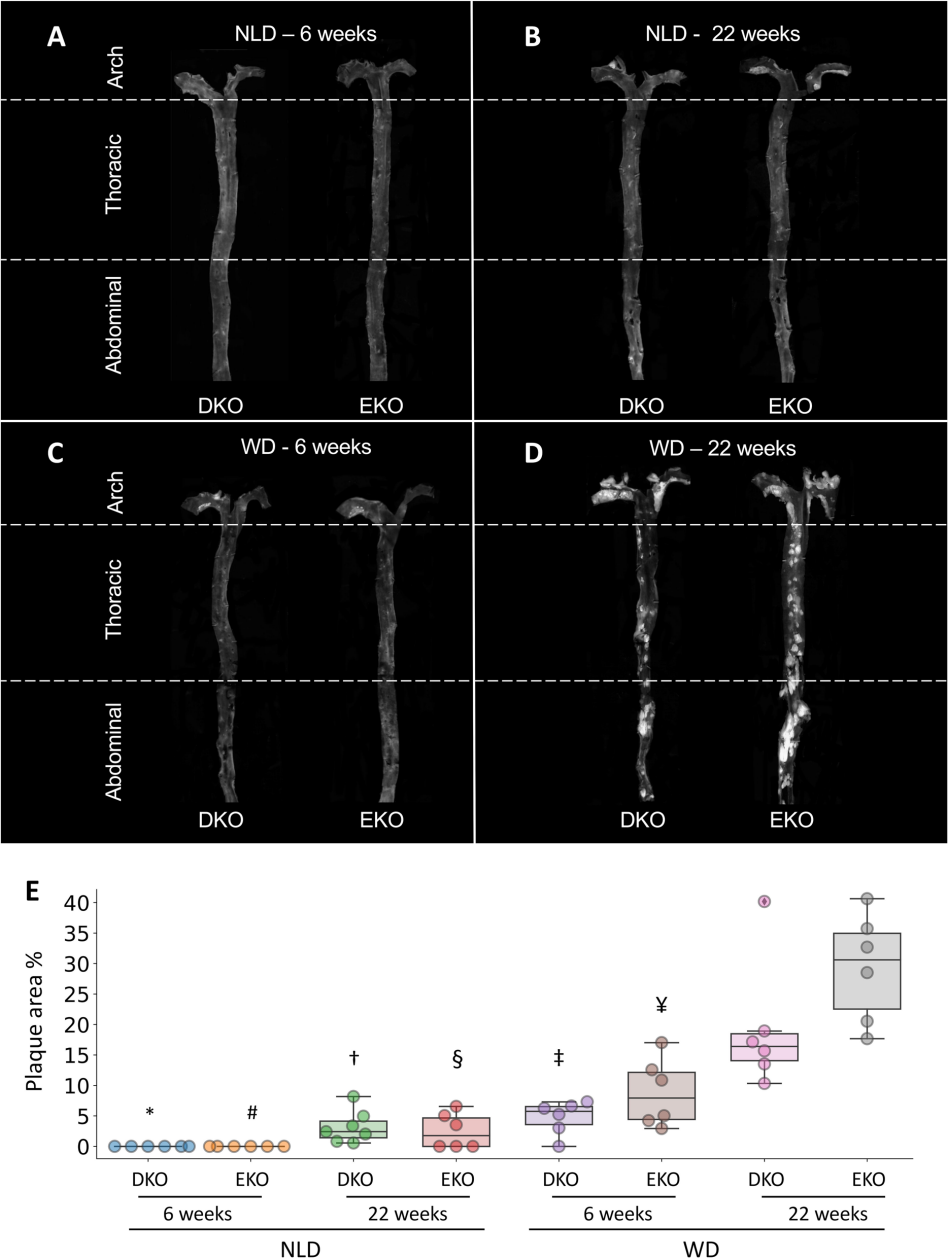
Quantification of aortic plaque area by en-face analysis. At the end of the dietary treatments, whole aortas were collected and the *en-face* analysis was performed to quantify the percentage of aortic surface covered by atherosclerotic plaques (white areas) (**A**–**D**). Data is shown as box plots, with upper and lower ends of the boxes indicating the 25th and 75th percentiles, respectively (**E**). The length of the box shows the interquartile range within which 50% of the values are located. The solid grey lines denote the median. Statistically significant differences were determined with Welch’s ANOVA followed by Dunnett’s post-hoc test, as follows: EKO 22 weeks at WD versus * DKO, 6 weeks NLD (*p* = 0.0065); # EKO, 6 weeks NLD (*p* = 0.0065); † DKO, 22 weeks WD (*p* = 0.0067); § EKO, 22 weeks (*p* = 0.0071); ‡ DKO, 6 weeks WD (*p* = 0.010); ¥ EKO, 6 weeks WD (*p* = 0.025).

Atherosclerosis development was evaluated in the whole aorta. After 6 weeks on NLD, no atherosclerotic plaques were visible in any genotype, whereas on WD both EKO and DKO mice showed initial atherosclerosis development. A comparable plaque development was observed in mice of both genotypes even after 22 weeks at NLD. Twenty-two weeks on WD worsened atherosclerotic plaques, dramatically increasing lesion size in both DKO and EKO mice (Fig. 7 and Supplementary Fig. S16).

**Figure 8:**
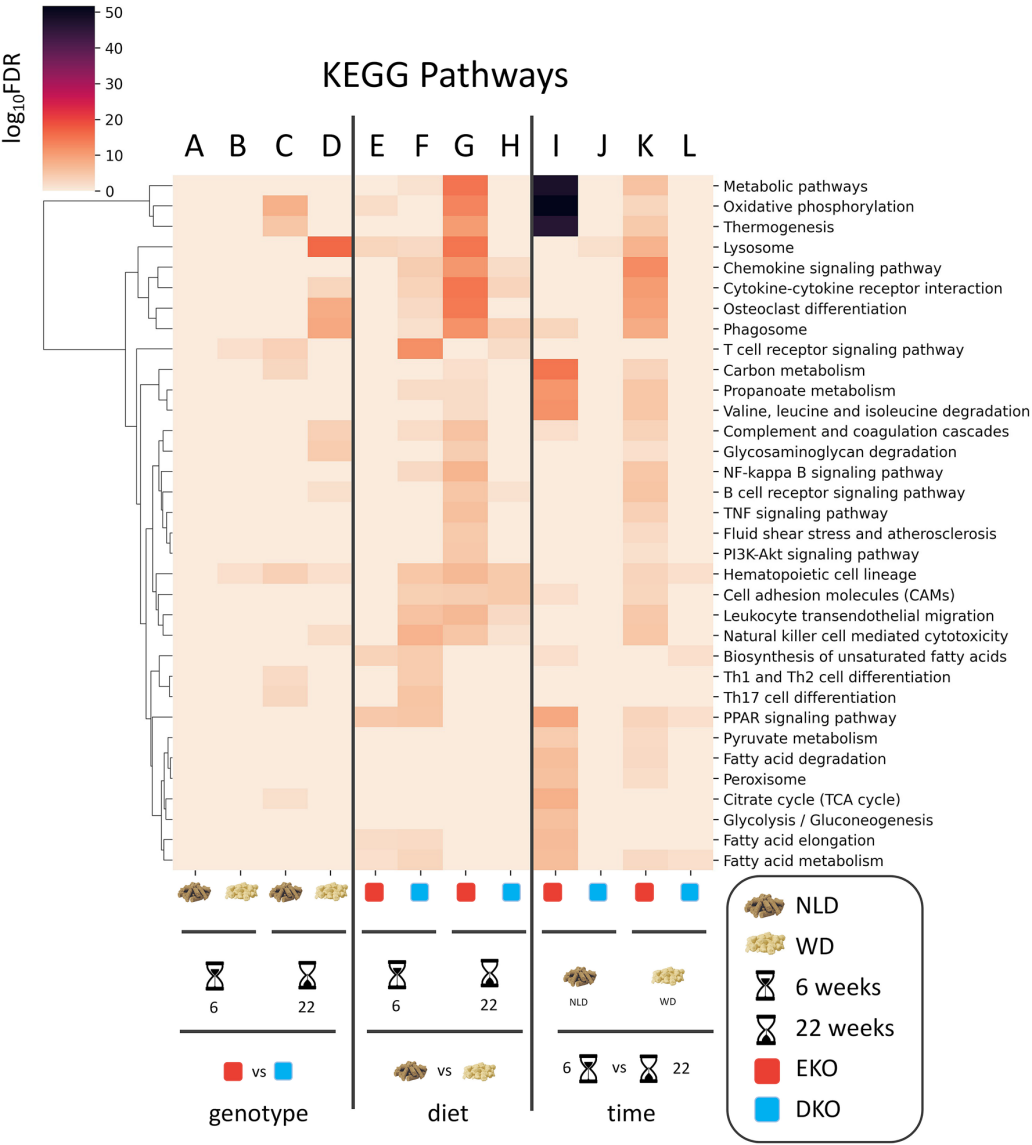
Aggregation of functional enrichment results—KEGG. reString was used to retrieve and process functional enrichment data of from lists of differently expressed genes, taken from multiple one-to-one comparisons. Aggregated data for KEGG Pathways has been processed and customized with reString, and it is shown unmodified from the program (to the exception of the graphical legend, **A**–**L**). Eyeing the clustermap immediately delivers take-away messages that would have been otherwise tricky to obtain, such as: (i) the two genotypes show differences at the latest time point. At WD, pathways show more robust FDR scores (**C**,**D**); (ii) diet differences push changes in gene expression at the latest time point in EKO (**G**) but not in DKO (H); (iii) eating WD rather than NDL drives dramatic changes in the metabolism of EKO at 6 (**I**), but not at 22 weeks (**K**); conversely, the passing of time does yield less dramatic changes in gene expression in DKO (**J**,**L**).

After the necessary bioinformatic analyses (reads QC, trimming, mapping, transcript abundance estimation, etc.), we produced reString-compliant lists of DE genes for twelve comparisons. As an example, aggregated results for KEGG Pathways are shown in Fig. 8. Transcript abundance estimation was validated by quantitative polymerase chain reaction on 6 randomly picked genes and was found to closely match RNA-seq results (Supplementary Fig. S17).

Although there was a comparable plaque development in the two genotypes, the transcriptome analysis revealed different gene expression signatures.

The comparison between genotypes (Fig. 8A–D), in accordance with the absence of plaque development after 6 weeks on NLD, did not reveal significantly enriched pathways between EKO and DKO (Fig. 8A), and showed the greatest differences after 22 weeks of dietary treatments (Fig. 8C,D). The comparison between diets (Fig. 8E–H) clearly showed that DKO had a more pronounced response to dietary treatment after 6 weeks (Fig. 8F), whereas EKO had a massive increase in enriched pathways after 22 weeks on WD (Fig. 8G).

The intra-genotype comparison between time points (Fig. 8I–L) strikingly shows how, albeit with different contributions of the different dietary treatments, there are profound changes in the gene expression profile that are still ongoing in EKO in the transition between 6 and 22 weeks (Fig. 8I,K) that are mostly absent in DKO (Fig. 8J,L). We hypothesize that DKO mice might reach a transcriptional steady state earlier than EKO.

By scrutinizing reString aggregated results (Supplemental Data Sets) we could readily observe interesting trends in some of the pathways that were modulated across all experimental conditions:

*Hematopoietic cell lineage* (mmu04640). The aggregated results showed that the “Hematopoietic cell lineage” was the most influenced pathway, the expression level of its constituent genes being impacted in 8 out of 12 experimental conditions. Compared to DKO, EKO had this pathway enriched at the longest time point, regardless of the diet administered. In EKO, WD administration and the passing of time synergistically enriched this pathway. Conversely, in DKO, WD only increased the expression of these genes mainly after 6 weeks.

*Lysosome* (mmu04142) and *Phagosome* (mmu04145). These pathways were also among the ones mostly affected, being significantly enriched in 6 comparisons. EKO fed WD for the longest time had the highest enrichment of the Lysosome pathway, compared to DKO. Yet, six weeks at WD were already sufficient to change the expression of these genes in both genotypes, although only in EKO the passing of time further enriched it.

Similarly, EKO fed WD for the longest time showed the highest enrichment of the Phagosome pathway, compared to DKO (Fig. 8D). In the diet comparisons, DKO had this pathway enriched at both time points (Fig. 8F,H)—only at the latest for EKO (Fig. 8G); in the time point comparisons, this pathway was enriched only in EKO in both dietary treatments (Fig. 8I,K), with the highest enrichment at WD.

*PPAR signaling pathway* (mmu03320) and *Fatty acid metabolism* (mmu01212). These pathways were enriched in 5 out of 12 comparisons and showed no difference between the two genotypes, when fed the same diet for the same time.

The most striking visual impact of the reString output heatmap is the block containing *Metabolic pathways* (mmu 01100), *Oxidative phosphorylation* (mmu 00190) and *Thermogenesis* (mmu 04714) in the time comparison. At NLD, the enrichment of those pathways in EKO is paramount (Fig. 8I). These pathways are also enriched at WD (Fig. 8K), but their enrichment is lower than at NLD, suggesting that the feeding at WD initiates earlier to modulate the expression of the genes annotated in those pathways, and that the transcriptional equilibrium that is reached at 22 weeks at WD is more similar to the one already established at 6 weeks at WD, than what happens at NLD at later stages. Interestingly, no such thing happens in DKO mice. This finding is further supported by the “immune-metabolic” block of pathways enriched at 22 weeks in EKO in the diet comparison (Fig. 8G), but again not in DKO (Fig. 8H).

In conclusion, we developed reString, a cross-platform software with a graphical user interface, written in Python, to enable all Researchers—especially including those without specific bioinformatics skills—the possibility of broadening the exploration of their RNAseq or high-throughput proteomics datasets, effortlessly automating a series of tasks that would be otherwise daunting if performed by hand. Furthermore, reString is actively maintained. While the tool is feature-complete, we plan to expand its features with new styling/charting options, broaden its support to STRING APIs—especially focusing on the Users’ feedback—and releasing bugfixes. Users can give feedback for troubles they may experience (“Help > Give feedback/Report an Issue” or “Help > Report a Bug”), or actively steer the app’s development by requesting new features (“Help > “Request a new feature”). We will also ensure compatibility with future STRING API versions. Last, reString is an open-source project and anyone with the adequate skill is welcome to peek into, exploit, contribute to and fork the project in order to expand and adapt it.

We detailed and showcased a full reString workflow by using sample data, and further demonstrated its applicability with a new RNAseq dataset.

## Supplementary Information

Supplementary Information 1.

Supplementary Information 2.

## Data Availability

The datasets generated and analysed during the current study are available in the Ncbi GEO repository, and can be accessed at: https://www.ncbi.nlm.nih.gov/geo/query/acc.cgi?&acc=GSE173974.
