# Supplementary material for: reString: an open-source Python software to perform automatic functional enrichment retrieval, results aggregation and data visualization
Source: Sci Rep. 2021 Dec 6;11:23458. doi: 10.1038/s41598-021-02528-0 (PMC8648753; doi:10.1038/s41598-021-02528-0)

Supplemental Material

reString: an open-source Python software to perform automatic functional enrichment retrieval, results aggregation and data visualization

Stefano Manzini[1*], Marco Busnelli[1*], Alice Colombo[1], Elsa Franchi[1], Pasquale Grossano[2] and Giulia Chiesa[1]

[1]Department of Pharmacological and Biomolecular Sciences

Università degli Studi di Milano,

20133 Milano, Italy

[2] Fondazione IRCCS Ca' Granda Ospedale Maggiore Policlinico, Milano

* These authors equally contributed to this work.

Corresponding authors:

Giulia Chiesa, giulia.chiesa@unimi.it

Stefano Manzini stefano.manzini@unimi.it

Supplementary Figure S1

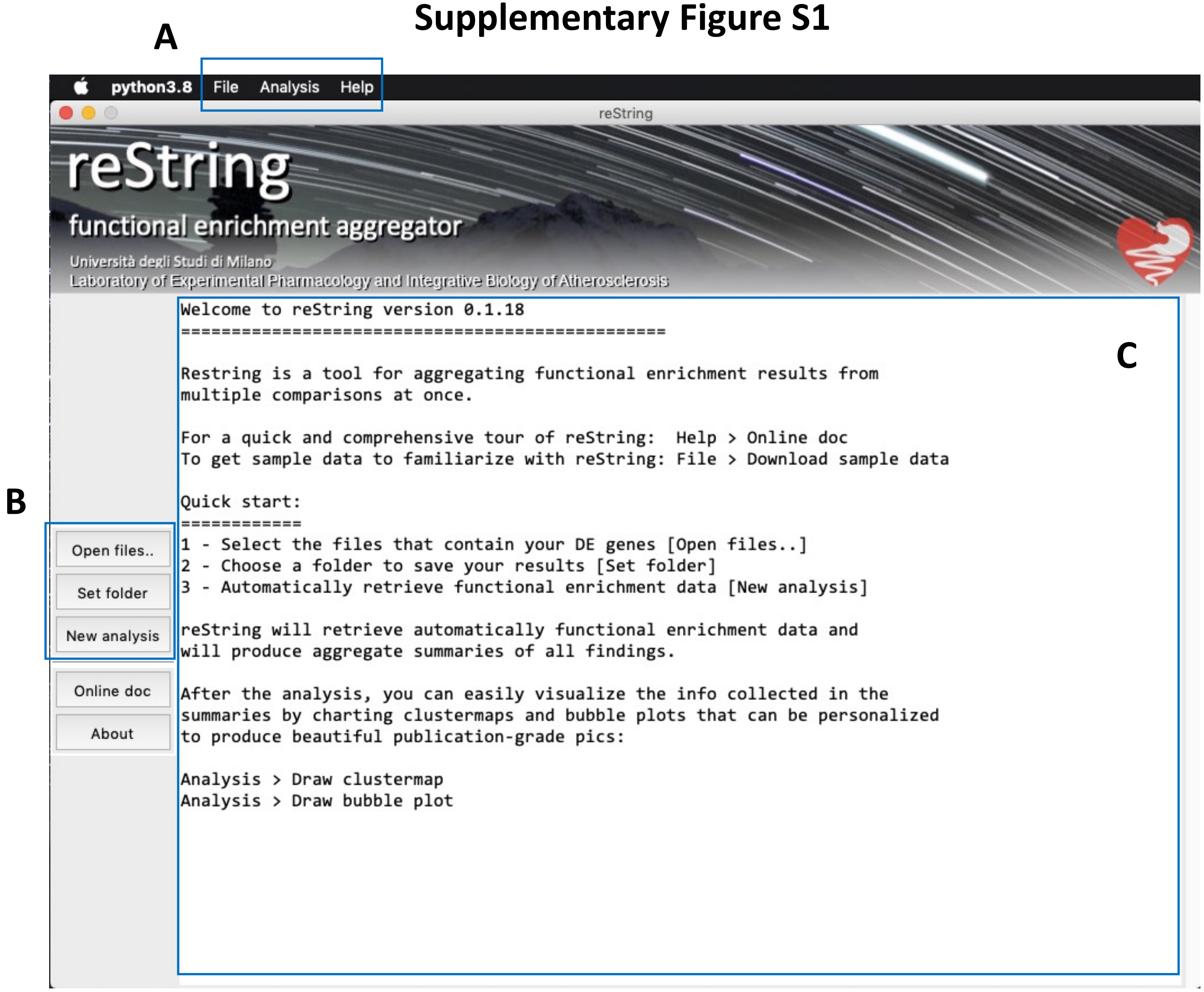

reString main window - This is how reString's main window is rendered in MacOS. `File`, `Analysis` and `Help` menus can be accessed from the top bar (**A**). The buttons on the left (**B**) allow for a quick analysis run, with default settings. The text output frame (**C**) welcomes the User with quick start tips, and is frequently updated in response to the User's inputs.

Supplementary Figure S2

A

B

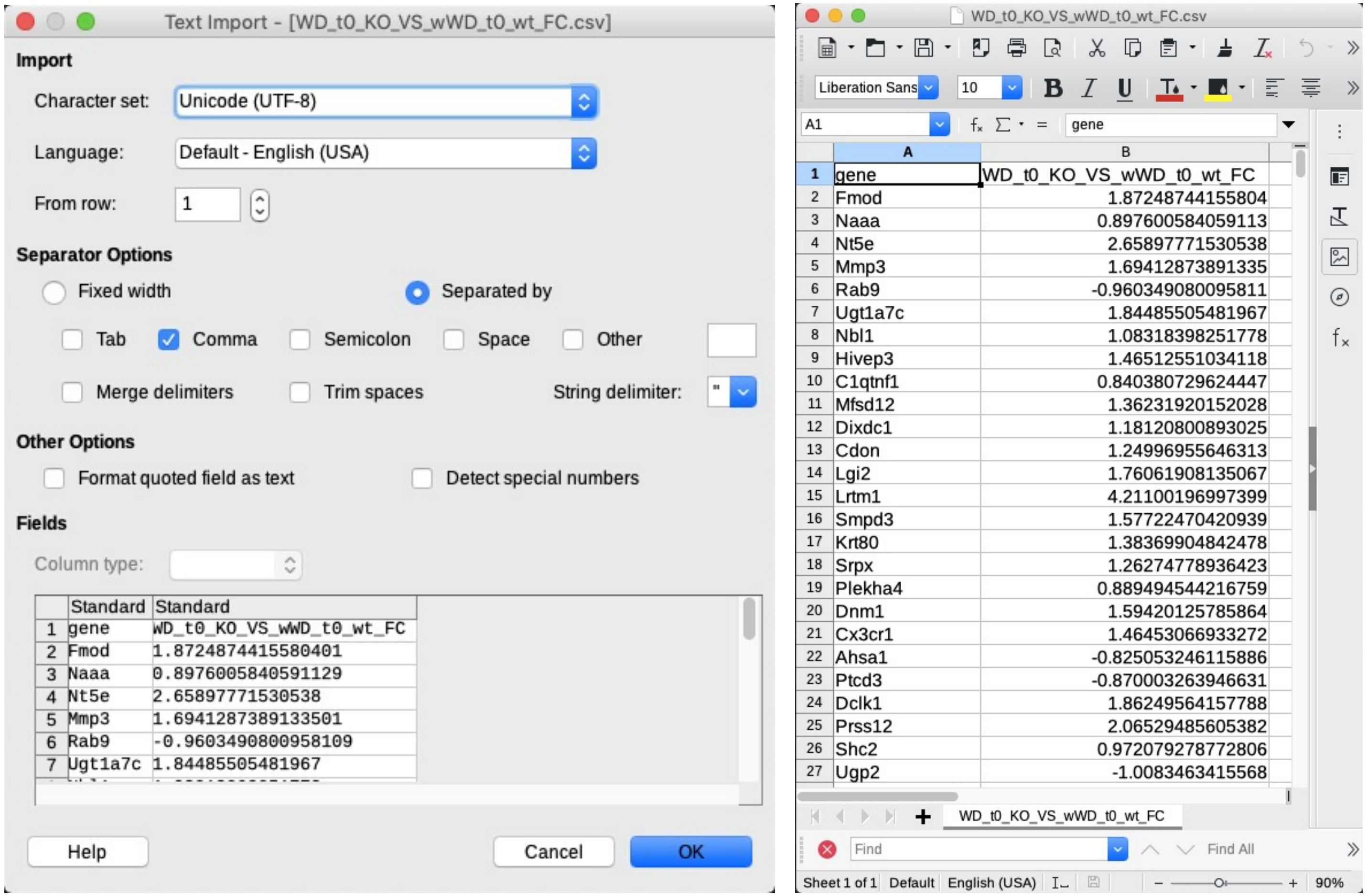

Input files structure – Input files need to be tabular data, in either .csv *(comma separated values)*, .tsv *(tab separated values)* or excel (.xlsx) files.

Please note that the .tdt *(tab delimited text)* extension for tabular data is discouraged as Libre Office's Calc tries to load the data as a Writer document rather than a spreadsheet.

Sample files downloadable from within reString are .csv files that can be seamlessly opened by either Excel or Libre Office's Calc.

In **A** is shown the text import dialog window of Calc. In **B**, the appearance of one file opened in Calc. The input file has two columns: gene (containing the gene IDs) and another labelled as the input filename (containing the fold change of the expression of that gene with respect to the comparison made). Handling RNAseq results to obtain this kind of tables should be straightforward for all researchers.

Even if not all analyses make use the of the fold change information (column 2), input files shall contain it nonetheless.

Supplementary Figure S3

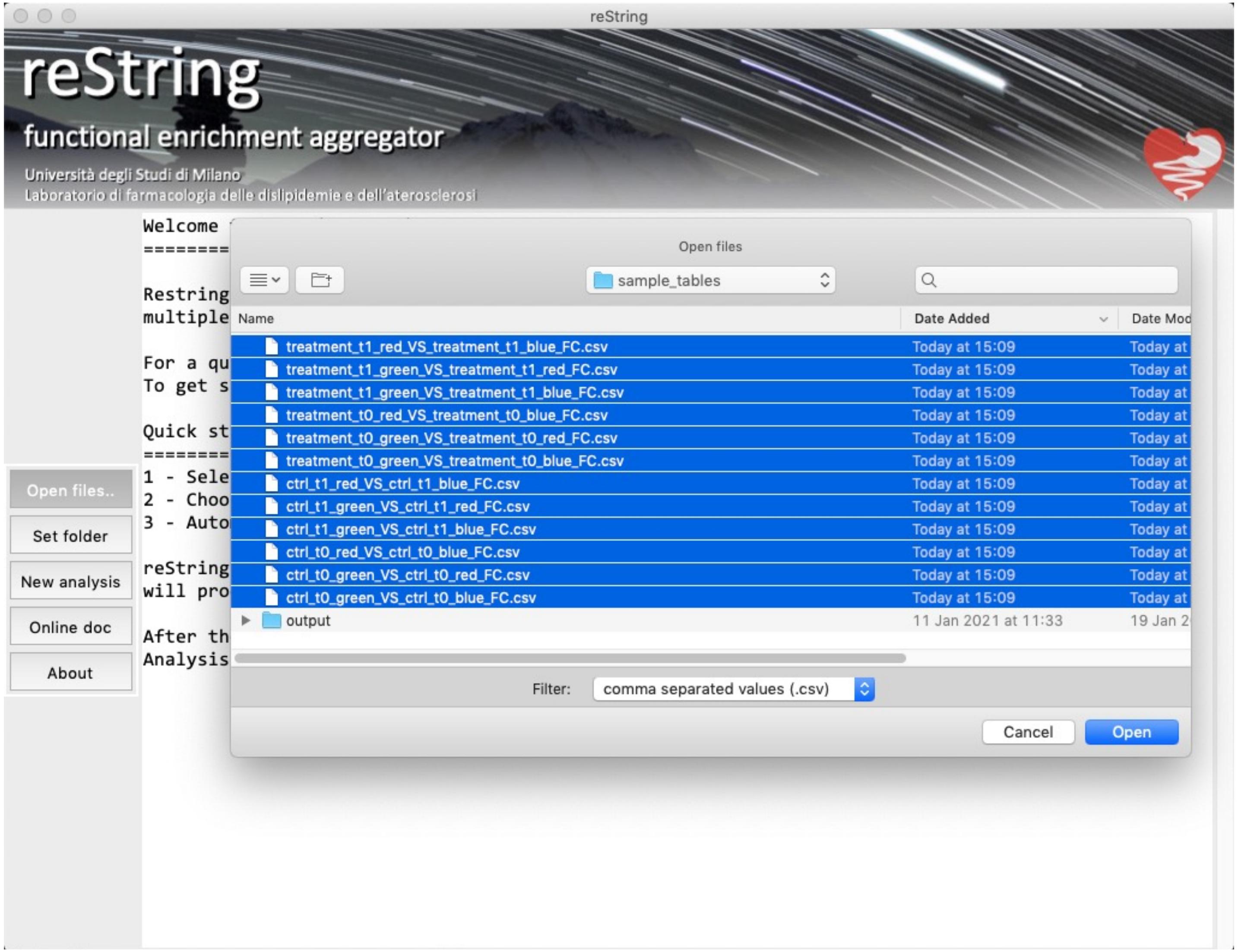

Input files dialog – Choose the files containing the gene lists of interest. Use the `Filter:` to focus on the desired file types in any directory.
Each time the step n. 4 is performed, the input file list is automatically cleared.

Supplementary Figure S4

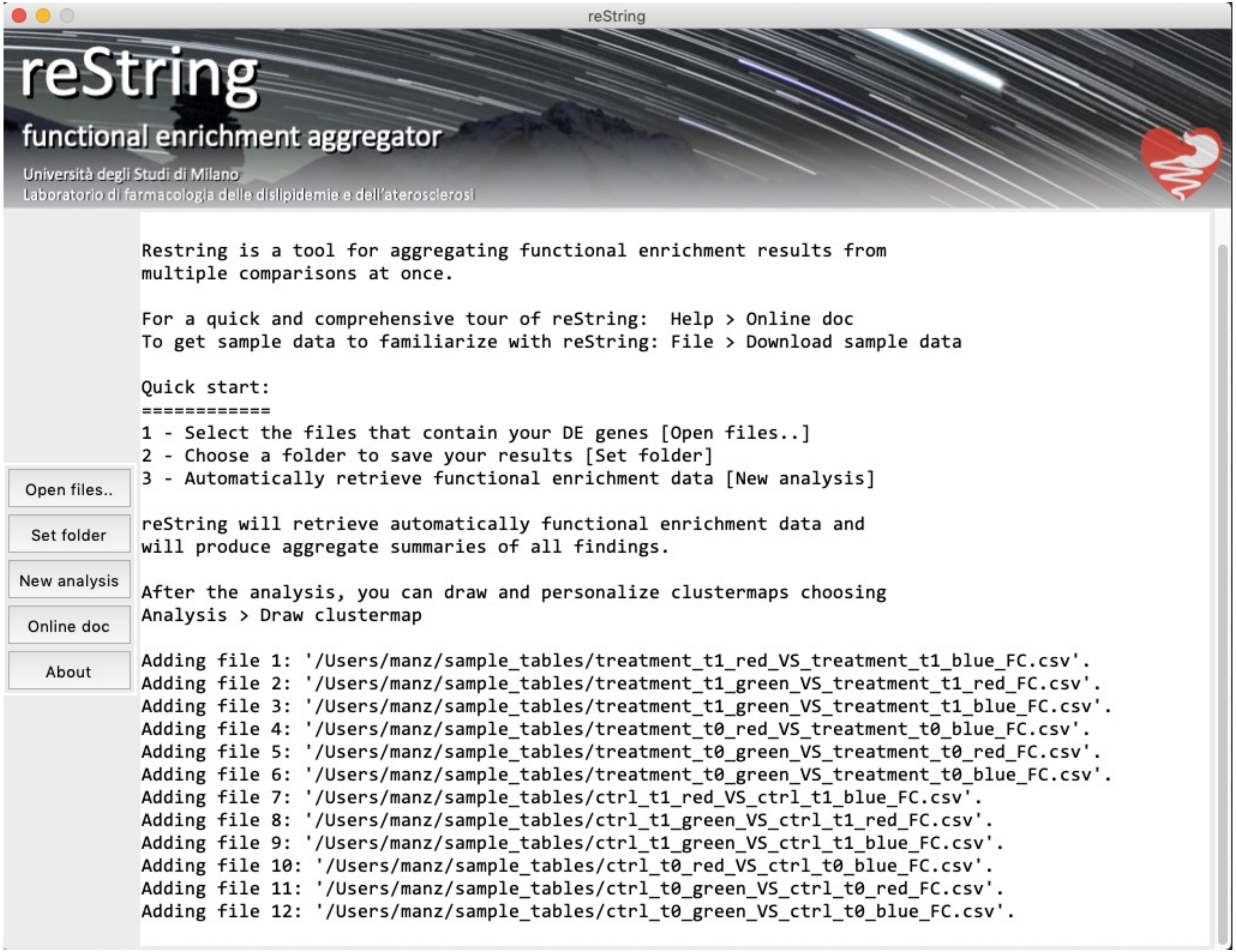

Adding input files – For each successfully loaded input file, reString outputs a message in the textual output frame. The frame is scrollable so that Users can always inspect each step of the analysis.

Supplementary Figure S5

A B

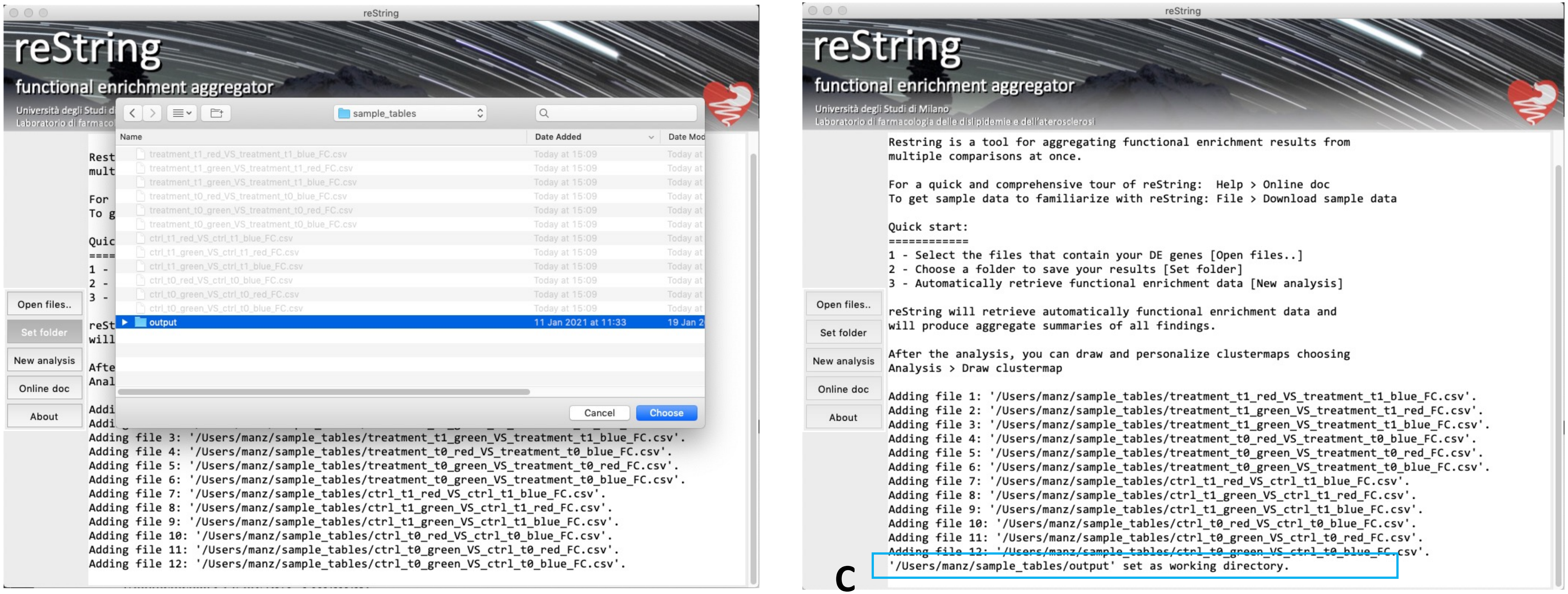

Choose output folder– Choose an existing folder to output the results files to. Navigate the file browser (**A**) to the desired output location and click Choose. The program will notify that the working directory has been set (**B, C**).

Supplementary Figure S6

A

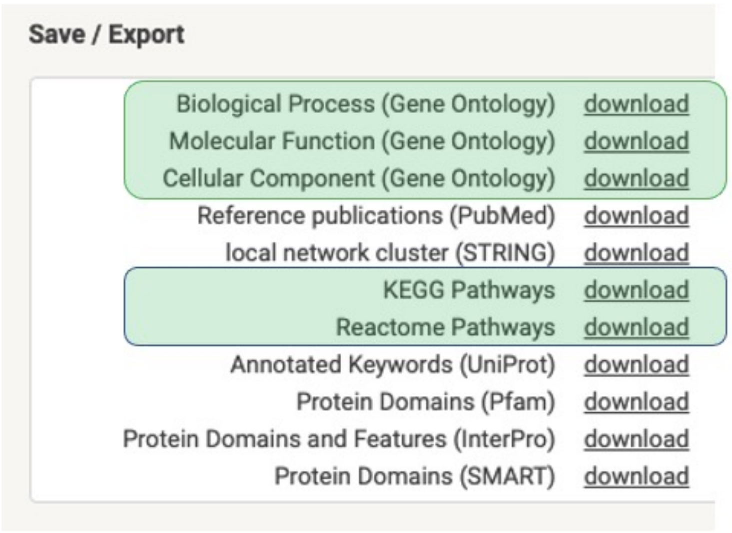

STRING website analysis tools – After performing PPI *(protein-protein interaction)* analysis with STRING, at the bottom of the page, the "`∑ Analysis`" button opens a dialog through which further functional enrichment data can be downloaded (**A**).
As an example, a KEGG enrichment file (saved by STRING as `enrichment.KEGG.tsv`) structure is shown in **B,** as it appears as opened with Libre Office's Calc. "ALL_" was prepended to the filename by reString, as for this particular example the gene list used to retrieve KEGG information contained all genes, irrespective of their up- or down-regulation with respect to experimental groups.

B

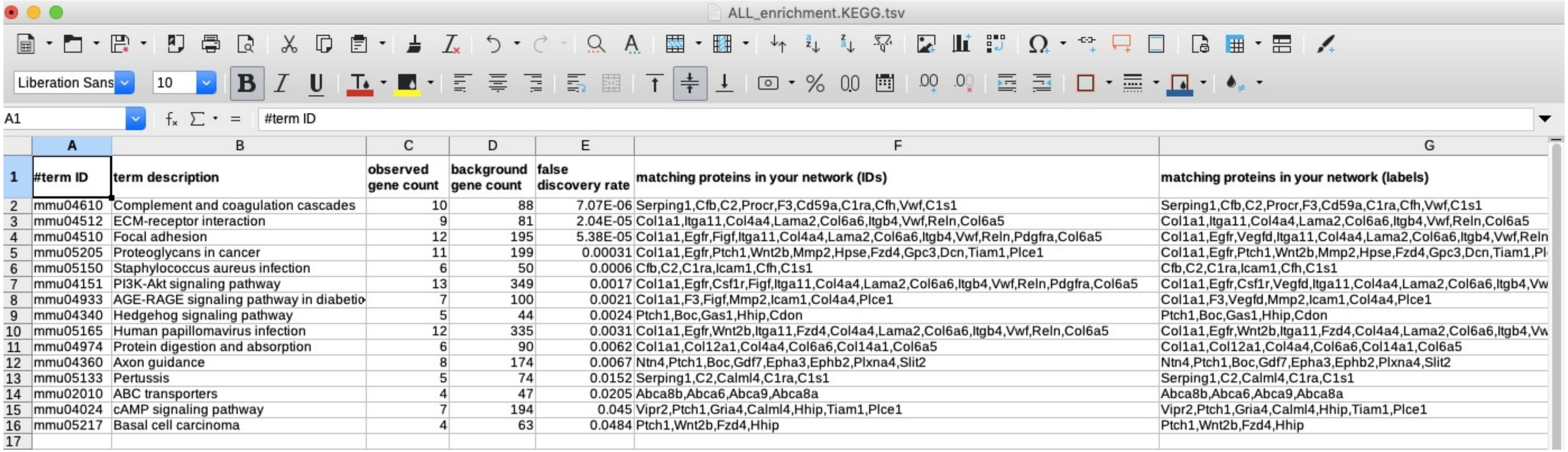

	A	B	C	D	E	F	G
1	#term ID	term description	observed gene count	background gene count	false discovery rate	matching proteins in your network (IDs)	matching proteins in your network (labels)
2	mmu04610	Complement and coagulation cascades	10	88	7.07E-06	Serping1,Cfb,C2,Procr,F3,Cd59a,C1ra,Cfh,Vwf,C1s1	Serping1,Cfb,C2,Procr,F3,Cd59a,C1ra,Cfh,Vwf,C1s1
3	mmu04512	ECM-receptor interaction	9	81	2.04E-05	Col1a1,Itga11,Col4a4,Lama2,Col6a6,Itgb4,Vwf,Reln,Col6a5	Col1a1,Itga11,Col4a4,Lama2,Col6a6,Itgb4,Vwf,Reln,Col6a5
4	mmu04510	Focal adhesion	12	195	5.38E-05	Col1a1,Egfr,Figf,Itga11,Col4a4,Lama2,Col6a6,Itgb4,Vwf,Reln,Pdgfra,Col6a5	Col1a1,Egfr,Vegfd,Itga11,Col4a4,Lama2,Col6a6,Itgb4,Vwf,Reln
5	mmu05205	Proteoglycans in cancer	11	199	0.00031	Col1a1,Egfr,Ptch1,Wnt2b,Mmp2,Hpse,Fzd4,Gpc3,Dcn,Tiam1,Plce1	Col1a1,Egfr,Ptch1,Wnt2b,Mmp2,Hpse,Fzd4,Gpc3,Dcn,Tiam1,Pl
6	mmu05150	Staphylococcus aureus infection	6	50	0.0006	Cfb,C2,C1ra,Icam1,Cfh,C1s1	Cfb,C2,C1ra,Icam1,Cfh,C1s1
7	mmu04151	PI3K-Akt signaling pathway	13	349	0.0017	Col1a1,Egfr,Csf1r,Figf,Itga11,Col4a4,Lama2,Col6a6,Itgb4,Vwf,Reln,Pdgfra,Col6a5	Col1a1,Egfr,Csf1r,Vegfd,Itga11,Col4a4,Lama2,Col6a6,Itgb4,Vw
8	mmu04933	AGE-RAGE signaling pathway in diabetic	7	100	0.0021	Col1a1,F3,Figf,Mmp2,Icam1,Col4a4,Plce1	Col1a1,F3,Vegfd,Mmp2,Icam1,Col4a4,Plce1
9	mmu04340	Hedgehog signaling pathway	5	44	0.0024	Ptch1,Boc,Gas1,Hhip,Cdon	Ptch1,Boc,Gas1,Hhip,Cdon
10	mmu05165	Human papillomavirus infection	12	335	0.0031	Col1a1,Egfr,Wnt2b,Itga11,Fzd4,Col4a4,Lama2,Col6a6,Itgb4,Vwf,Reln,Col6a5	Col1a1,Egfr,Wnt2b,Itga11,Fzd4,Col4a4,Lama2,Col6a6,Itgb4,Vw
11	mmu04974	Protein digestion and absorption	6	90	0.0062	Col1a1,Col12a1,Col4a4,Col6a6,Col14a1,Col6a5	Col1a1,Col12a1,Col4a4,Col6a6,Col14a1,Col6a5
12	mmu04360	Axon guidance	8	174	0.0067	Ntn4,Ptch1,Boc,Gdf7,Epha3,Ephb2,Plxna4,Slit2	Ntn4,Ptch1,Boc,Gdf7,Epha3,Ephb2,Plxna4,Slit2
13	mmu05133	Pertussis	5	74	0.0152	Serping1,C2,Calml4,C1ra,C1s1	Serping1,C2,Calml4,C1ra,C1s1
14	mmu02010	ABC transporters	4	47	0.0205	Abca8b,Abca6,Abca9,Abca8a	Abca8b,Abca6,Abca9,Abca8a
15	mmu04024	cAMP signaling pathway	7	194	0.045	Vipr2,Ptch1,Gria4,Calml4,Hhip,Tiam1,Plce1	Vipr2,Ptch1,Gria4,Calml4,Hhip,Tiam1,Plce1
16	mmu05217	Basal cell carcinoma	4	63	0.0484	Ptch1,Wnt2b,Fzd4,Hhip	Ptch1,Wnt2b,Fzd4,Hhip
17							

Supplementary Figure S7

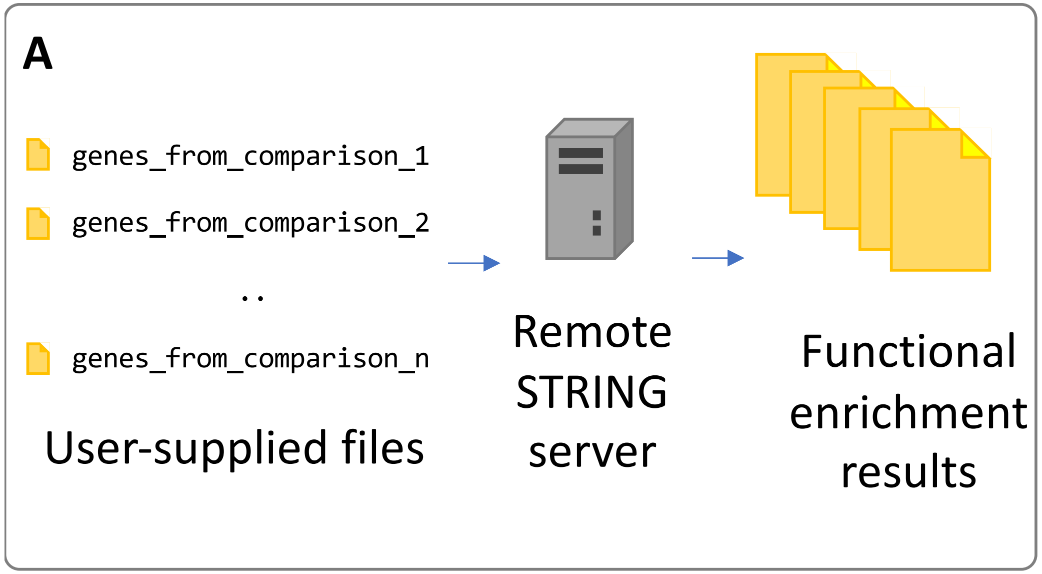

B

```
===============================================================
Aggregation started.
Now in: /Users/manz/sample_tables/output/treatment_t1_red_VS_treatment_t1_blue_FC
Reading from: /Users/manz/sample_tables/treatment_t1_red_VS_treatment_t1_blue_FC.csv
Querying STRING. Session ID: restring-ko47u7au, TaxID: 10090, 420 genes/proteins.
STRING replied in 10568.59 milliseconds.
Table written: UP_enrichment.Component.tsv
Table written: UP_enrichment.Function.tsv
Table written: UP_enrichment.KEGG.tsv
Table written: UP_enrichment.Process.tsv
Table written: UP_enrichment.RCTM.tsv
Querying STRING. Session ID: restring-ko47u7au, TaxID: 10090, 1236 genes/proteins.
STRING replied in 14024.62 milliseconds.
Table written: DOWN_enrichment.Component.tsv
Table written: DOWN_enrichment.Function.tsv
Table written: DOWN_enrichment.KEGG.tsv
Table written: DOWN_enrichment.Process.tsv
Table written: DOWN_enrichment.RCTM.tsv
```

Retrieval of functional enrichment information from STRING. For each supplied gene list table, reString contacts STRING remote servers via STRING APIs to fetch functional enrichment information tables (**A**). reString outputs what it is doing to the textual output frame of the main program's window (**B**).

When running the sample protocol, reString starts with the first file, and creates a subfolder with the same name into the specified results folder. Then, depending on the analysis parameters, it queries STRING for functional information with either all genes (ALL), or upregulated (UP) and/or downregulated (DOWN) ones, naming output tables accordingly *(if the gene list is long enough to generate statistically significant output data)*. A table for each one of KEGG, Function, Component, Process and RCTM results type is retrieved. The process is repeated for all files.

In the example depicted (**B**), the genes contained in the input file were divided into up- and downregulated, and each list was used to probe the STRING server. reString outputs the time that took the server to respond. Following STRING APIs instructions, each request is followed by a pause time of 1 second.

To report itself to the STRING server, reString generates a session-unique session ID, shown at each query.

Supplementary Figure S8

A

```
==================================================================
Finished making functional enrichment tables.
210.99 seconds elapsed.
Getting directories to process.
*Python*: dirs = get_dirs()

Aggregating data for: Component
==================================
*Python*: db = aggregate_results(dirs, kind='Component')
Start walking the directory structure.

Parameters
----------
folders: 12
kind=Component
directions=['UP', 'DOWN']

Processing directory: ctrl_t0_green_VS_ctrl_t0_blue_FC
Processing directory: ctrl_t0_green_VS_ctrl_t0_red_FC
        Processing file DOWN_enrichment.Component.tsv
        Processing file UP_enrichment.Component.tsv
Processing directory: ctrl_t0_red_VS_ctrl_t0_blue_FC
        Processing file DOWN_enrichment.Component.tsv
        Processing file UP_enrichment.Component.tsv
Processing directory: ctrl_t1_green_VS_ctrl_t1_blue_FC
Processing directory: ctrl_t1_green_VS_ctrl_t1_red_FC
        Processing file DOWN_enrichment.Component.tsv
        Processing file UP_enrichment.Component.tsv
```

B

```
Processed 12 directories and 13 files.
Found a total of 26 RCTM elements.
*Python*: tableize_aggregated(db)
*Python*: df.to_csv('RCTM_results.tsv')
*Python*: res = summary(db)
*Python*: res.to_csv('RCTM_summary.tsv')

==================================================================
Finished aggregating all terms. Tables produced:
Component_results.tsv
Component_summary.tsv
Function_results.tsv
Function_summary.tsv
KEGG_results.tsv
KEGG_summary.tsv
Process_results.tsv
Process_summary.tsv
RCTM_results.tsv
RCTM_summary.tsv
```

Aggregation of functional enrichment results. After fetching all tables from STRING, reString begins aggregating data from them, detailing each step to the main window (shown in **A**).
When possible, the equivalent Python code of what it is doing is outputted as well, and it should serve as a reference to whomever would like to reproduce or integrate the same analysis when using reString as a Python module (prefixed by "`*Python*`").
reString processed all the folders it encounters in the output folder indicated before the beginning of the analysis, produced during the retrieval of data from STRING servers.
For each `kind` of term retrieved (KEGG, Function, Component, Process and RCTM), the aggregation is started following the chosen analysis type. In the depicted example, the default analysis is run (indicated with "`directions`").
After completing the aggregation, reString outputs final, aggregated summaries it produced for each `term` (**B**). For each one, two types of tables are produced: **results** and **summary**.

Supplementary Figure S9

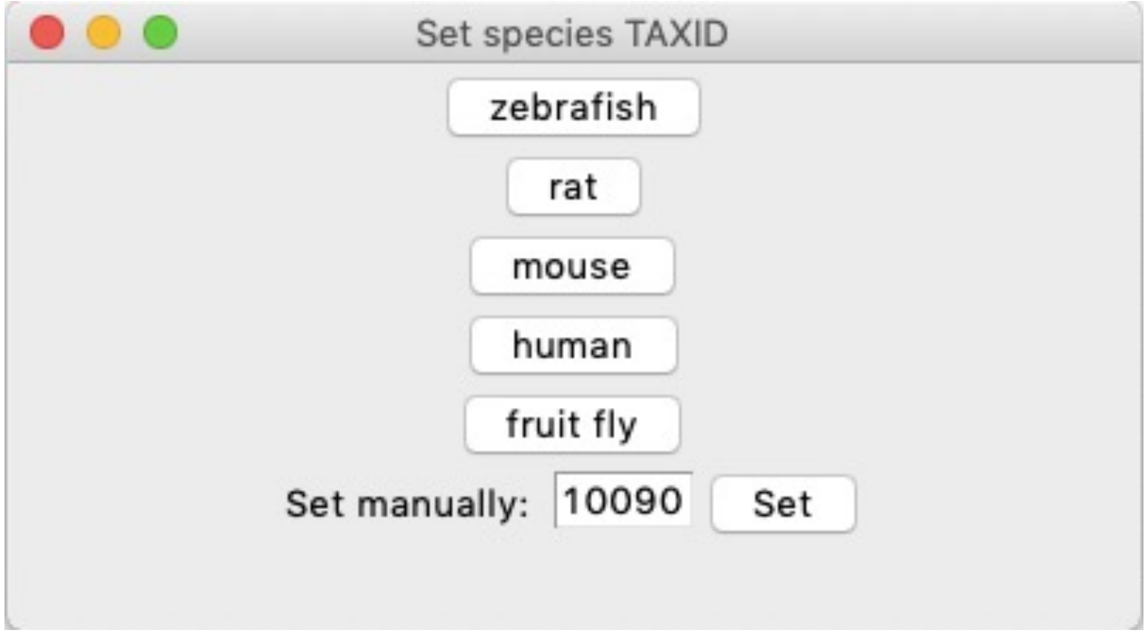

Species selection dialog. STRING requires a species parameter to be indicated when performing PPI network and functional enrichment analysis. reString defaults to mouse (taxonomy identifier 10900). It is possible to choose among the most common species by clicking the corresponding buttons, or directly setting one species by inputting the corresponding numerical taxonomy identified and clicking “`Set`”.

Supplementary Figure S10

A

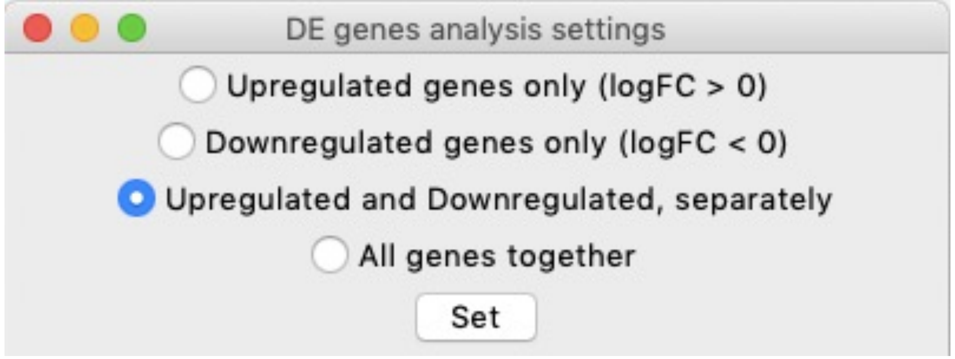

B

gene ID	condition 1	condition 2	condition 1 / condition 2	log2FC
gene 1	143	748	0.191	-2.38
gene 2	50	4	12.5	3.64

Analysis options. reString knows from the input files if the genes of any given comparison are more expressed in one condition with respect to the other. This directionality information can be exploited to set four possible analysis types (**A**). "up" and "down" follows in reString the convention specified in **B**. Researchers should adjust their input data so that "upregulated" or "downregulated" matches the inplied convention.

In the example shown, condition 1 and condition 2 are two experimental conditions where the abundance of the transcript has been estimated. The log_2 of the ratio of the two conditions is calculated as shown, and the following applies:

Upregulated means higher in condition 1 vs condition 2. That is the case of gene 2. The Log2FC is > 0.

Downregulated means lower in condition 1 vs condition 2. That is the case of gene 1. The Log2FC is < 0.

Supplementary Figure S11

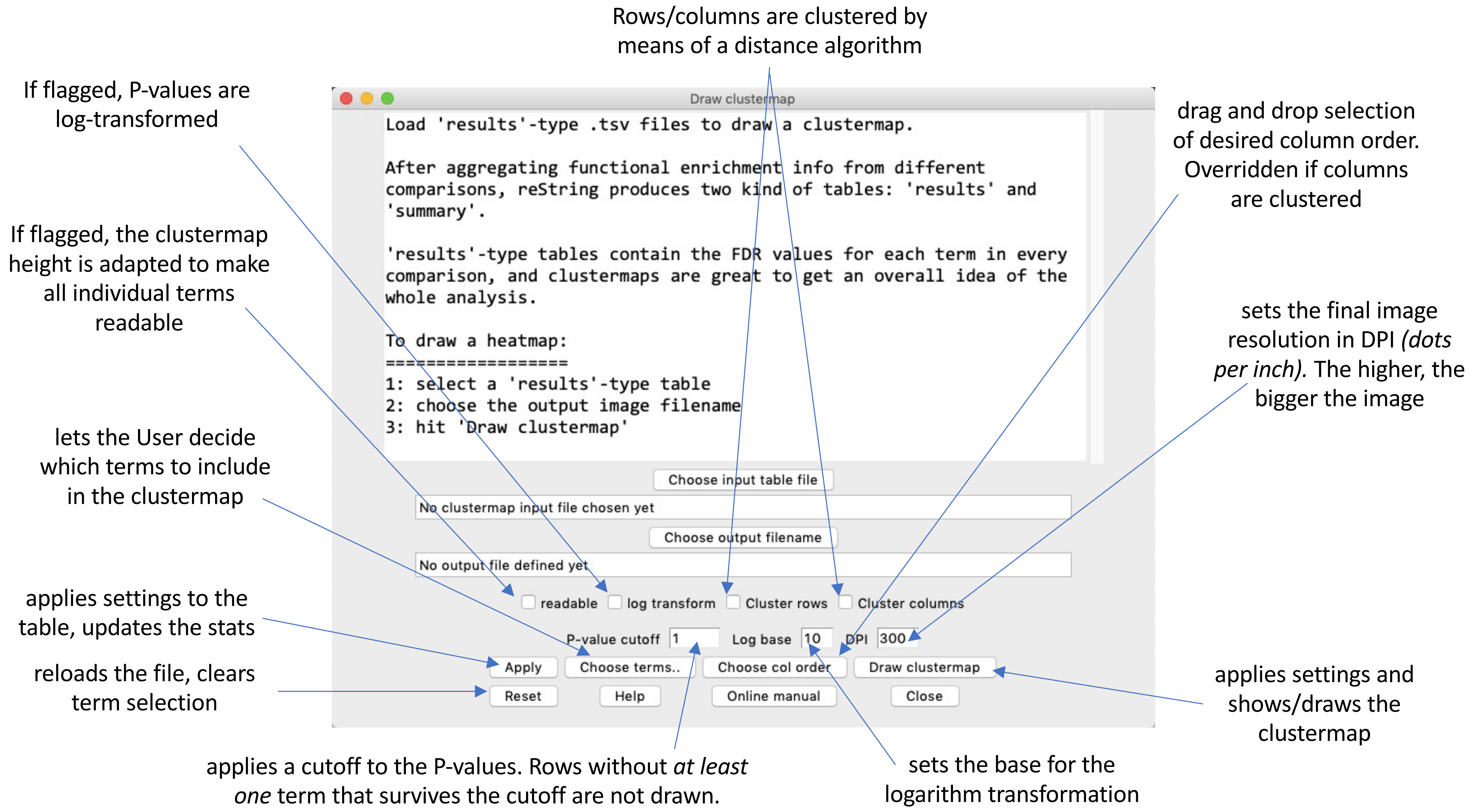

Draw clustermap window. reString has a built-in tool that produces clustermaps by charting **results**-type tables. By default, it shows and saves to a preferred location a heatmap (no clustering of rows and columns) that fits into a reasonably-sized picture. Options are illustrated in the picture above, please refer to the manual for a complete explanation.

Supplementary Figure S12

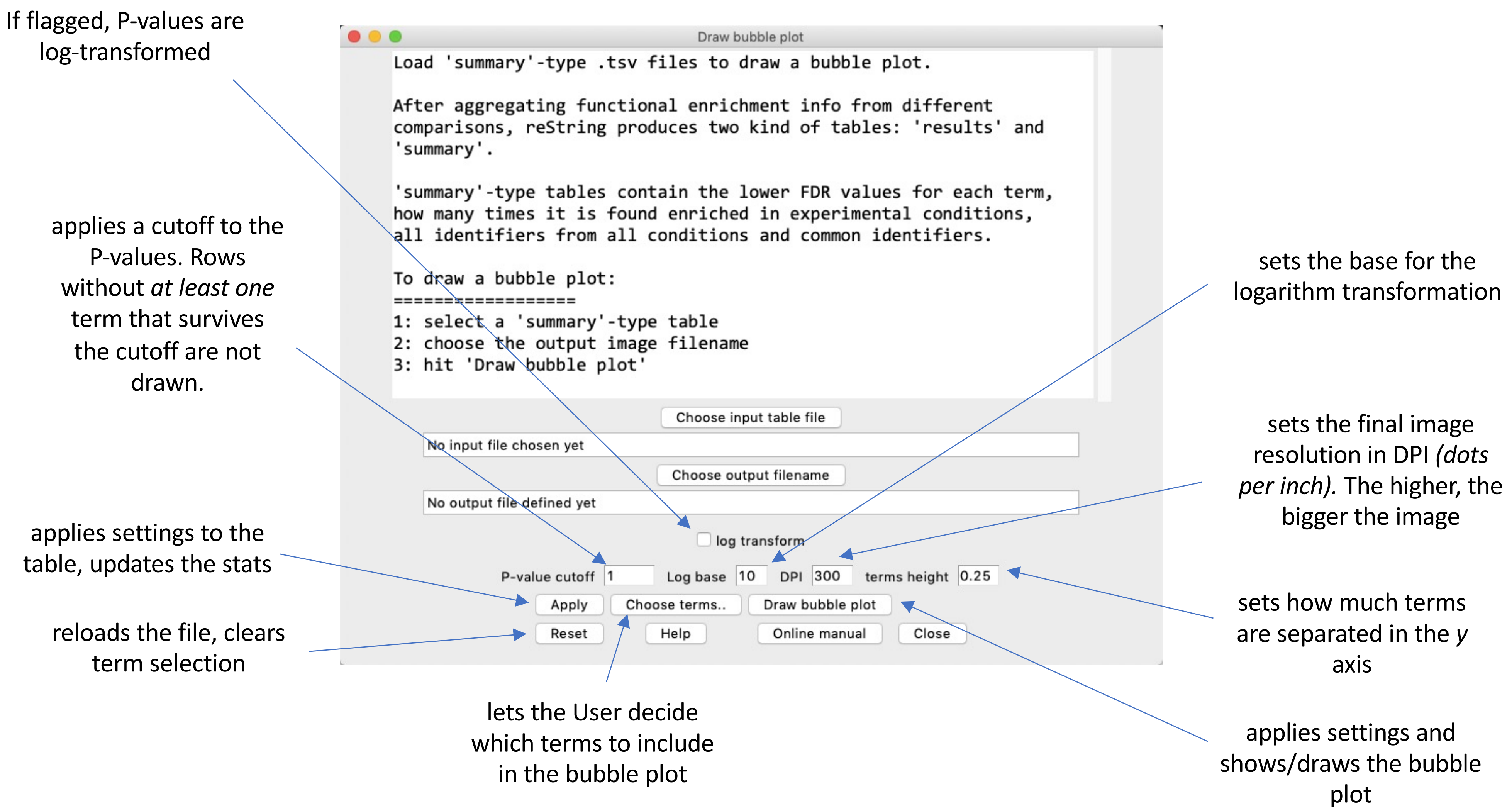

Draw bubble plot window. reString has a built-in tool that produces bubble plots by charting **summary**-type tables. Options are illustrated in the picture above, please refer to the manual for a complete explanation.

Supplementary Figure S13

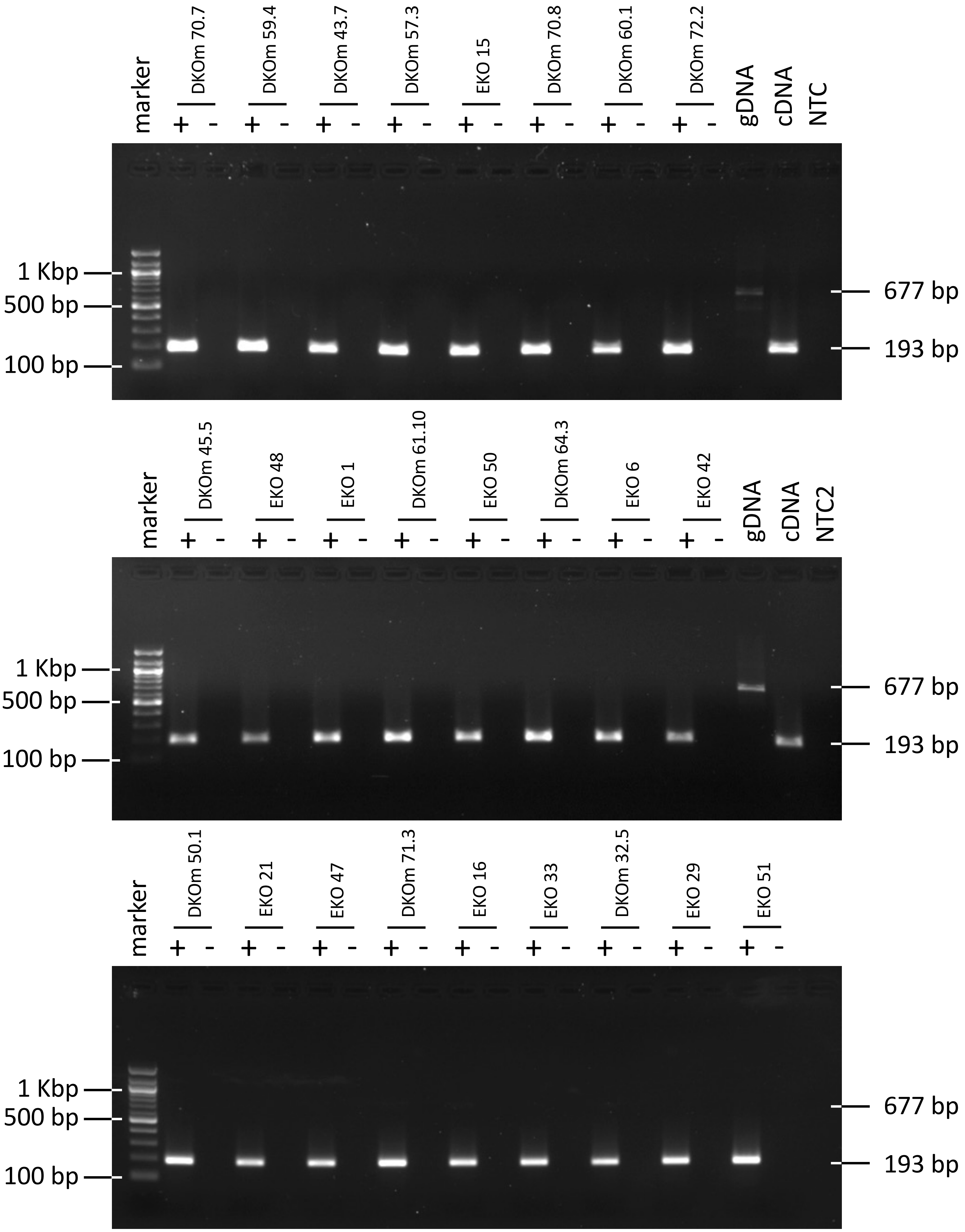

Retrotranscription and DNA contamination test. Specific primers were used to amplify sequences of Srp14 on cDNA (193bp) and gDNA (677) on RT plus and RT minus samples.

Supplementary Figure S14

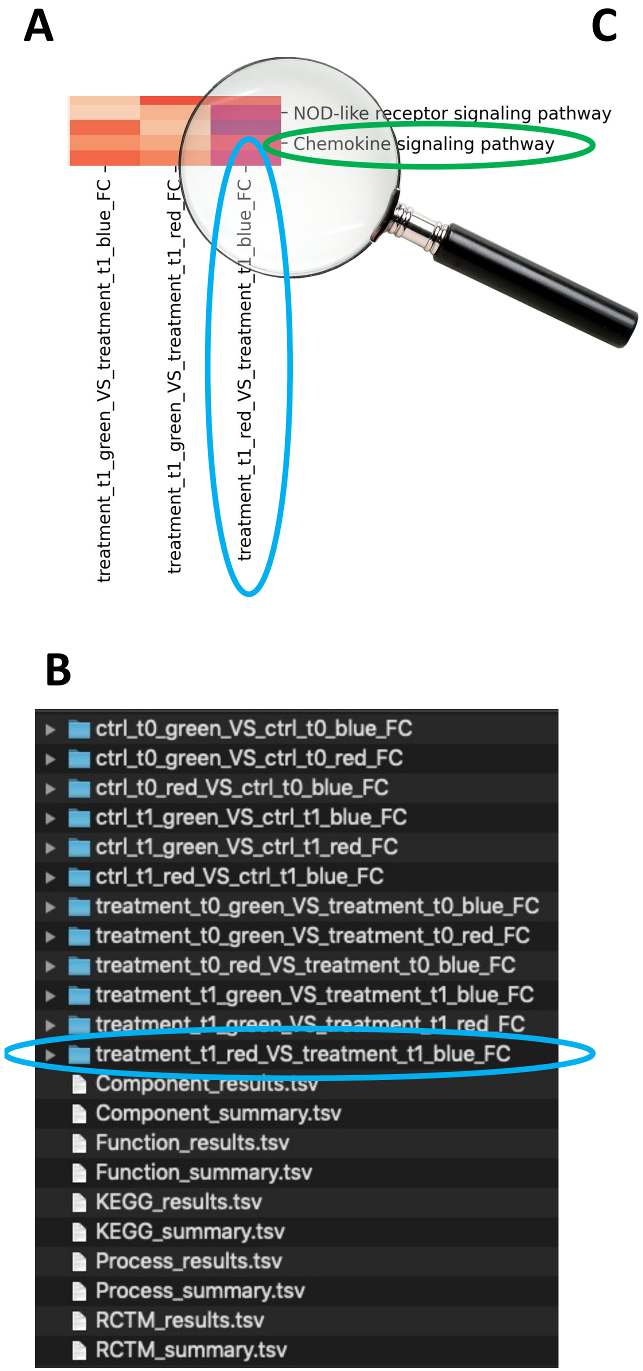

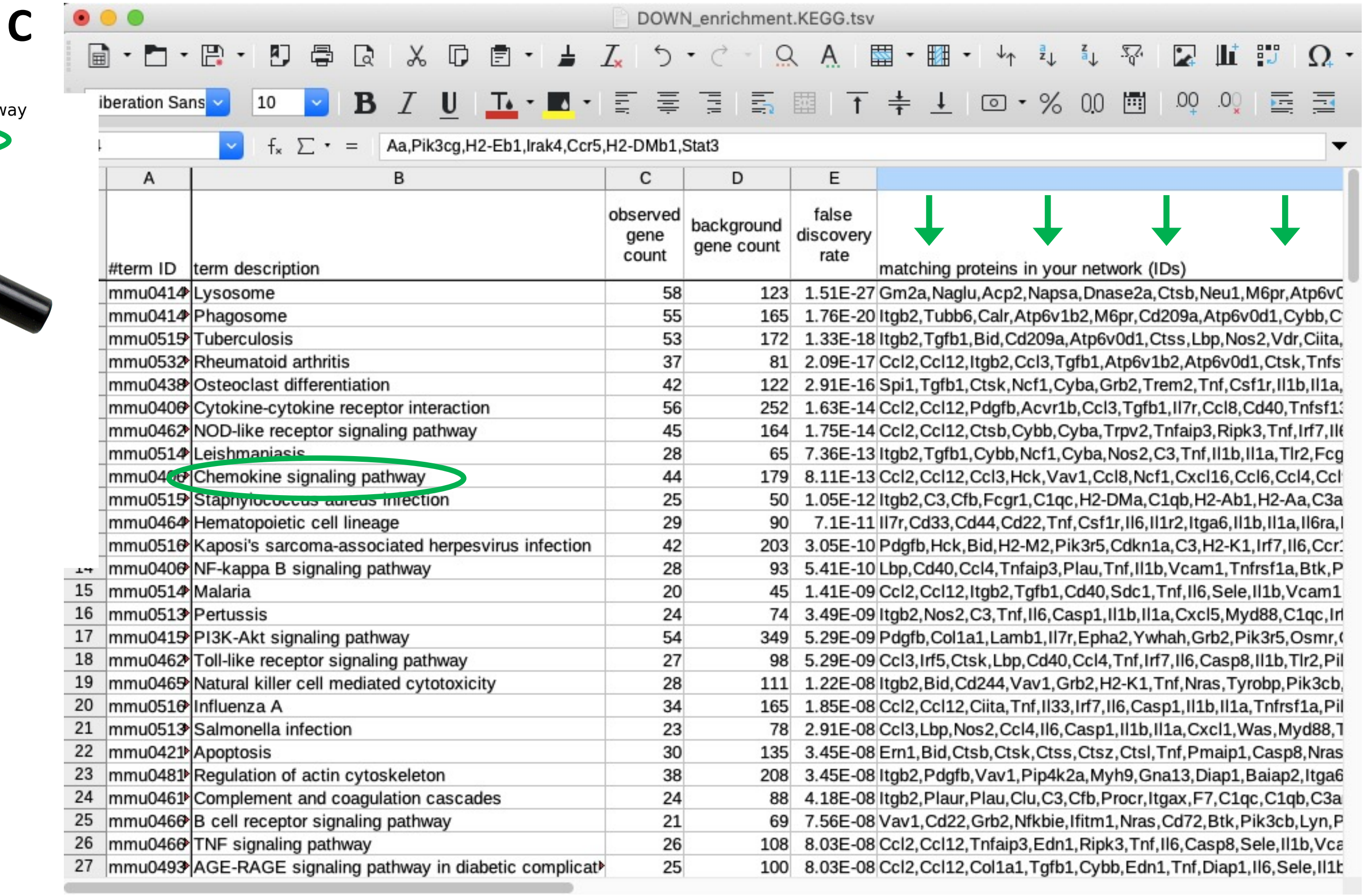

	A	B	C	D	E	
	#term ID	term description	observed gene count	background gene count	false discovery rate	matching proteins in your network (IDs)
	mmu0414	Lysosome	58	123	1.51E-27	Gm2a,Naglu,Acp2,Napsa,Dnase2a,Ctsb,Neu1,M6pr,Atp6v0
	mmu0414	Phagosome	55	165	1.76E-20	Itgb2,Tubb6,Calr,Atp6v1b2,M6pr,Cd209a,Atp6v0d1,Cybb,C
	mmu0515	Tuberculosis	53	172	1.33E-18	Itgb2,Tgfb1,Bid,Cd209a,Atp6v0d1,Ctss,Lbp,Nos2,Vdr,Ciita,
	mmu0532	Rheumatoid arthritis	37	81	2.09E-17	Ccl2,Ccl12,Itgb2,Ccl3,Tgfb1,Atp6v1b2,Atp6v0d1,Ctsk,Tnfs
	mmu0438	Osteoclast differentiation	42	122	2.91E-16	Spi1,Tgfb1,Ctsk,Ncf1,Cyba,Grb2,Trem2,Tnf,Csf1r,Il1b,Il1a,
	mmu0406	Cytokine-cytokine receptor interaction	56	252	1.63E-14	Ccl2,Ccl12,Pdgfb,Acvr1b,Ccl3,Tgfb1,Il7r,Ccl8,Cd40,Tnfsf1
	mmu0462	NOD-like receptor signaling pathway	45	164	1.75E-14	Ccl2,Ccl12,Ctsb,Cybb,Cyba,Trpv2,Tnfaip3,Ripk3,Tnf,Irf7,Il6
	mmu0514	Leishmaniasis	28	65	7.36E-13	Itgb2,Tgfb1,Cybb,Ncf1,Cyba,Nos2,C3,Tnf,Il1b,Il1a,Tlr2,Fcg
	mmu04[illegible]	Chemokine signaling pathway	44	179	8.11E-13	Ccl2,Ccl12,Ccl3,Hck,Vav1,Ccl8,Ncf1,Cxcl16,Ccl6,Ccl4,Ccl
	mmu0515	Staphylococcus aureus infection	25	50	1.05E-12	Itgb2,C3,Cfb,Fcgr1,C1qc,H2-DMa,C1qb,H2-Ab1,H2-Aa,C3a
	mmu0464	Hematopoietic cell lineage	29	90	7.1E-11	Il7r,Cd33,Cd44,Cd22,Tnf,Csf1r,Il6,Il1r2,Itga6,Il1b,Il1a,Il6ra,I
	mmu0516	Kaposi's sarcoma-associated herpesvirus infection	42	203	3.05E-10	Pdgfb,Hck,Bid,H2-M2,Pik3r5,Cdkn1a,C3,H2-K1,Irf7,Il6,Ccr
14	mmu0406	NF-kappa B signaling pathway	28	93	5.41E-10	Lbp,Cd40,Ccl4,Tnfaip3,Plau,Tnf,Il1b,Vcam1,Tnfrsf1a,Btk,P
15	mmu0514	Malaria	20	45	1.41E-09	Ccl2,Ccl12,Itgb2,Tgfb1,Cd40,Sdc1,Tnf,Il6,Sele,Il1b,Vcam1
16	mmu0513	Pertussis	24	74	3.49E-09	Itgb2,Nos2,C3,Tnf,Il6,Casp1,Il1b,Il1a,Cxcl5,Myd88,C1qc,Ir
17	mmu0415	PI3K-Akt signaling pathway	54	349	5.29E-09	Pdgfb,Col1a1,Lamb1,Il7r,Epha2,Ywhah,Grb2,Pik3r5,Osmr,
18	mmu0462	Toll-like receptor signaling pathway	27	98	5.29E-09	Ccl3,Irf5,Ctsk,Lbp,Cd40,Ccl4,Tnf,Irf7,Il6,Casp8,Il1b,Tlr2,Pil
19	mmu0465	Natural killer cell mediated cytotoxicity	28	111	1.22E-08	Itgb2,Bid,Cd244,Vav1,Grb2,H2-K1,Tnf,Nras,Tyrobp,Pik3cb,
20	mmu0516	Influenza A	34	165	1.85E-08	Ccl2,Ccl12,Ciita,Tnf,Il33,Irf7,Il6,Casp1,Il1b,Il1a,Tnfrsf1a,Pil
21	mmu0513	Salmonella infection	23	78	2.91E-08	Ccl3,Lbp,Nos2,Ccl4,Il6,Casp1,Il1b,Il1a,Cxcl1,Was,Myd88,T
22	mmu0421	Apoptosis	30	135	3.45E-08	Ern1,Bid,Ctsb,Ctsk,Ctss,Ctsz,Ctsl,Tnf,Pmaip1,Casp8,Nras
23	mmu0481	Regulation of actin cytoskeleton	38	208	3.45E-08	Itgb2,Pdgfb,Vav1,Pip4k2a,Myh9,Gna13,Diap1,Baiap2,Itga6
24	mmu0461	Complement and coagulation cascades	24	88	4.18E-08	Itgb2,Plaur,Plau,Clu,C3,Cfb,Procr,Itgax,F7,C1qc,C1qb,C3a
25	mmu0466	B cell receptor signaling pathway	21	69	7.56E-08	Vav1,Cd22,Grb2,Nfkbie,Ifitm1,Nras,Cd72,Btk,Pik3cb,Lyn,P
26	mmu0466	TNF signaling pathway	26	108	8.03E-08	Ccl2,Ccl12,Tnfaip3,Edn1,Ripk3,Tnf,Il6,Casp8,Sele,Il1b,Vca
27	mmu0493	AGE-RAGE signaling pathway in diabetic complicat	25	100	8.03E-08	Ccl2,Ccl12,Col1a1,Tgfb1,Cybb,Edn1,Tnf,Diap1,Il6,Sele,Il1b

Results inspection – Inspecting each block of the clustermap is easy. reString organizes all functional enrichment files it retrieves from STRING, for each comparison, into a folder with the same name as the input file. In this example, the term "Immune System" of Reactome Pathways is investigated in the comparison "treatment_t1_red_VS_treatment_t1_blue_FC" (**A**). The corresponding files for RCTM data are found in the corresponding folder (**B**), and once opened, it is easy to find if the term was found with the up- or down-regulated gene lists (**C**) and retrieve the genes of interest (arrows).

Supplementary Figure S15

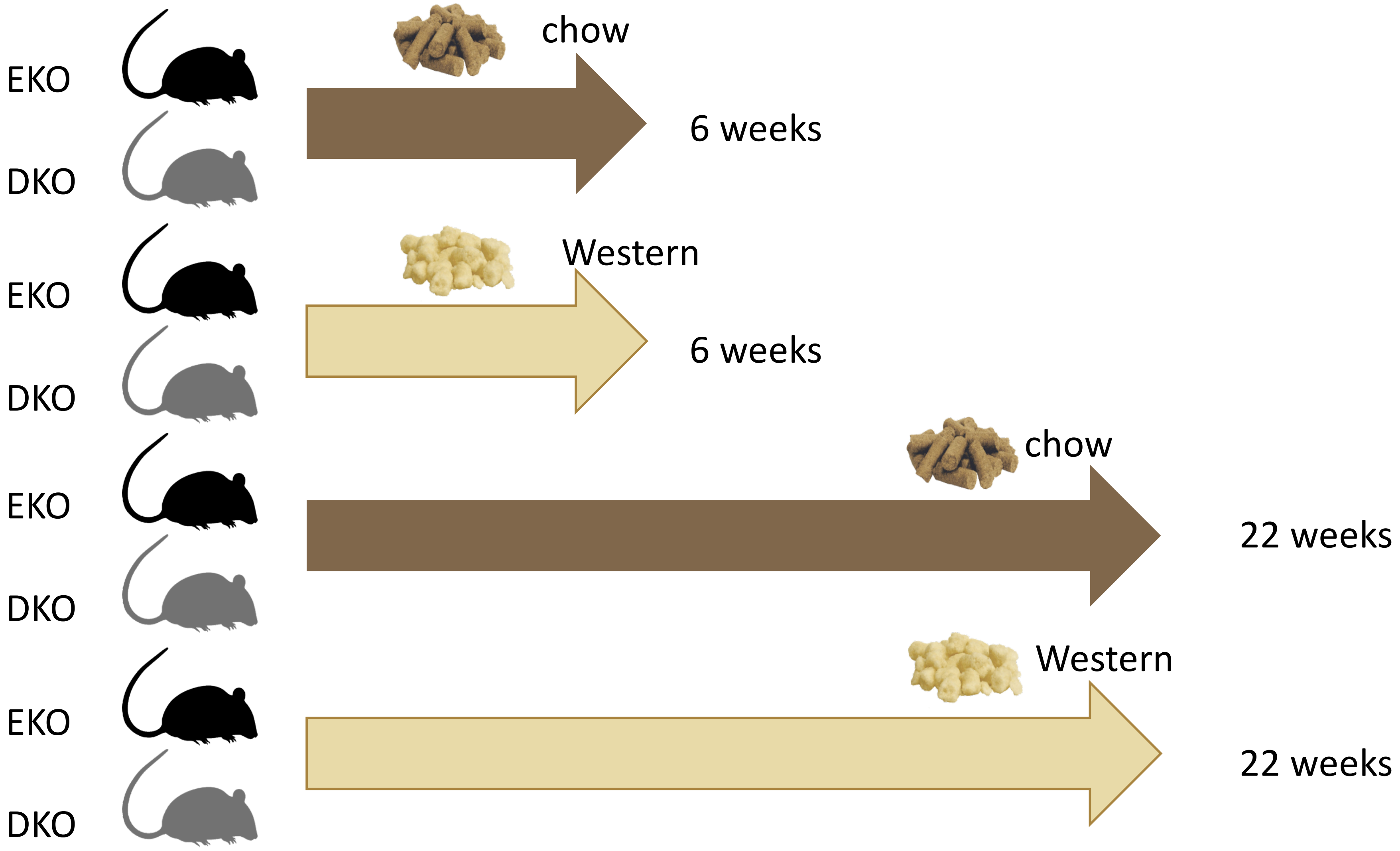

Experimental design. Eight weeks old male mice were randomly divided, genotype-wise, into 8 groups and fed either a normal laboratory diet (NLD, 4RF21, Mucedola, Italy) or a Western-type diet (WD, TD.88137, Envigo, Italy) for 6 or 22 weeks.

Supplementary Figure S16

A

Dunnett's T3 multiple comparisons test	Summary	Adjusted P Value	t	DF
DKO 6 NLD vs. EKO 6 NLD	ns	>0.9999999999	0	10,00
DKO 6 NLD vs. EKO 22 WD	**	0.006507451	3164	6000
EKO 6 NLD vs. EKO 22 WD	**	0.006507451	2105	5000
EKO 22 NLD vs. EKO 22 WD	**	0.006716819	4198	5000
DKO 22 NLD vs. EKO 22 WD	**	0.007111354	3838	5000
DKO 6 WD vs. EKO 22 WD	*	0.010198495	4426	5000
EKO 6 WD vs. EKO 22 WD	*	0.025153616	8092	5000
DKO 6 NLD vs. DKO 22 WD	ns	0.08407745	3164	6000
EKO 6 NLD vs. DKO 22 WD	ns	0.08407745	2105	5000
DKO 6 NLD vs. DKO 6 WD	ns	0.102325199	4198	5000
EKO 6 NLD vs. DKO 6 WD	ns	0.102325199	3838	5000
EKO 22 NLD vs. DKO 22 WD	ns	0.132090987	4426	5000
DKO 6 NLD vs. EKO 6 WD	ns	0.140726879	8092	5000
EKO 6 NLD vs. EKO 6 WD	ns	0.140726879	0,4284	10,29
DKO 22 NLD vs. DKO 22 WD	ns	0.148360436	1015	10,57
DKO 6 WD vs. DKO 22 WD	ns	0.216937032	2236	6912
DKO 6 NLD vs. DKO 22 NLD	ns	0.234282101	3600	5535
EKO 6 NLD vs. DKO 22 NLD	ns	0.234282101	6948	5774
EKO 22 NLD vs. EKO 6 WD	ns	0.471763384	1342	9962
DKO 22 NLD vs. EKO 6 WD	ns	0.576007491	2425	7545
EKO 6 WD vs. DKO 22 WD	ns	0.625856404	3712	5748
DKO 6 NLD vs. EKO 22 NLD	ns	0.650687267	7024	6077
EKO 6 NLD vs. EKO 22 NLD	ns	0.650687267	1591	7285
DKO 22 WD vs. EKO 22 WD	ns	0.834527223	3238	5662
DKO 6 WD vs. EKO 6 WD	ns	0.896062292	6484	5955
EKO 22 NLD vs. DKO 6 WD	ns	0.975162821	2138	7555
DKO 22 NLD vs. DKO 6 WD	ns	0.998894774	4795	8438
DKO 22 NLD vs. EKO 22 NLD	ns	1	1766	9674

B

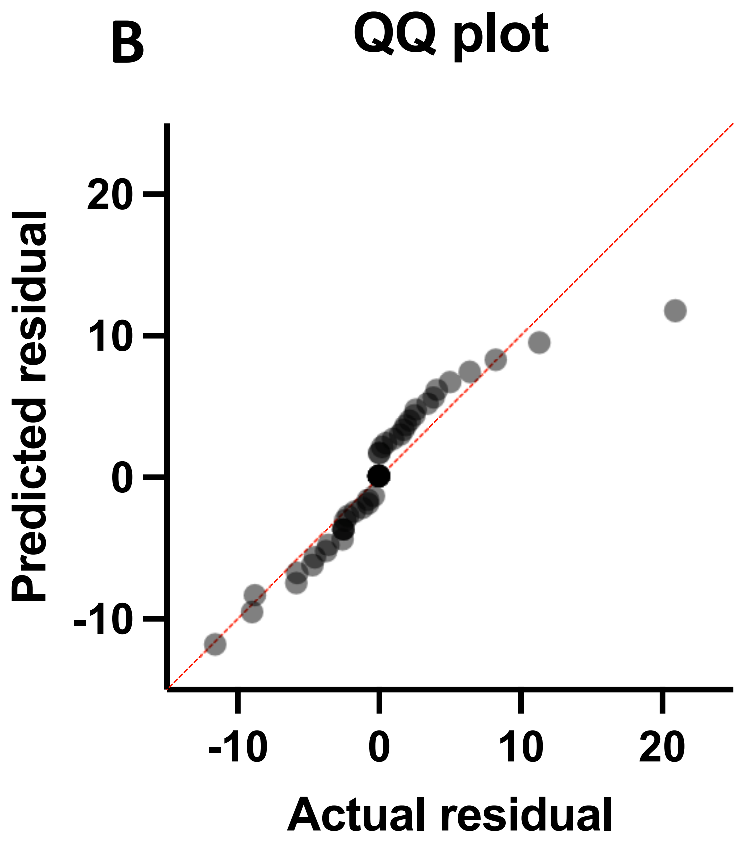

Plaque extent in EKO and DKO mice: detailed statistics. Detailed results of the *post-hoc* testing for each group, including the ones whose differences are not statistically significant, are shown in **A**. In **B**, a quantile-quantile (QQ) plot of all residuals is shown. Plaque extent distributes normally, thus a parametric multi-comparison test (Welch's ANOVA) has been chosen to test for statistical significance among groups.

Supplementary Figure S17

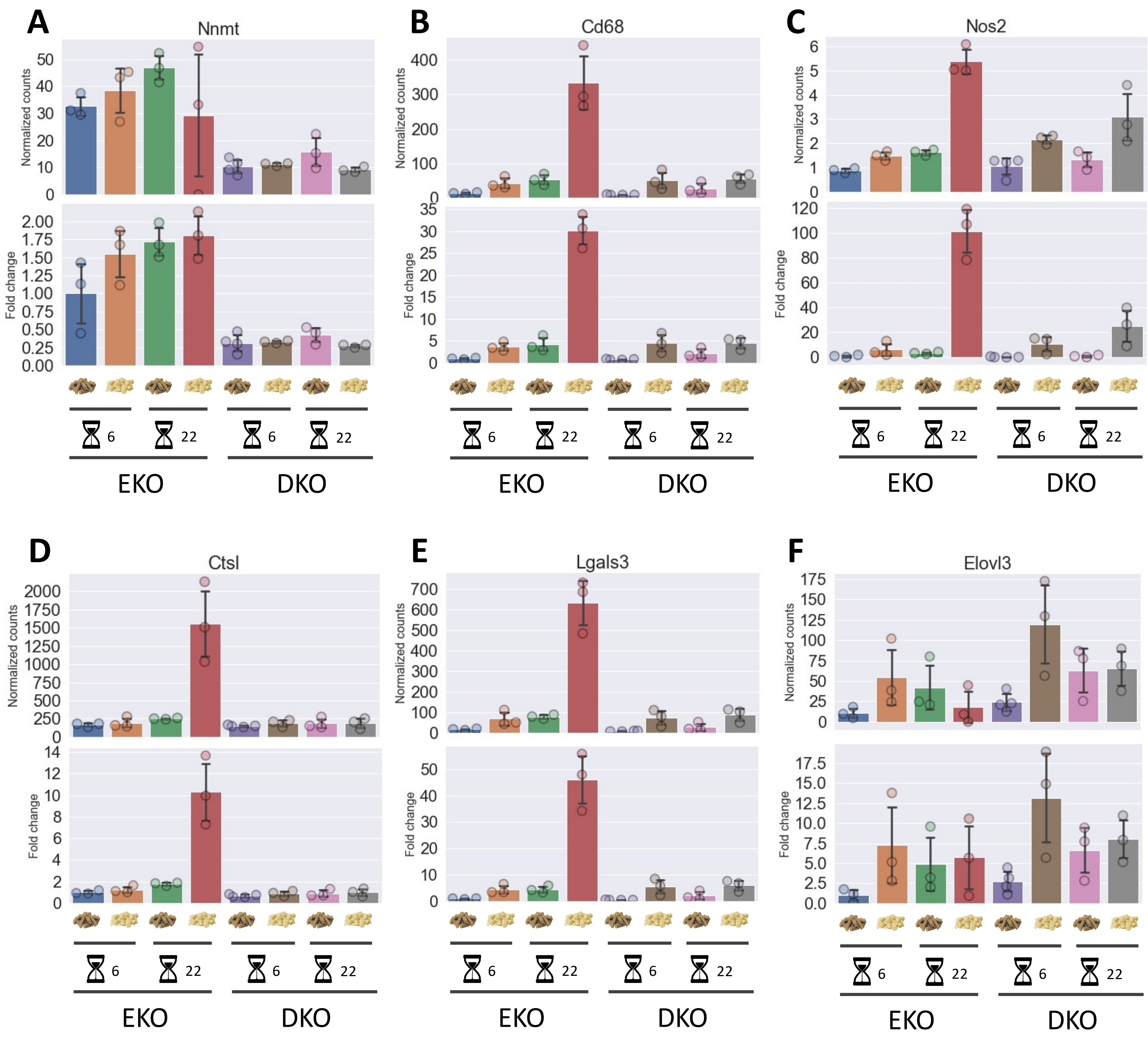

Six genes have been chosen for independent validation by qPCR (**A**-**F**). As a proof of principle, all samples were retro-transcribed and the resulting relative expression (EKO, NLD and 6 weeks was set as the relative reference group, blue) are compared head-to-head with corresponding normalized counts from RNAseq (bottom, qPCR data, top, RNAseq data). In every case (150 individual values), the correspondence between qPCR and normalized counts for each gene (converted to fold change vs the same reference group, then standard scaled) is remarkable (**G**)

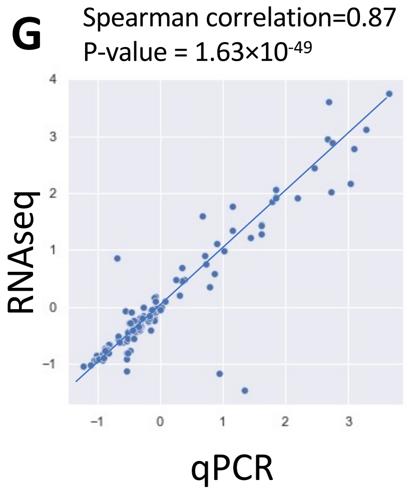

Supplementary Table S1

Target	Primer A (5' → 3')	Primer B (5' → 3')	Size (bp)	Temp (°C)	Type	Accession(s)
Srp14	GGAGGCTTCTGCTGACGGCG	GGGCTCGAGGCCCTCCACA	193 (cDNA)<br>677 (gDNA)	58.5	endpoint PCR	NM_009273.4
Tbp	ACCGTGAATCTTGGCTGTAAAC	GCAGCAAATCGCTTGGGATTA	86 bp	60	qPCR	NM_013684.3
Cd68	TGTTCAGCTCCAAGCCCAAA	GTACCGTCACAACCTCCCTG	123 bp	60	qPCR	NM_001291058.1
Ctsl	CAAATAAGAATAAATATTGGCTTGTCA	TGTAGCCTTCCATACCCCATT	66 bp	60	qPCR	NM_009984.4
Elovl3	GCCTCTCATCCTCTGGTCCT	TGCCATAAACTTCCACATCCT	76	60	qPCR	NM_007703.2,<br>NM_001374665.1
Lgals3	TGGACCACTGACGGTGCC	GGCAACATCATTCCCTCTCCT	136	60	qPCR	NM_010705.3,<br>NM_001145953.1
Nnmt	GAGCCTTTGACTGGTCCCC	GCACGCCTCAACTTCTCCTC	94	60	qPCR	NM_010924.3,<br>NM_001311062.1
Nos2	CGTGAAGAAAACCCCTTGTGCT	AGGGATTCTGGAACATTCTGTGCT	117	60	qPCR	NM_010927.4

For endpoint PCR, the conditions were as follows: 95 °C for 3 min, followed by 35 cycles of 30 s at 95 °C, 30 s at 58.5 °C, 45 s at 72 °C for 45 s, followed by a final amplification step of 5 min at 72 °C. For qPCR, the conditions were as follows: 95 °C for 1 min, followed by 40 cycles of 10 s at 95 °C, 30 s at 60 °C, followed by a final melting curve performed from 65.0 °C to 95.0 °C with 0.5 °C increments.

Supplementary Materials and Methods

This document mirrors the online documentation currently available (September, 2021) for reString at the GitHub reString repo: https://github.com/Stemanz

Please refer to the online doc for the latest changes and updates.

restring

Easy to use functional enrichment terms retriever and aggregator, designed for the wet biology researcher

Overview

`restring` works on user-supplied differentially expressed (DE) genes list, and **automatically pulls and aggregates functional enrichment data** from STRING.
It returns, in table-friendly format, aggregated results from **analyses of multiple comparisons**.

Results can readily be visualized via **highly customizable heat/clustermaps** to produce beautiful publication-grade pics. Plus, it's got a GUI!

What KEGG pathway was found in which comparisons? What pvalues? What DE genes annotated in that pathway were shared in those comparisons? How can I simultaneously show results for all my experimental groups, for all terms, all at once? This can all be managed by `restring`.

Table of contents

- Use case
- Installation
  - Installation in depth
  - Installation troubleshooting
- Procedure
  - 1 | Prepping the files
  - 2 | Set input files and choosing the output path
  - 3 | Running the analysis (with default settings)
    - Results tables
    - Summary tables
  - 4 | Visualizing the results
    - Clustermap/Heatmap
    - Clustermap customization options
    - Bubble plot

        - Bubble plot options
    - 5 | Configuring the analysis
        - Species
        - DE genes settings
        - Set background
        - Clear background
        - Choosing a specific STRING version
- Getting help
    - Investigating an issue
    - Reporting a bug
    - Requesting a new feature
- Known bugs
- **restring as a Python module**
    - 1 | Download required files from STRING
    - 2 | Aggregating the results
    - 3 | Visualizing the results with `draw_clustermap()`
    - 4 | Polishing up: making the clutermap pop

Use case

Modern high-throughput -omic approaches generate huge lists of differentially expressed (DE) genes/proteins, which can in turn be used for functional enrichment studies. Manualy reviewing a large number of such analyses is time consuming, especially for experimental designs with more than a few groups. Let's consider this experimental setup:

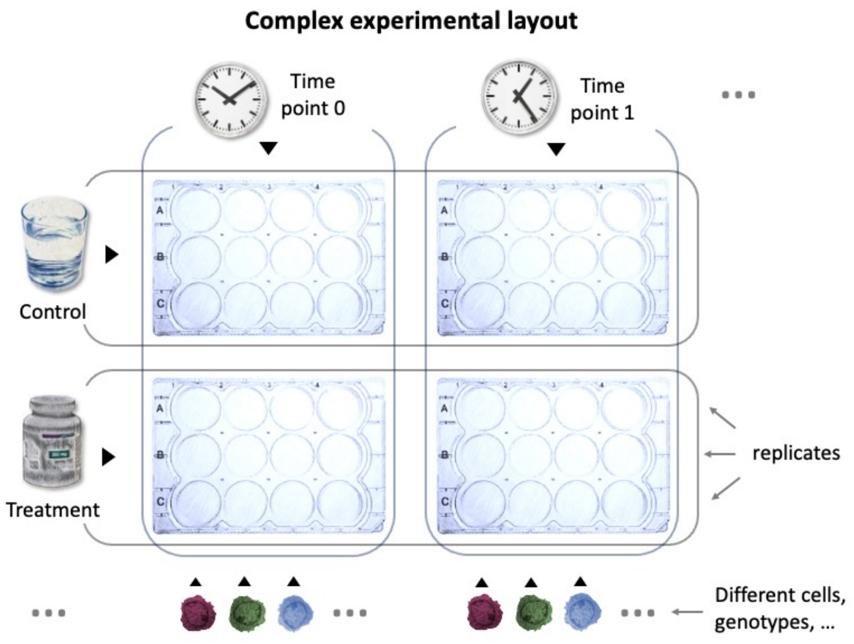

This represents a fairly common experimental design, but manually inspecting functional enrichment results for such all possible combinations would require substantial effort. Let's take a look at how we can tackle this issue with `restring`.
Our sample experimental setup has **two treatments**, given at **two time points** to **three different sample types**. Let's assume those samples are cells of different genotypes, and we'd like to mainly investigate genotype comparisons. After quantifying gene expression by RNAseq, we have DE genes for every comparison. As in many experimental pipelines, each list of DE genes is investigated with functional enrichment tools, such as [String](). But every comparison generates one or more tables. `restring` makes it easy to generate summary reports from all of them, automatically.

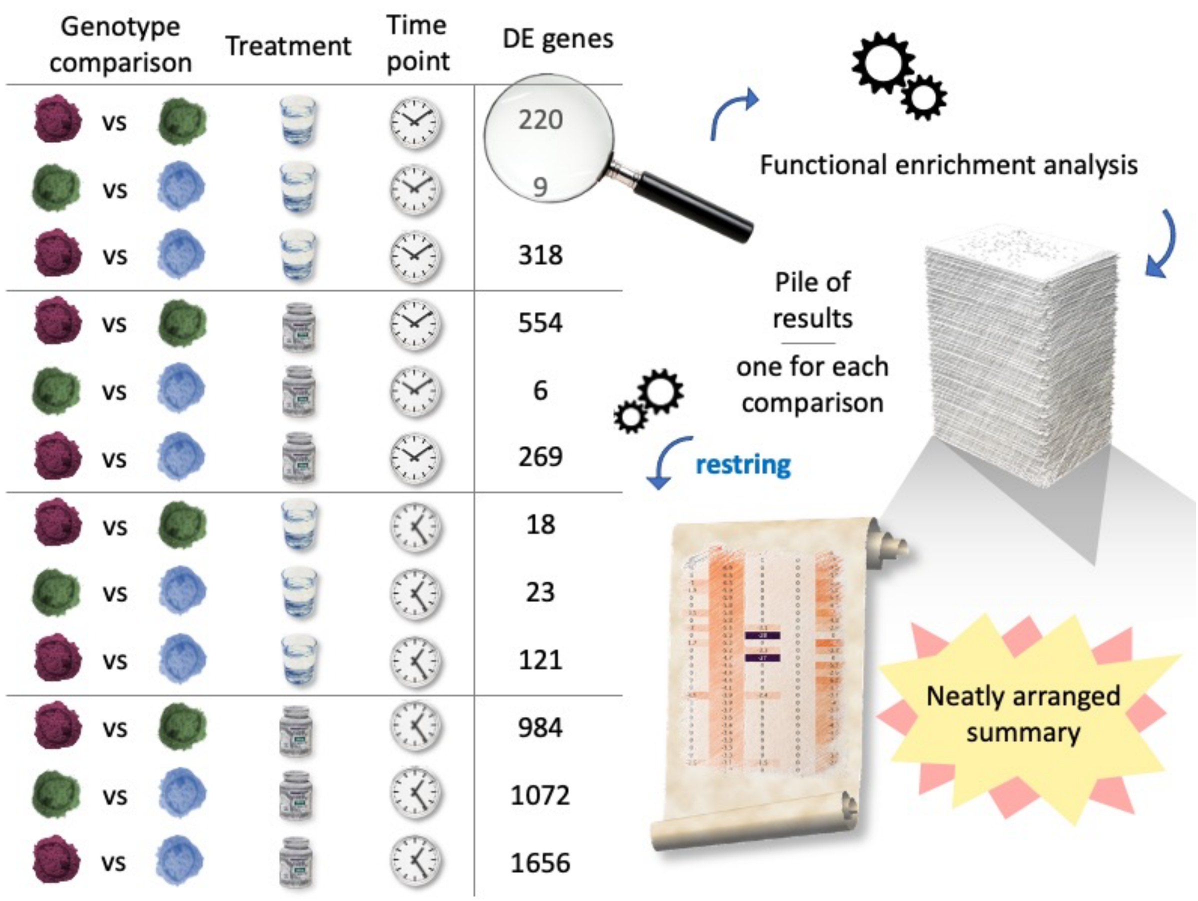

Installation

`reString` is a Python application, and requires Python to run. Please refer to Python's official page for installation: [https://www.python.org/]().
Once you have Python up and running, installing `reString` is as simple as opening up a terminal window and typing:
`pip install restring`
Here's what the installation process looks like in the Mac:

```
(restest) cln-169-032-dhcp:~ manz$ pip install restring
Collecting restring
```

```
  Downloading
https://files.pythonhosted.org/packages/58/4c/03f7f06a15619bd8b53ada077a2e
4f9d7cd187d9e9857ab57b3239963744/restring-0.1.16.tar.gz (1.2MB)
    100% |████████████████████████████████| 1.2MB 437kB/s
Requirement already satisfied: matplotlib in
/Applications/Anaconda3/anaconda/lib/python3.4/site-packages (from
restring)
Requirement already satisfied: seaborn in
/Applications/Anaconda3/anaconda/lib/python3.4/site-packages (from
restring)
Requirement already satisfied: pandas in
/Applications/Anaconda3/anaconda/lib/python3.4/site-packages (from
restring)
Requirement already satisfied: requests in
/Applications/Anaconda3/anaconda/lib/python3.4/site-packages (from
restring)
Requirement already satisfied: numpy>=1.6 in
/Applications/Anaconda3/anaconda/lib/python3.4/site-packages (from
matplotlib->restring)
Requirement already satisfied: python-dateutil in
/Applications/Anaconda3/anaconda/lib/python3.4/site-packages (from
matplotlib->restring)
Requirement already satisfied: pytz in
/Applications/Anaconda3/anaconda/lib/python3.4/site-packages (from
matplotlib->restring)
Requirement already satisfied: cycler in
/Applications/Anaconda3/anaconda/lib/python3.4/site-packages (from
matplotlib->restring)
Requirement already satisfied: pyparsing!=2.0.4,>=1.5.6 in
/Applications/Anaconda3/anaconda/lib/python3.4/site-packages (from
matplotlib->restring)
Requirement already satisfied: six>=1.5 in
/Applications/Anaconda3/anaconda/lib/python3.4/site-packages (from python-
dateutil->matplotlib->restring)
Building wheels for collected packages: restring
  Running setup.py bdist_wheel for restring ... done
  Stored in directory:
/Users/manz/Library/Caches/pip/wheels/25/67/13/73711665f987ae891784bef729f
350429599d2e3cda015a37a
Successfully built restring
Installing collected packages: restring
Successfully installed restring-0.1.16
(restest) cln-169-032-dhcp:~ manz$
```

To **run** restring, simply open a terminal and type:

```
restring-gui
```

This will launch `reString` in its GUI form. On Windows systems, the first time the antivirus might want to check `restring-gui.exe`, but the application should launch without issues once it realizes there are no threats.

This is what it looks like in MacOS:

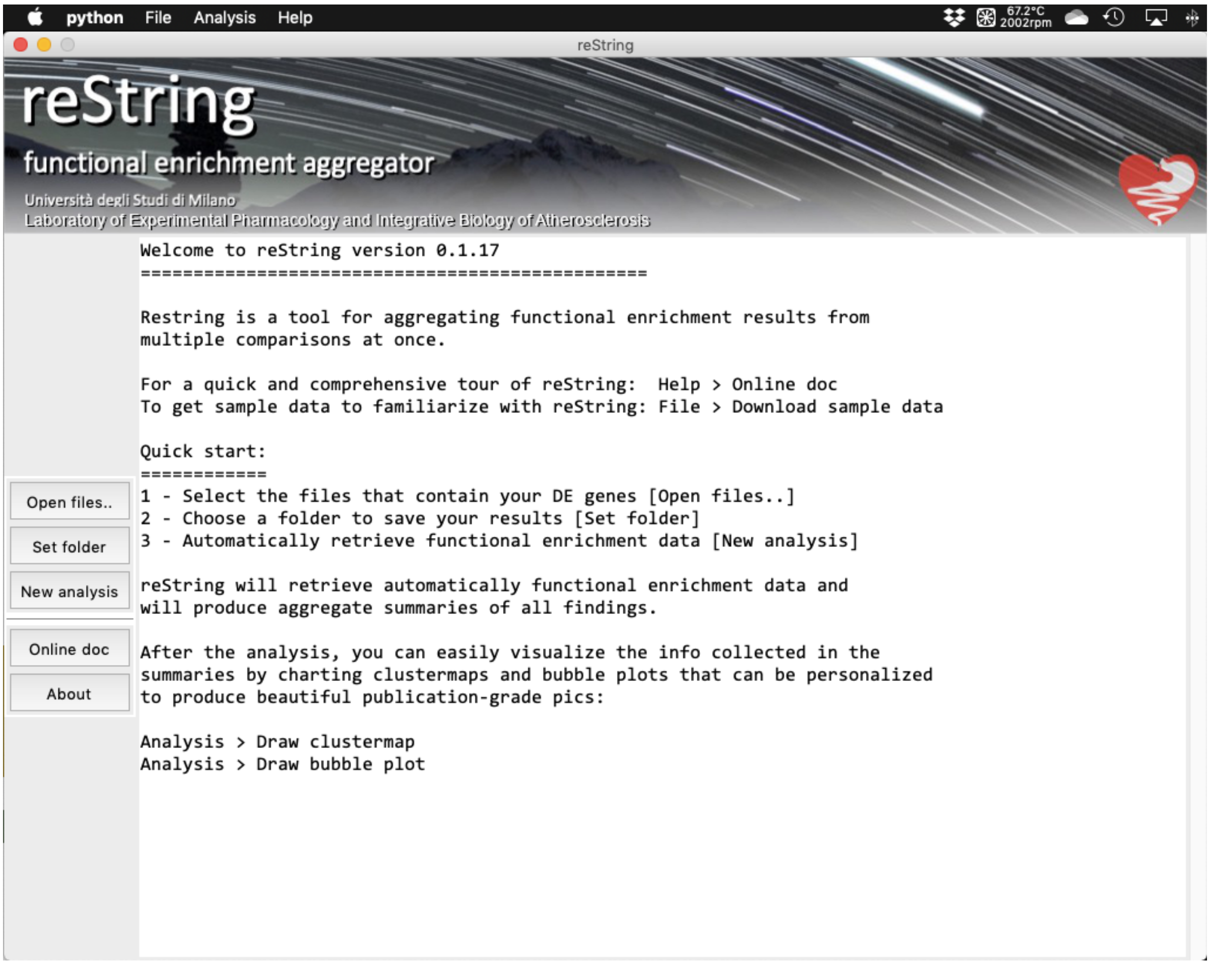

Installation in depth

Here are step-by-step instructions on how to install `reString` on specific platforms in the form of YouTube videos.

[Windows 10:]https://www.youtube.com/watch?v=agIYg93ticI

[Mac OS:]https://www.youtube.com/watch?v=7zRQrWpRi1E

[Ubuntu GNU/linux:]https://www.youtube.com/watch?v=Lejia7_Zcp0

[Raspberry Pi OS:]https://www.youtube.com/watch?v=RybteNoaWOI

In each video description, the commands that should be inputted in the terminal to perfect the installation process are handily summarized. This covers both checking/installing Python, eventual missing dependencies and `restring` itself.

Installation troubleshooting

If you experience hiccups during the installation, maybe we got you covered:

- If you get `SyntaxError` after trying to run `restring`:

make sure you are using Python 3.x and **not** Python 2.x. Python 2.x is *obsolete* and *discontinued*. Many systems still support both, in this case you use `python` and `pip` for Python 2.x and `python3` and `pip3` or Python 3.x. In this case, use `pip3` to install restring.

- If you get `SyntaxError` and you are **sure** you're running Python 3.x:

then you're running a version prior to 3.6. Update it.

- If you get errors launching reString by typing `restring-gui`:

To the exception of MacOS, we noticed that the installation script is not placed in the `Path`/`PATH` environment variable *(that is: even if the script is in your computer, your computer doesn't know where to pull it from when you type it)*.

If this happens, you have two alternatives:

alternative a) start `restring` by typing

```
python -c "import restring; restring.restring_gui()"
```

or

```
python3 -c "import restring; restring.restring_gui()"
```

Use the first command if `python` is Python 3.x in your system, use `python3` if in your system the version 2.x is called instead. These commands are guaranteed to work from within any folder the terminal is in;

alternative b) permanently teach your system where the launch script lies. You will know the location from the installation log (refer to the YouTube videos). In GNU/linux systems, it's far easier to google for something like "how to permanently add a folder to PATH in YOUR_DISTRO_HERE". In Windows, follow the instructions of the YouTube installation guide. When done, you will be able to launch restring by just typing:

```
restring-gui
```

- If you get weird errors:

Get in touch with us: report a bug.

Procedure

`restring` can be used via its graphical user interface (recommended). A full protocol, with sample data and examples, is detailed below.
Alternatively, it can be imported as a Python module. This hands-on procedure is detailed at the end of this document.

`restring` GUI

1 | Prepping the files

All `restring` requires is a gene list of choice per experimental condition. This gene list needs to be in tabular form, arranged like this [sample data](). This is very easily managed with any spreadsheet editor, such as Microsoft's Excel or Libre Office's Calc.

2 | Set input files and output path

In the menu, choose `File > Open...`, or hit the `Open files..` button.
Tip: put all input files you want to process together in one or more analyses in the same folder. Input files can be individually selected from any one folder, but each time input files are added, the input files list is reset.

Then, choose an existing directory where all putput files will be placed: choose `File > Set output folder` or hit `Set folder` button *(Choose a different output folder each time the analysis parameters are varied, see section 5)*.

3 | Running the analysis with default settings

In the menu, choose `Analysis > New analysis`, or hit the `New analysis` button.
`restring` will look for genes in the files you have specified, interrogate STRING to get functional enrichment data back *(these tables, looking exactly the same to the ones you would manually retrieve, will be saved into subfolders of the output folder)*, then write aggregated results and summaries.
These are found in the specified output directory, and take the form of **results**- or **summary**-type tables, in `.tsv` *(tab separated values)* format, that can be opened out-of-the-box by Excel or Calc. Let's take a look at the anatomy of these tables.

Results tables

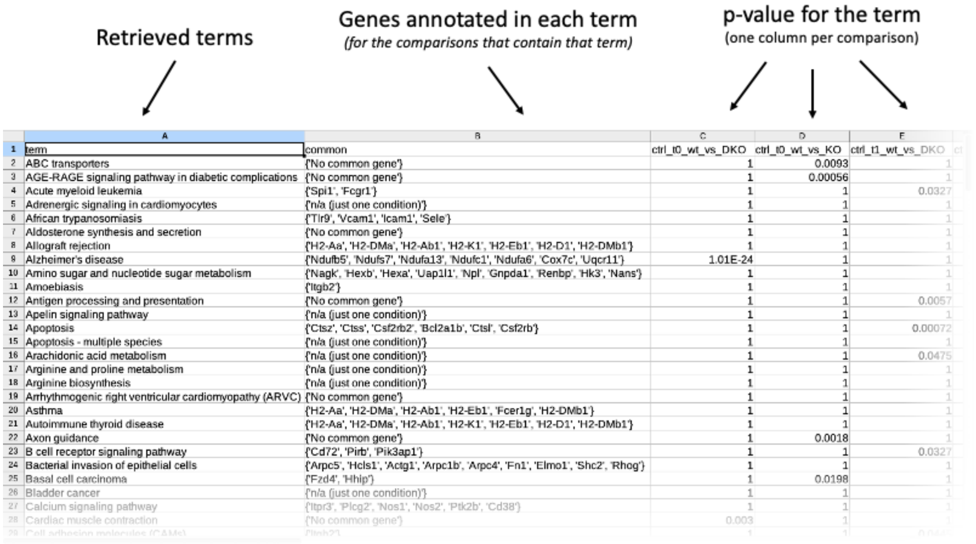

	A	B	C	D	E
1	term	common	ctrl_t0_wt_vs_DKO	ctrl_t0_wt_vs_KO	ctrl_t1_wt_vs_DKO
2	ABC transporters	{'No common gene'}	1	0.0093	1
3	AGE-RAGE signaling pathway in diabetic complications	{'No common gene'}	1	0.00056	1
4	Acute myeloid leukemia	{'Spi1', 'Fcgr1'}	1	1	0.0327
5	Adrenergic signaling in cardiomyocytes	{'n/a (just one condition)'}	1	1	1
6	African trypanosomiasis	{'Tlr9', 'Vcam1', 'Icam1', 'Sele'}	1	1	1
7	Aldosterone synthesis and secretion	{'No common gene'}	1	1	1
8	Allograft rejection	{'H2-Aa', 'H2-DMa', 'H2-Ab1', 'H2-K1', 'H2-Eb1', 'H2-D1', 'H2-DMb1'}	1	1	1
9	Alzheimer's disease	{'Ndufb5', 'Ndufs7', 'Ndufa13', 'Ndufc1', 'Ndufa6', 'Cox7c', 'Uqcr11'}	1.01E-24	1	1
10	Amino sugar and nucleotide sugar metabolism	{'Nagk', 'Hexb', 'Hexa', 'Uap1l1', 'Npl', 'Gnpda1', 'Renbp', 'Hk3', 'Nans'}	1	1	1
11	Amoebiasis	{'Itgb2'}	1	1	1
12	Antigen processing and presentation	{'No common gene'}	1	1	0.0057
13	Apelin signaling pathway	{'n/a (just one condition)'}	1	1	1
14	Apoptosis	{'Ctsz', 'Ctss', 'Csf2rb2', 'Bcl2a1b', 'Ctsl', 'Csf2rb'}	1	1	0.00072
15	Apoptosis - multiple species	{'n/a (just one condition)'}	1	1	1
16	Arachidonic acid metabolism	{'n/a (just one condition)'}	1	1	0.0475
17	Arginine and proline metabolism	{'n/a (just one condition)'}	1	1	1
18	Arginine biosynthesis	{'n/a (just one condition)'}	1	1	1
19	Arrhythmogenic right ventricular cardiomyopathy (ARVC)	{'No common gene'}	1	1	1
20	Asthma	{'H2-Aa', 'H2-DMa', 'H2-Ab1', 'H2-Eb1', 'Fcer1g', 'H2-DMb1'}	1	1	1
21	Autoimmune thyroid disease	{'H2-Aa', 'H2-DMa', 'H2-Ab1', 'H2-K1', 'H2-Eb1', 'H2-D1', 'H2-DMb1'}	1	1	1
22	Axon guidance	{'No common gene'}	1	0.0018	1
23	B cell receptor signaling pathway	{'Cd72', 'Pirb', 'Pik3ap1'}	1	1	0.0327
24	Bacterial invasion of epithelial cells	{'Arpc5', 'Hcls1', 'Actg1', 'Arpc1b', 'Arpc4', 'Fn1', 'Elmo1', 'Shc2', 'Rhog'}	1	1	1
25	Basal cell carcinoma	{'Fzd4', 'Hhip'}	1	0.0198	1
26	Bladder cancer	{'n/a (just one condition)'}	1	1	1
27	Calcium signaling pathway	{'Itpr3', 'Plcg2', 'Nos1', 'Nos2', 'Ptk2b', 'Cd38'}	1	1	1
28	Cardiac muscle contraction	{'No common gene'}	0.003	1	1
29	[illegible]	[illegible]	1	1	[illegible]

The table contains all terms cumulatively retrieved from all comparisons *(each one of the inpt files containing the genes of interest between any two experimental conditions)*. For every term, common genes (if any) are listed. These common genes only include comparisons where the term actually shows up. If the term just appears in exactly one comparison, this is explicitly stated: `n/a (just one condition)`. P-values are the ones retrieved from the STRING tables *(the lower, the better)*. Missing p-values are represented with 1 *(that is, in that specific comparison the term is 100% likely not enriched)*.

Summary tables

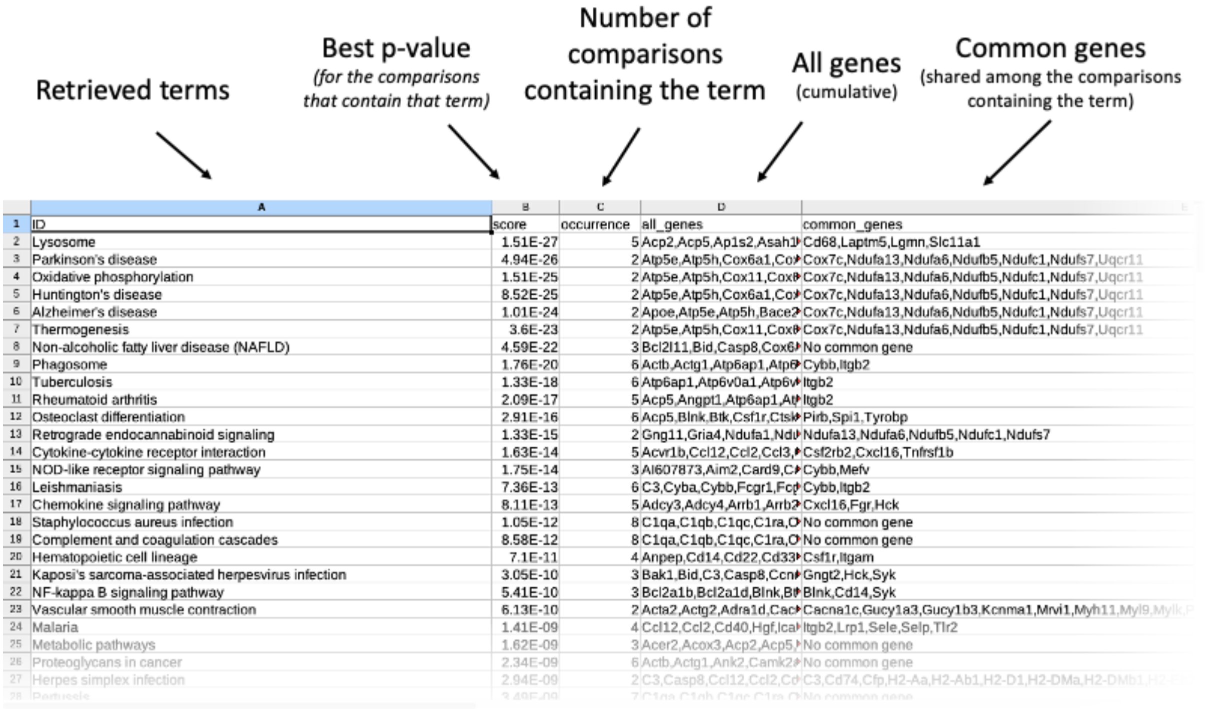

	A	B	C	D	
1	ID	score	occurrence	all_genes	common_genes
2	Lysosome	1.51E-27	5	Acp2,Acp5,Ap1s2,Asah1	Cd68,Laptm5,Lgmn,Slc11a1
3	Parkinson's disease	4.94E-26	2	Atp5e,Atp5h,Cox6a1,Co	Cox7c,Ndufa13,Ndufa6,Ndufb5,Ndufc1,Ndufs7,Uqcr11
4	Oxidative phosphorylation	1.51E-25	2	Atp5e,Atp5h,Cox11,Cox	Cox7c,Ndufa13,Ndufa6,Ndufb5,Ndufc1,Ndufs7,Uqcr11
5	Huntington's disease	8.52E-25	2	Atp5e,Atp5h,Cox6a1,Co	Cox7c,Ndufa13,Ndufa6,Ndufb5,Ndufc1,Ndufs7,Uqcr11
6	Alzheimer's disease	1.01E-24	2	Apoe,Atp5e,Atp5h,Bace2	Cox7c,Ndufa13,Ndufa6,Ndufb5,Ndufc1,Ndufs7,Uqcr11
7	Thermogenesis	3.6E-23	2	Atp5e,Atp5h,Cox11,Cox	Cox7c,Ndufa13,Ndufa6,Ndufb5,Ndufc1,Ndufs7,Uqcr11
8	Non-alcoholic fatty liver disease (NAFLD)	4.59E-22	3	Bcl2l11,Bid,Casp8,Cox6	No common gene
9	Phagosome	1.76E-20	6	Actb,Actg1,Atp6ap1,Atp6	Cybb,Itgb2
10	Tuberculosis	1.33E-18	6	Atp6ap1,Atp6v0a1,Atp6v	Itgb2
11	Rheumatoid arthritis	2.09E-17	5	Acp5,Angpt1,Atp6ap1,At	Itgb2
12	Osteoclast differentiation	2.91E-16	6	Acp5,Blnk,Btk,Csf1r,Ctsk	Pirb,Spi1,Tyrobp
13	Retrograde endocannabinoid signaling	1.33E-15	2	Gng11,Gria4,Ndufa1,Ndu	Ndufa13,Ndufa6,Ndufb5,Ndufc1,Ndufs7
14	Cytokine-cytokine receptor interaction	1.63E-14	5	Acvr1b,Ccl12,Ccl2,Ccl3,	Csf2rb2,Cxcl16,Tnfrsf1b
15	NOD-like receptor signaling pathway	1.75E-14	3	AI607873,Aim2,Card9,C	Cybb,Mefv
16	Leishmaniasis	7.36E-13	6	C3,Cyba,Cybb,Fcgr1,Fc	Cybb,Itgb2
17	Chemokine signaling pathway	8.11E-13	5	Adcy3,Adcy4,Arrb1,Arrb2	Cxcl16,Fgr,Hck
18	Staphylococcus aureus infection	1.05E-12	8	C1qa,C1qb,C1qc,C1ra,C	No common gene
19	Complement and coagulation cascades	8.58E-12	8	C1qa,C1qb,C1qc,C1ra,C	No common gene
20	Hematopoietic cell lineage	7.1E-11	4	Anpep,Cd14,Cd22,Cd33	Csf1r,Itgam
21	Kaposi's sarcoma-associated herpesvirus infection	3.05E-10	3	Bak1,Bid,C3,Casp8,Ccn	Gngt2,Hck,Syk
22	NF-kappa B signaling pathway	5.41E-10	3	Bcl2a1b,Bcl2a1d,Blnk,B	Blnk,Cd14,Syk
23	Vascular smooth muscle contraction	6.13E-10	2	Acta2,Actg2,Adra1d,Cac	Cacna1c,Gucy1a3,Gucy1b3,Kcnma1,Mrvi1,Myh11,Myl9,Mylk,P
24	Malaria	1.41E-09	4	Ccl12,Ccl2,Cd40,Hgf,Ica	Itgb2,Lrp1,Sele,Selp,Tlr2
25	Metabolic pathways	1.62E-09	3	Acer2,Acox3,Acp2,Acp5,	No common gene
26	Proteoglycans in cancer	2.34E-09	6	Actb,Actg1,Ank2,Camk2	No common gene
27	Herpes simplex infection	2.94E-09	2	C3,Casp8,Ccl12,Ccl2,Cd	C3,Cd74,Cfp,H2-Aa,H2-Ab1,H2-D1,H2-DMa,H2-DMb1,[illegible]
28	Pertussis	3.49E-09	7	C1qa,C1qb,C1qc,C1ra,C	No common gene

These can be useful to find the most interesting terms across all comparisons: better p-value, presence in most/selected comparisons), as well as finding the most recurring DE genes for each term.

4 | Visualizing the results

Clustermap

`restring` makes it easy to inspect the results by visualizing **results**-type tables as clustermaps. In the menu, choose `Analysis > Draw clustermap` to open the Draw clustermap window:

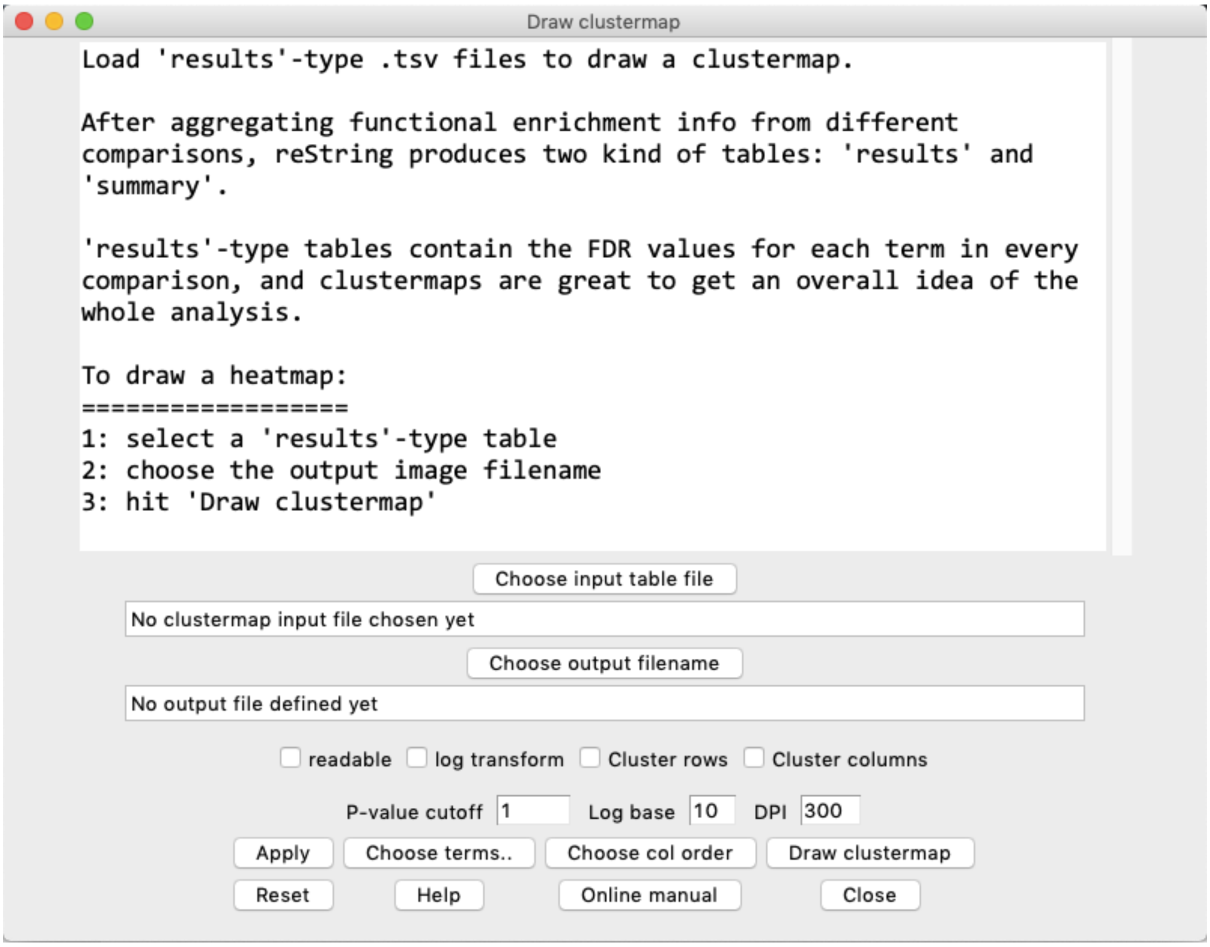

Clustermap Options

readable: if flagged, the output clustermap will be drawn as tall as required to fully display all the terms contained in it. Be warned that this might get very tall, depending on the number of terms.

log transform: if flagged, the p-values are minus log-transformed with the specified base: -log(number, base chosen). Hit `Apply` to apply.

cluster rows: if flagged, the rows are clustered (by distance) as per Scipy defaults. The column order is overridden.

cluster columns: if flagged, the columns are clustered (by distance) as per Scipy defaults.

P-value cutoff: For each term *(row)*, if all values are higher than the specified threshold, the term is not included in the clustermap. For log-transformed heatmaps, for each term *(row)*, if all values are lower than the specified threshold, the term is not included in the clustermap.

Insert a new value and hit `Apply` to see how many terms are retained/discarded by the new threshold.
Note that the default value of 1 will include all terms of a non-transformed table, as all terms are necessarily 1 or lower *(moreover, there should automatically be at least a term per row that was significant, at P=0.05, in the files retrieved from STRING, otherwise the term would not appear in the table in the first place)*.

To set a new threshold, for instance at P=0.001, one should input `0.001`, or 3 when log-transforming in base 10. Always hit `Apply`.
Log base: choose the base for the logarithm.

DPI: choose the output image resolution in DPI *(dot per inch)*. The higher, the larger the image.

Apply: Applies the current settings to the table, and shows how the settings impact on the table.

Choose terms..: This button opens a dialog to choose the terms. An example:

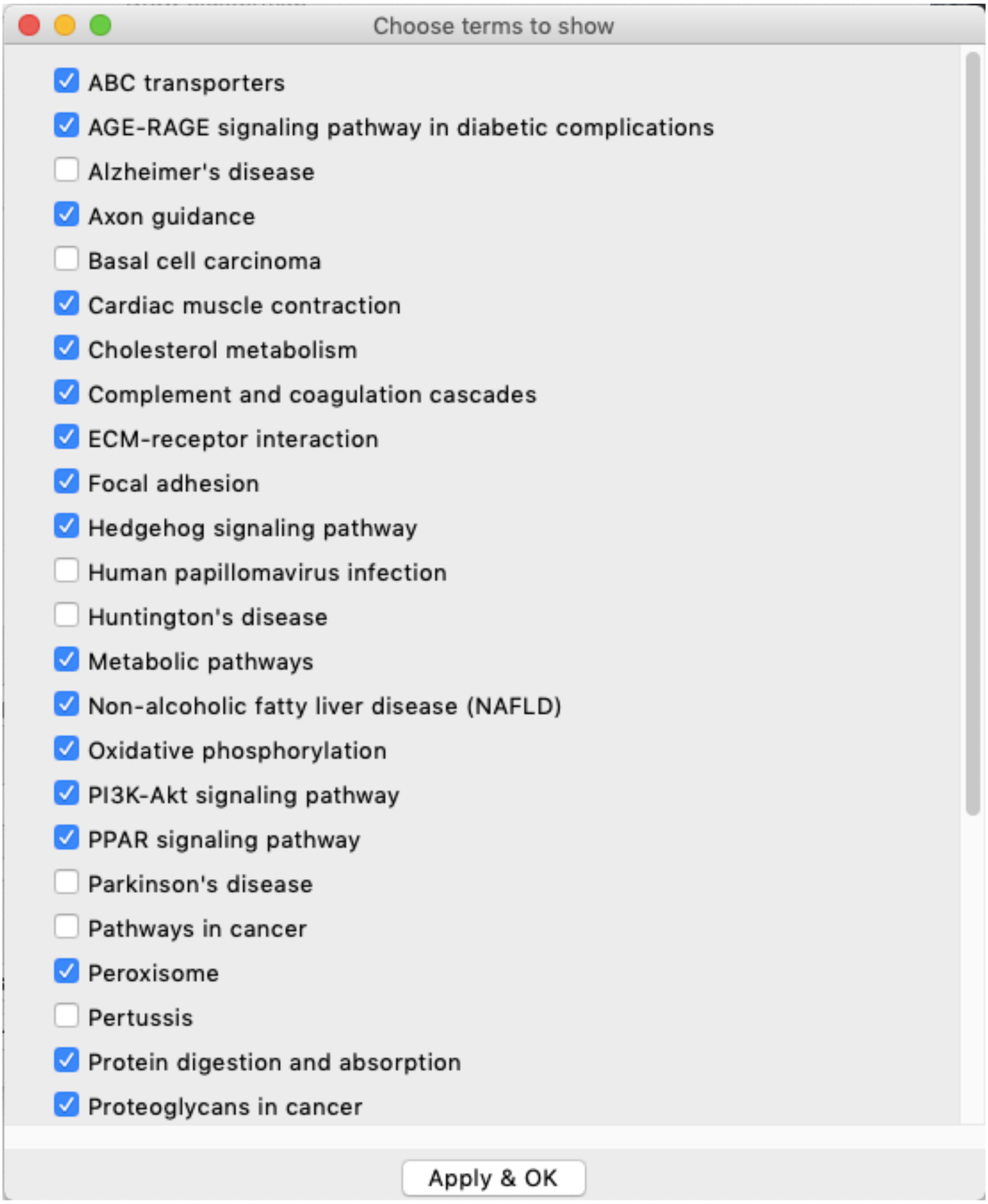

In this example, the results table contains terms that are irrelevant in the analysis being made. When loading a new table, all terms are automatically included, but the user chan choose to untick the terms that are unwanted. If a new **P-value cutoff** is applied, `restring` remembers the user choice even if some of the terms are now removed from the term list and are added back to the table at a later time.
Hit `Apply & OK` to apply the choice and close the window.

Choose col order: The user can reorder the column order by dragging the column names. Multiple adjacent columns can be selected and dragged together *(this is ineffective if* **Cluster rows** *is flagged)*.

Hit `OK` to apply and close the window.

Draw clustermap: Draws, saves and opens the clustermap.

Reset: Reloads the input table and clears term selection.

Help: Opens a dialog that briefly outlines the procedure.

Online Manual: Opens the default browser at the clustermap help section.

Close: Closes the window.

Bubble plot

`restring` makes it easy to inspect the results by visualizing **summary**-type tables as bubble plots.
In the menu, choose `Analysis > Draw bubble plot` to open the Draw bubble plot window:

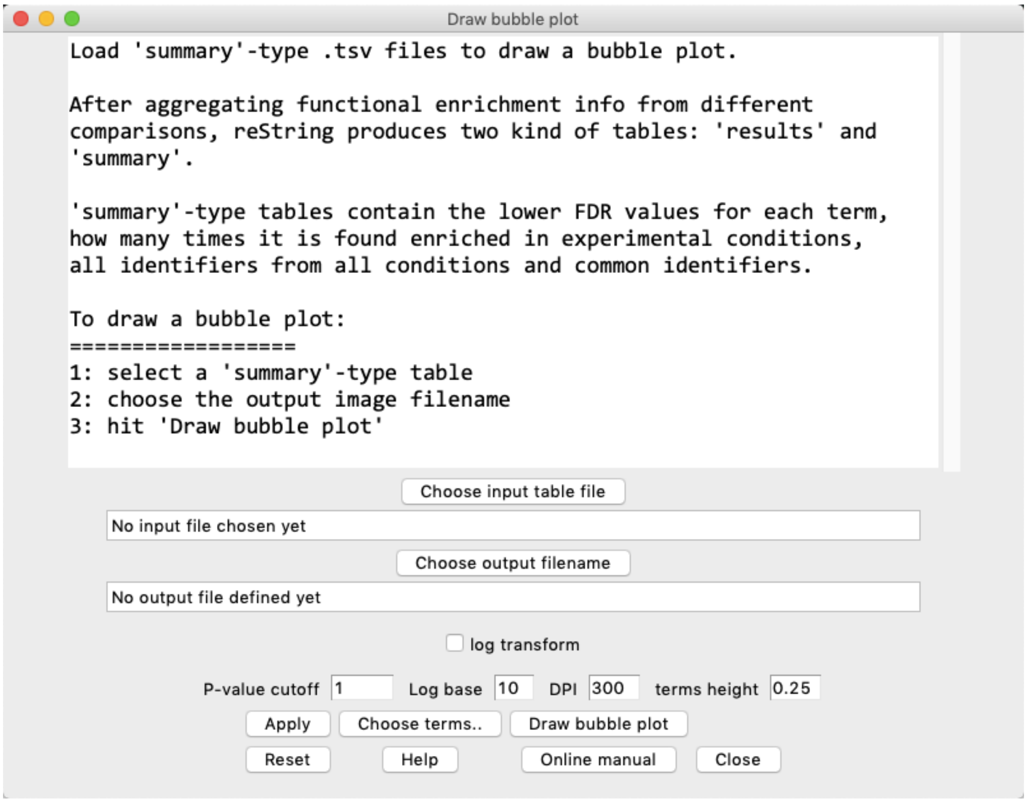

The bubble plot emphasizes the information gathered in summary-type tables, drawing, for each selected term, a bubble whose color and size reflect the FDR (color) and number of genes shared between all experimental conditions for each term (size). Here is an example:

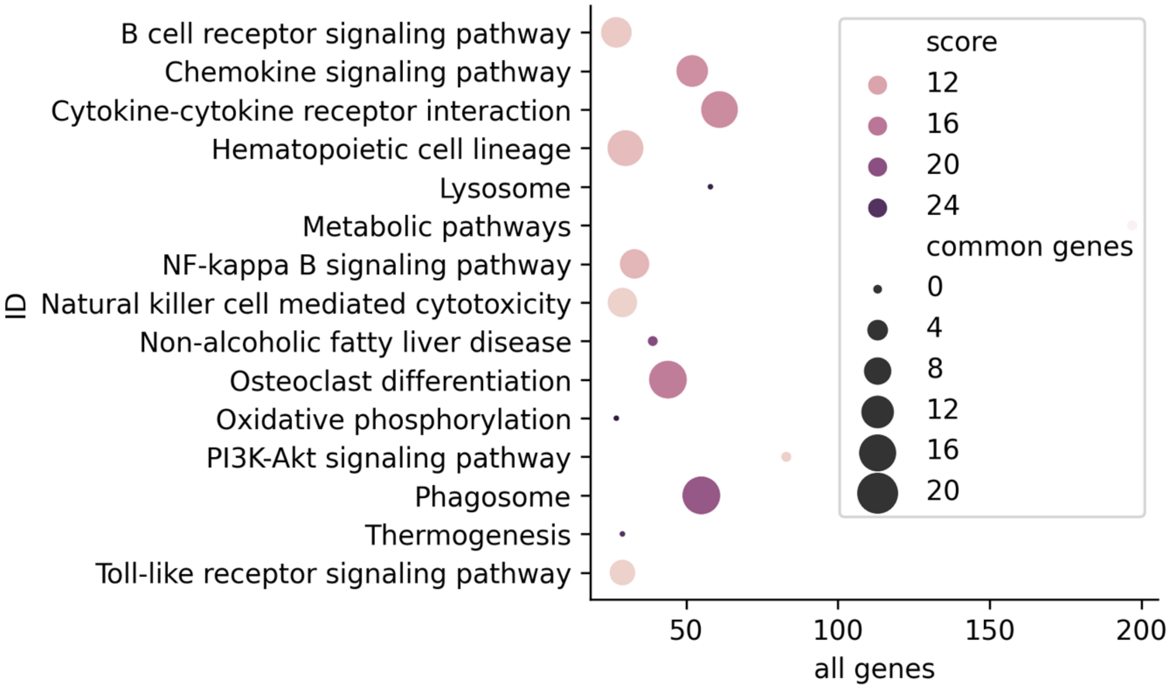

Bubble plot options

log transform: if flagged, the p-values are minus log-transformed with the specified base: -log(number, base chosen). Hit `Apply` to apply.

P-value cutoff: For each term *(row)*, if all values are higher than the specified threshold, the term is not included in the clustermap. For log-transformed heatmaps, for each term *(row)*, if all values are lower than the specified threshold, the term is not included in the clustermap.

Insert a new value and hit `Apply` to see how many terms are retained/discarded by the new threshold.
Note that the default value of 1 will include all terms of a non-transformed table, as all terms are necessarily 1 or lower *(moreover, there should automatically be at least a term per row that was significant, at P=0.05, in the files retrieved from STRING, otherwise the term would not appear in the table in the first place)*.
To set a new threshold, for instance at P=0.001, one should input `0.001`, or 3 when log-transforming in base 10. Always hit `Apply`.

Log base: choose the base for the logarithm.

DPI: choose the output image resolution in DPI *(dot per inch)*. The higher, the larger the image.

terms height: differently from the heatmap, bubble plots are always drawn such as all terms that survived the FDR cutoff and User selection are always readable. This parameter specifies how much distant (vertically) each term should be drawn in the resulting bubble plot. Defaults to `0.25`.

Apply: Applies the current settings to the table, and shows how the settings impact on the table.

Choose terms..: This button opens a dialog to choose the terms. An example:

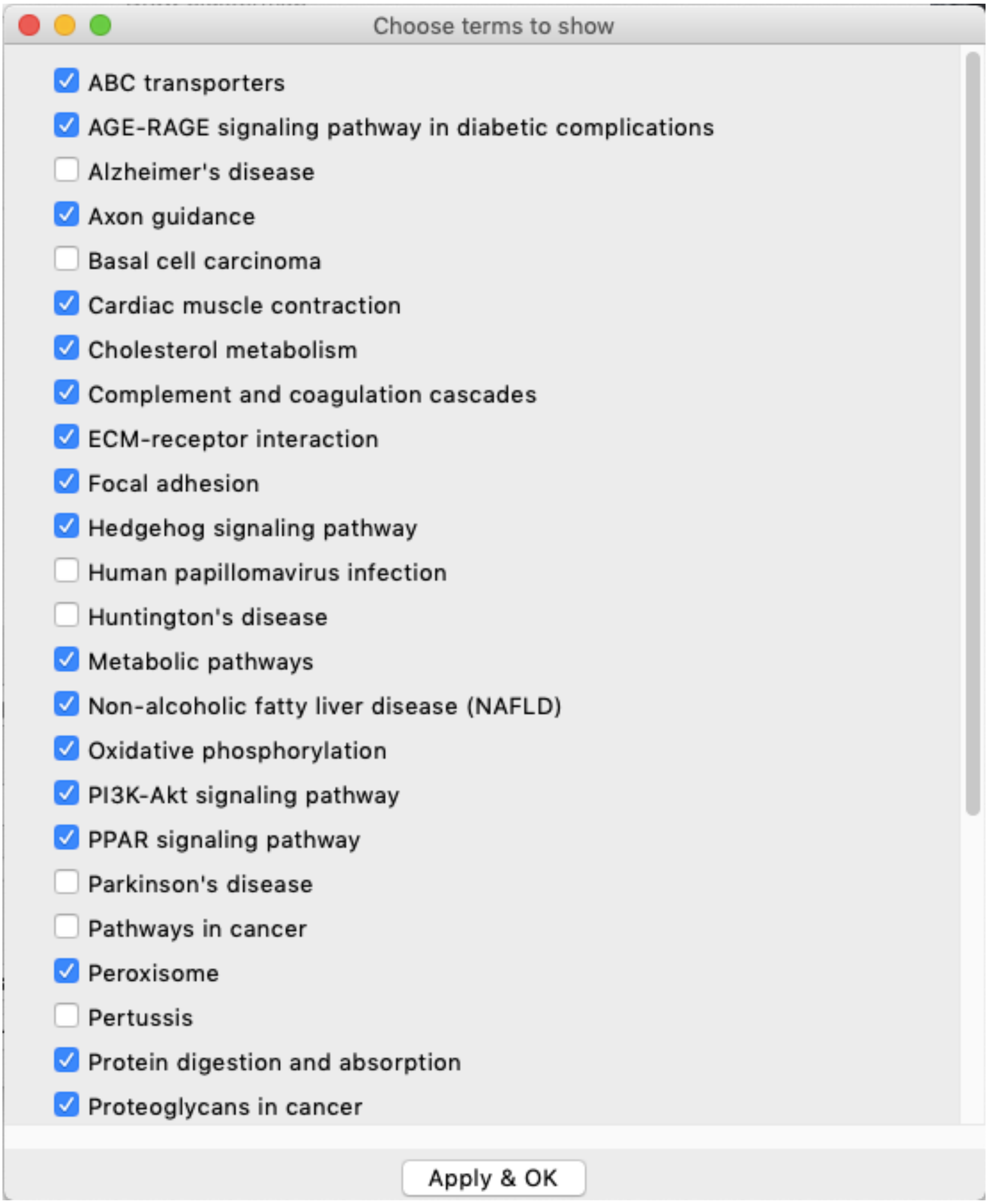

In this example, the results table contains terms that are irrelevant in the analysis being made. When loading a new table, all terms are automatically included, but the user chan choose to untick the terms that are unwanted. If a new **P-value cutoff** is applied, `restring` remembers the user choice even if some of the terms are now removed from the term list and are added back to the table at a later time.

Hit `Apply & OK` to apply the choice and close the window.

Draw bubble plot: Draws, saves and opens the bubble plot.

Reset: Reloads the input table and clears term selection.

Help: Opens a dialog that briefly outlines the procedure.

Online Manual: Opens the default browser at the bubble plot help section.

Close: Closes the window.

5 | Configuring the analysis.

Species

`restring` defaults to *Mus musculus*. To choose another species, choose `Analysis > Set species` to open the dialog:

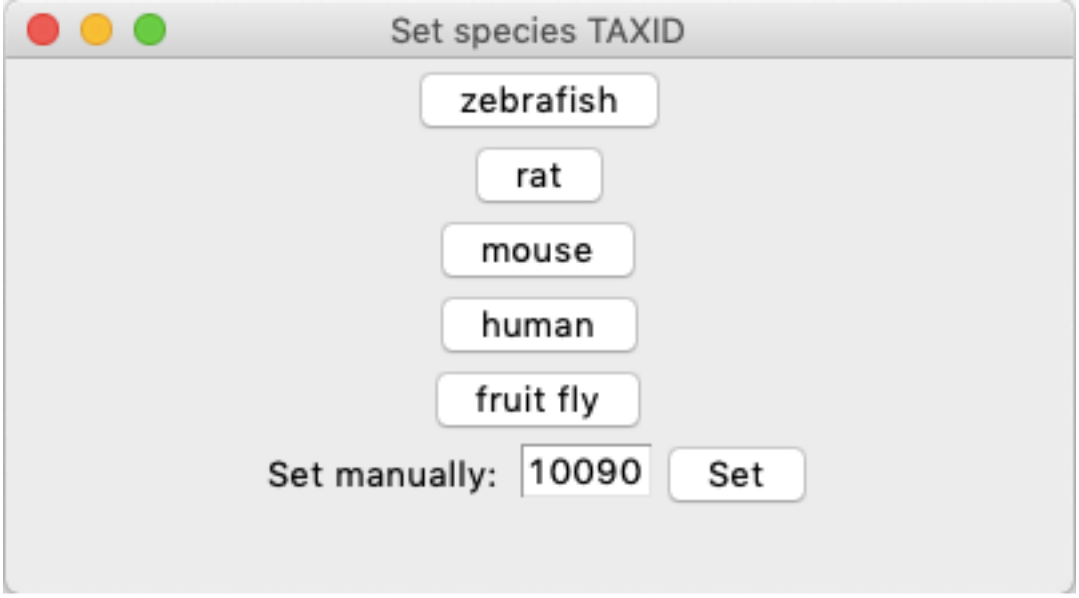

STRING accepts species in the form of taxonomy IDs. Hit the button of your species of choice or supply a custom TaxID and hit `Set`.
Head over to STRING's doc to know if your species is supported.

DE genes settings

`restring` accepts as gene lists input something like this sample data.
The input contains information of the gene name *(we developed* `restring` *having the* official gene name *in mind as the preferred gene identifier, as that's always the case among researchers in our experience)* and information about the *direction* of the change of expression with respect to experimental groups. This is the implied convention:

```
gene ID  |  cond1  | cond2  | cond1_vs_cond2 |  log2FC |
---------|---------------------------------------------|
```

```
    gene1   |     143   |  748    |       0.191      |  -2.38   |
  ----------|---------------------------------------------------|
    gene2   |     50    |   4     |       12.5       |   3.64   |
```

In this example, cond1 and cond2 are the two experimental condition where the abundance of the transcript has been estimated. Every RNAseq analysis contains at least the log2FC *(base 2, log-transformed ratio of the expression values)*, that tells if the gene is upregulated or downregulated.

`restring` follows this convention:
UP is upregulated in cond1 versus cond2. That's the case of gene2. Log2FC is > 0. **DOWN** is downregulated in cond1 versus cond2. That's the case of gene1. Log2FC is < 0.
`restring` does not actually care about the magnitude of Log2FC: that's from the pre-processing of the genes that the researcher is interested in.
Depending on how to treat the *directionality* information, there are four types of different analyses:

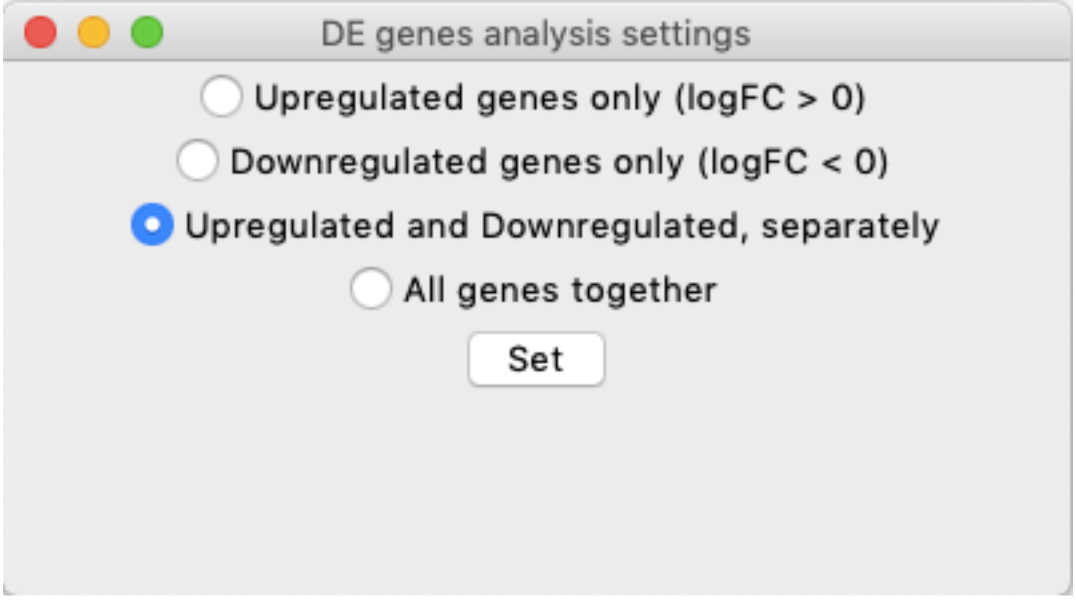

Upregulated genes only: Functional enrichment info is searched for upregulated genes only. Enrichment is performed on upregulated genes only *(Arrange the input data so as "upregulated" in your experiment matches the implied convention)*.

Downregulated genes only: Functional enrichment info is searched for downregulated genes only. Enrichment is performed on downregulated genes only *(Arrange the input data so as "downregulated" in your experiment matches the implied convention)*.

Upregulated and Downregulated, separately: This is the **default** option. For every comparison, both upregulated and downregulated genes are considered, but separately. This means that functional enrichment info is retrieved for upregulated and downregulated genes separately, but the terms are aggregated from both.

If a term shows up in both UP and DOWN gene lists, then the lowest P-value one is recorded.

All genes together: Functional enrichment info is searched for all genes together, and the resulting aggregation will reflect the functional enrichment analysis retrieved with all genes

together *(still supply a gene list that has a number, for each gene ID, in the second column. Just write any number.)*

Tip: To avoid accumulating STRING files, consider setting a different output folder any time the analysis parameters are varied. Notwithstanding, `restring` clearly labels what enrichment files come from which gene lists: `UP`, `DOWN` or `ALL` are prepended to each table retrieved from STRING.

Set background

In the words of [Szklarczyk *et al.* 2021's paper]:

"An increasing number of STRING users enter the database not with a single protein as their query, but with a set of proteins. [..] STRING will perform automated pathway-enrichment analysis on the user's input and list any pathways or functional subsystems that are observed more frequently than expected (using hypergeometric testing, against a statistical background of either the entire genome or a user-supplied background gene list)."

By default, `reString` requests functional enrichment data against the statistical background of the entire genome. This is specified in the textual output during the anaysis:
Running the analysis against a statistical background of the entire genome (default).
Otherwise, it is possible to specify a background that will be applied to all input files, via `Analysis > Custom background`. You will be prompted to open a `.csv`, `.tsv`, `.xls`, `.xlsx` file that need to be a headerless, one-column file containing your custom background entries. Alternatively, you can place one entry per line in a `.txt` file. During the analysis, this will be specified as follows in the textual output:
Running the analysis against a statistical background of user-supplied terms.

Clear Background

To clear (empty) the custom background and revert to the default background (the entire genome), choose `Analysis > Clear custom background`. A message will confirm that the background has been cleared.

Choosing a specific STRING version

In the menu, choose `Analysis > Choose STRING version` to open the following dialog:

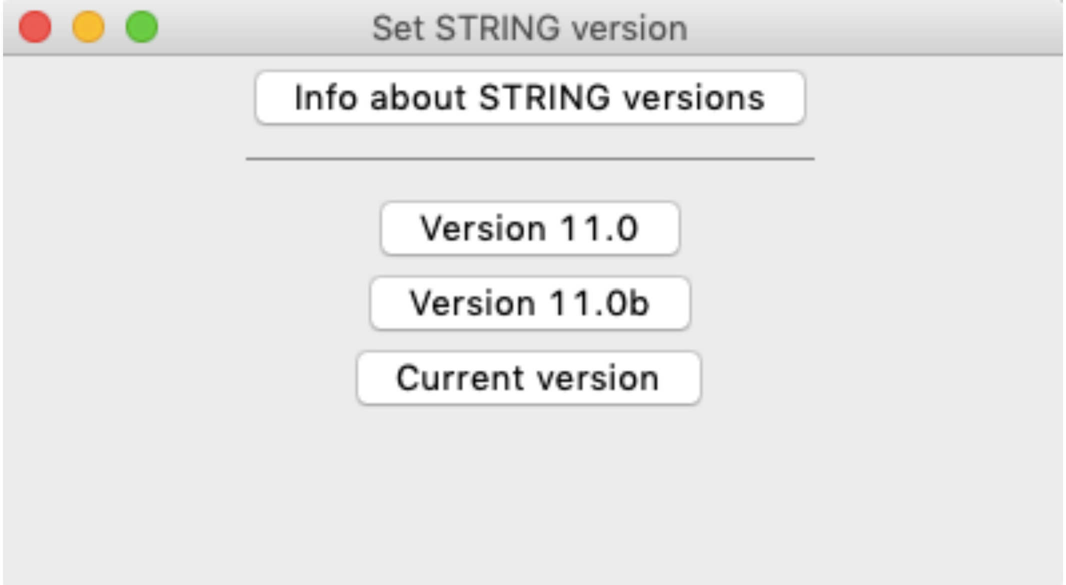

`reString` is compatible with the output produced from STRING API version 11.0 and above. To get info about past and current STRING releases, see [here]() or hit `Info about STRING versions` in the window.
`reString` always defaults to the lastest release, but for compatibility purposes other versions (11.0b or 11.0) can be selected.

Need help?

Investigating an issue

Please [let us know if you have any issue](). From installation, to usage, to unforeseen application hiccups, there is a form to request assistance. Just hit `New issue` to start a new request. Files can be drag-and-dropped into the form as well, and your request can be previewed before being finalized.
To help up pin down the issue, please always include details about your machine setup (CPU, RAM, GPU, vendor) as well as your Python, OS and `restring` version. To further help investigating the matter, you should also include a procedure to allow us to replicate the issue in order to fix it (if possible, also include your input files).
If you've been encountering an issue, chances are that some other people have already stumbled over the same problem, and the answer might already be around in the [Issues]() section.

Reporting a bug

Most of the time, `restring` communicates what it's doing by printing messages in the application window(s). In rare circumstances, errors are printed only to the **terminal window** *(the one you've launched* `restring-gui` *from, and that's still around)*:

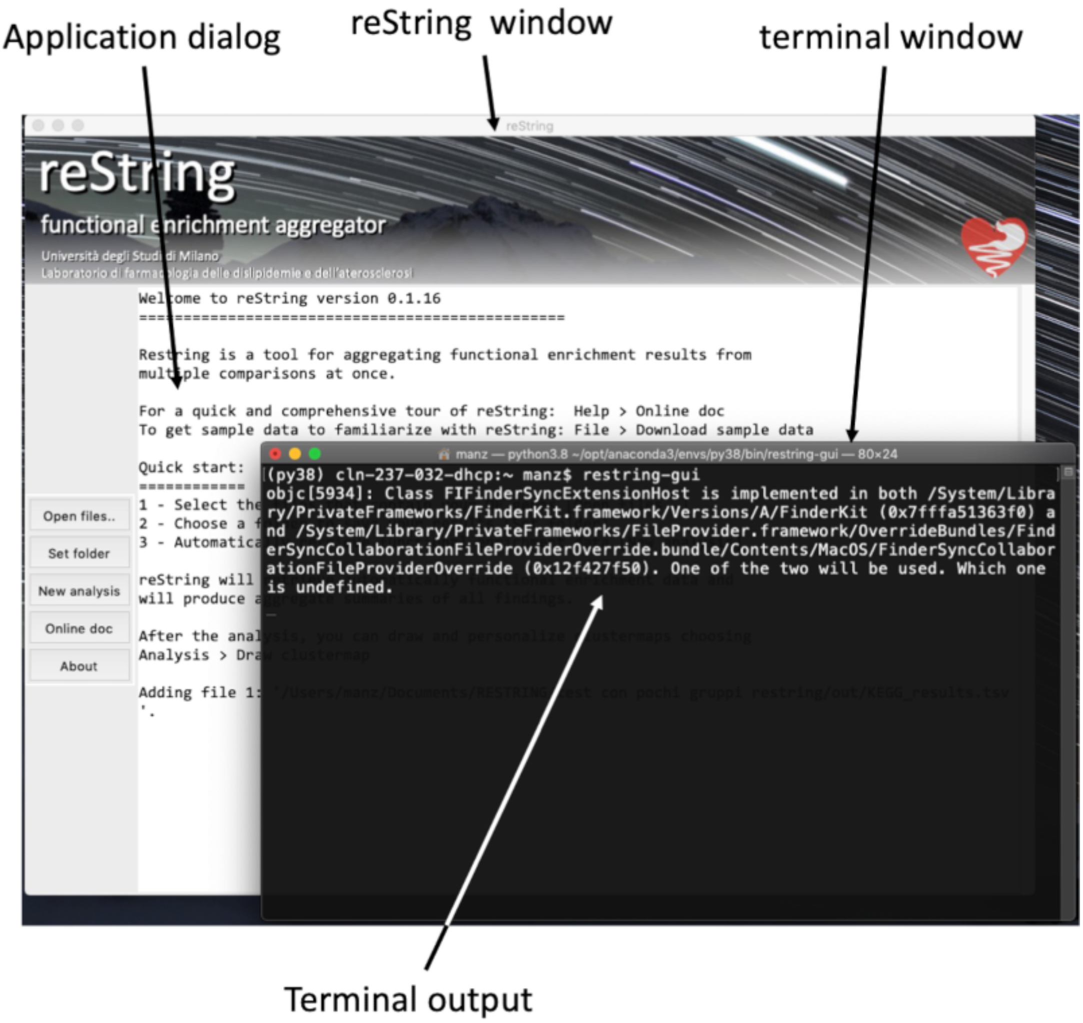

In addition to all information needed to investigate an issue (see above section), please include all **terminal output** (copy-and-paste the text, or drop a screenshot) in your bug report. Help us improve `restring` and report any bug [here](#).

Requesting a new feature

If you feel like `restring` should be including some new awesome feature, please [let us know](#)! We are aimed at making `restring` richer and more user-friendly.

Known bugs

- The repository of the ARM version of Raspberry Pi OS is sometimes having problems with keeping binaries up to date or working properly, and some of them might be required by `restring`. If you experience installation troubles with that distribution, wait for the developers to get all packages up to date.
- When drawing a heatmap or a clustermap, if you load the wrong table type (e.g. a "results"-type table instead of a "summary"-type table, `restring` will complain but it will output some weird error to the terminal. Always keep an eye on what's going on in the terminal, as explained [here](#).

`restring` as a Python module

1 | Download required files from STRING

Head over to String, and analyze your gene/protein list. Please refer to the String documentation for help. After running the analysis, hit the Σ Analysis button at the bottom of the page. This allows to download the results as tab delimited text files.

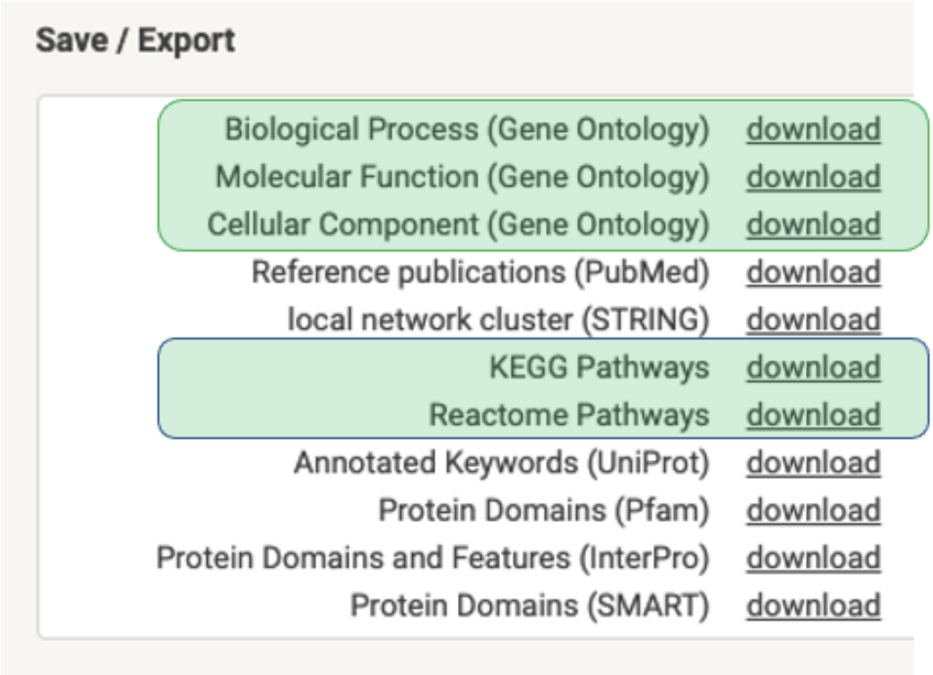

`restring` is designed to work with the results types highlighted in green. For each one of your experimental settings, create a folder with a name that will serve as a label for it. Here's how our sample data is arranged:

- ctrl_t0_red_vs_blue
- ctrl_t0_red_vs_green
- ctrl_t1_green_vs_blue
- ctrl_t1_red_vs_blue
- ctrl_t1_red_vs_green
- treatment_t0_red_vs_blue
- treatment_t0_red_vs_green
- treatment_t1_green_vs_blue
- treatment_t1_red_vs_blue
- treatment_t1_red_vs_green

Not all comparisons resulted in a DE gene list that's long enough to generate functional enrichment results (see image above), thus a few comparisons *(folders)* are missing. When the DE gene list was sufficiently long to generate results for all analyses, this is what the folder content looks like *(example of one folder)*:

- DOWN_enrichment.Component.tsv
- DOWN_enrichment.KEGG.tsv
- DOWN_enrichment.Process.tsv
- DOWN_enrichment.RCTM.tsv
- UP_enrichment.Component.tsv
- UP_enrichment.Function.tsv
- UP_enrichment.KEGG.tsv
- UP_enrichment.Process.tsv
- UP_enrichment.RCTM.tsv

For each enrichment (`KEGG`, `Component`, `Function`, `Process` and `RCTM`), we fed String with DE genes that were either up- or downregulated with respect of one of the genotypes of the

analysis. `restring` **makes use** of the `UP` and `DOWN` labels in the filenames to know what direction the analysis went *(it's possible to aggregate* `UP` *and* `DOWN` *DE genes together)*.
It's OK to have folders that don't contain all files (if there were insufficient DE genes to produce some), like in the folder `ctrl_t0_green_VS_ctrl_t0_blue_FC` that you will find in your output directory after the analysis has finished.

2 | Aggregating the results

Once everything is set up, we can run `restring` to aggregate info from all the sparse results. The following example makes use of the String results that can be found in sample data.

```
import restring

dirs = restring.get_dirs()
print(dirs)
['ctrl_t0_green_VS_ctrl_t0_blue_FC',
 'ctrl_t0_green_VS_ctrl_t0_red_FC',
 'ctrl_t0_red_VS_ctrl_t0_blue_FC',
 'ctrl_t1_green_VS_ctrl_t1_blue_FC',
 'ctrl_t1_green_VS_ctrl_t1_red_FC',
 'ctrl_t1_red_VS_ctrl_t1_blue_FC',
 'treatment_t0_green_VS_treatment_t0_blue_FC',
 'treatment_t0_green_VS_treatment_t0_red_FC',
 'treatment_t0_red_VS_treatment_t0_blue_FC',
 'treatment_t1_green_VS_treatment_t1_blue_FC',
 'treatment_t1_green_VS_treatment_t1_red_FC',
 'treatment_t1_red_VS_treatment_t1_blue_FC']
```

`get_dirs()` returns a `list` of all folders within the current directory, to the excepion of folders beginning with __ or `.`. We can start aggregating results with default parameters (`KEGG` pathways for both `UP` and `DOWN` regulated genes).

```
db = restring.aggregate_results(dirs)
Start walking the directory structure.

Parameters
----------
folders: 12
kind=KEGG
directions=['UP', 'DOWN']

Processing directory: ctrl_t0_green_VS_ctrl_t0_blue_FC
Processing directory: ctrl_t0_green_VS_ctrl_t0_red_FC
	Processing file DOWN_enrichment.KEGG.tsv
	Processing file UP_enrichment.KEGG.tsv
Processing directory: ctrl_t0_red_VS_ctrl_t0_blue_FC
	Processing file DOWN_enrichment.KEGG.tsv
	Processing file UP_enrichment.KEGG.tsv
Processing directory: ctrl_t1_green_VS_ctrl_t1_blue_FC
Processing directory: ctrl_t1_green_VS_ctrl_t1_red_FC
	Processing file DOWN_enrichment.KEGG.tsv
	Processing file UP_enrichment.KEGG.tsv
Processing directory: ctrl_t1_red_VS_ctrl_t1_blue_FC
	Processing file DOWN_enrichment.KEGG.tsv
	Processing file UP_enrichment.KEGG.tsv
Processing directory: treatment_t0_green_VS_treatment_t0_blue_FC
Processing directory: treatment_t0_green_VS_treatment_t0_red_FC
	Processing file UP_enrichment.KEGG.tsv
Processing directory: treatment_t0_red_VS_treatment_t0_blue_FC
```

```
        Processing file DOWN_enrichment.KEGG.tsv
        Processing file UP_enrichment.KEGG.tsv
Processing directory: treatment_t1_green_VS_treatment_t1_blue_FC
        Processing file DOWN_enrichment.KEGG.tsv
        Processing file UP_enrichment.KEGG.tsv
Processing directory: treatment_t1_green_VS_treatment_t1_red_FC
        Processing file DOWN_enrichment.KEGG.tsv
        Processing file UP_enrichment.KEGG.tsv
Processing directory: treatment_t1_red_VS_treatment_t1_blue_FC
        Processing file DOWN_enrichment.KEGG.tsv
        Processing file UP_enrichment.KEGG.tsv

Processed 12 directories and 17 files.
Found a total of 165 KEGG elements.
```

Tip: you must start working in the same directory where you start `restring`, as it memorizes the starting directory at startup and would otherwise complain that it can longer locate the folders. tl;dr: don't play around with `os.chdir()`, get to the folder containing the output folders from the start. Running `aggregate_results()` with other parameters is possible:

```
help(restring.aggregate_results)
# truncated output
    Walks the given <directories> list, and reads the String .tsv files of
    defined <kind>.

    Params:
    =======
    directories: <list> of directories where to look for String files

    kind:    <str> Defines the String filetype to process. Kinds defined
in
             settings.file_types

    directions: <list> contaning the up- or down-regulated genes in a
comparison.
             Info is retrieved from either UP and/or DOWN lists.
             * Prerequisite *: generating files form String with UP and/or
DOWN regulated
             genes separately.

    verbose: <bool>; turns verbose mode on or off
```

The `kind` parameter is picked from the 5 supported String result tables:

```
print(restring.settings.file_types)
('Component', 'Function', 'KEGG', 'Process', 'RCTM')
```

To manipulate the aggregated results, it's convenient to put them into a table:

```
df = restring.tableize_aggregated(db)
```

This functions wraps the results into a handy `pandas.DataFrame` object, that can be saved as a table for further inspection:

```
df.to_csv("results.csv")
```

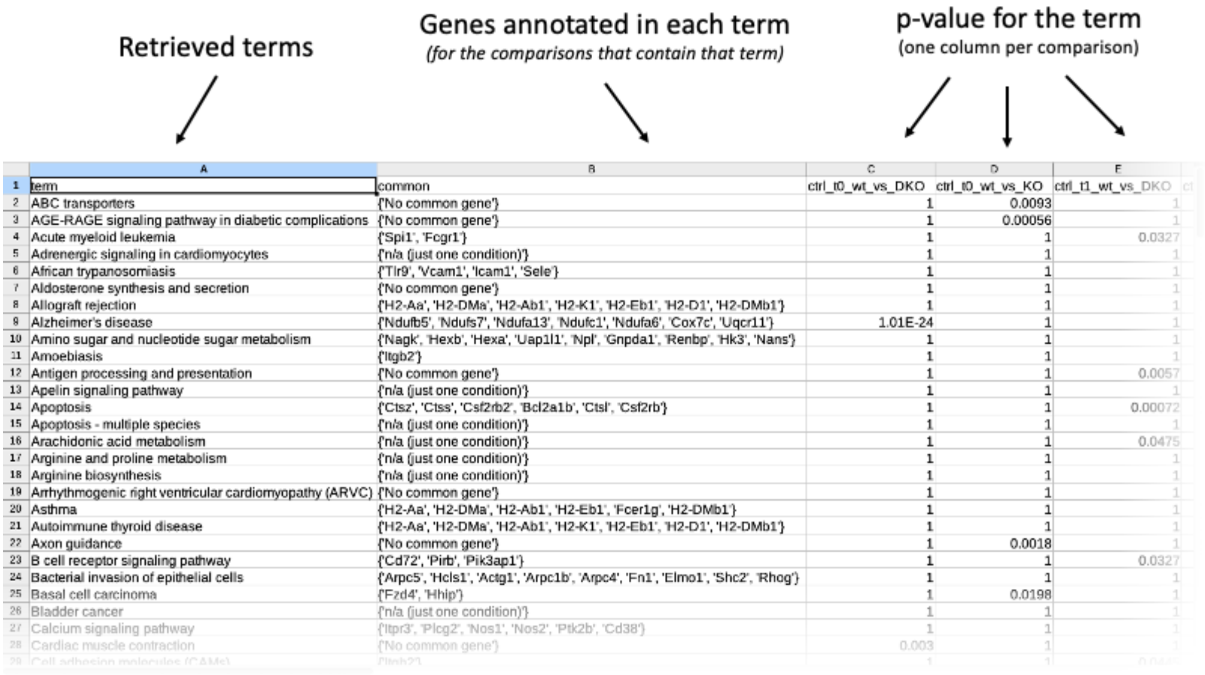

	A	B	C	D	E
1	term	common	ctrl_t0_wt_vs_DKO	ctrl_t0_wt_vs_KO	ctrl_t1_wt_vs_DKO
2	ABC transporters	{'No common gene'}	1	0.0093	1
3	AGE-RAGE signaling pathway in diabetic complications	{'No common gene'}	1	0.00056	1
4	Acute myeloid leukemia	{'Spi1', 'Fcgr1'}	1	1	0.0327
5	Adrenergic signaling in cardiomyocytes	{'n/a (just one condition)'}	1	1	1
6	African trypanosomiasis	{'Tlr9', 'Vcam1', 'Icam1', 'Sele'}	1	1	1
7	Aldosterone synthesis and secretion	{'No common gene'}	1	1	1
8	Allograft rejection	{'H2-Aa', 'H2-DMa', 'H2-Ab1', 'H2-K1', 'H2-Eb1', 'H2-D1', 'H2-DMb1'}	1	1	1
9	Alzheimer's disease	{'Ndufb5', 'Ndufs7', 'Ndufa13', 'Ndufc1', 'Ndufa6', 'Cox7c', 'Uqcr11'}	1.01E-24	1	1
10	Amino sugar and nucleotide sugar metabolism	{'Nagk', 'Hexb', 'Hexa', 'Uap1l1', 'Npl', 'Gnpda1', 'Renbp', 'Hk3', 'Nans'}	1	1	1
11	Amoebiasis	{'Itgb2'}	1	1	1
12	Antigen processing and presentation	{'No common gene'}	1	1	0.0057
13	Apelin signaling pathway	{'n/a (just one condition)'}	1	1	1
14	Apoptosis	{'Ctsz', 'Ctss', 'Csf2rb2', 'Bcl2a1b', 'Ctsl', 'Csf2rb'}	1	1	0.00072
15	Apoptosis - multiple species	{'n/a (just one condition)'}	1	1	1
16	Arachidonic acid metabolism	{'n/a (just one condition)'}	1	1	0.0475
17	Arginine and proline metabolism	{'n/a (just one condition)'}	1	1	1
18	Arginine biosynthesis	{'n/a (just one condition)'}	1	1	1
19	Arrhythmogenic right ventricular cardiomyopathy (ARVC)	{'No common gene'}	1	1	1
20	Asthma	{'H2-Aa', 'H2-DMa', 'H2-Ab1', 'H2-Eb1', 'Fcer1g', 'H2-DMb1'}	1	1	1
21	Autoimmune thyroid disease	{'H2-Aa', 'H2-DMa', 'H2-Ab1', 'H2-K1', 'H2-Eb1', 'H2-D1', 'H2-DMb1'}	1	1	1
22	Axon guidance	{'No common gene'}	1	0.0018	1
23	B cell receptor signaling pathway	{'Cd72', 'Pirb', 'Pik3ap1'}	1	1	0.0327
24	Bacterial invasion of epithelial cells	{'Arpc5', 'Hcls1', 'Actg1', 'Arpc1b', 'Arpc4', 'Fn1', 'Elmo1', 'Shc2', 'Rhog'}	1	1	1
25	Basal cell carcinoma	{'Fzd4', 'Hhip'}	1	0.0198	1
26	Bladder cancer	{'n/a (just one condition)'}	1	1	1
27	Calcium signaling pathway	{'Itpr3', 'Plcg2', 'Nos1', 'Nos2', 'Ptk2b', 'Cd38'}	1	1	1
28	Cardiac muscle contraction	{'No common gene'}	0.003	1	1
29	Cell adhesion molecules (CAMs)	[illegible]	1	1	[illegible]

The table contains all terms cumulatively retrieved from all comparisons *(directories/columns)*. For every term, common genes (if any) are listed. These common genes only include comparisons where the term actually shows up. If the term just appears in exactly one comparison, this is explicitly stated: `n/a (just one condition)`. P-values are the ones retrieved from the String tables *(the lower, the better)*. Missing p-values are represented with `1` (this can be set to anything `str()` accepts when calling `tableize_aggregated()` with the `not_found` parameter).

These 'aggregated' tables are useful for charting the results (see later). There's other info that can be extracted form aggregated results, in the form of a 'summary' table:

```
res = restring.summary(db)
res.to_csv("summary.csv")
```

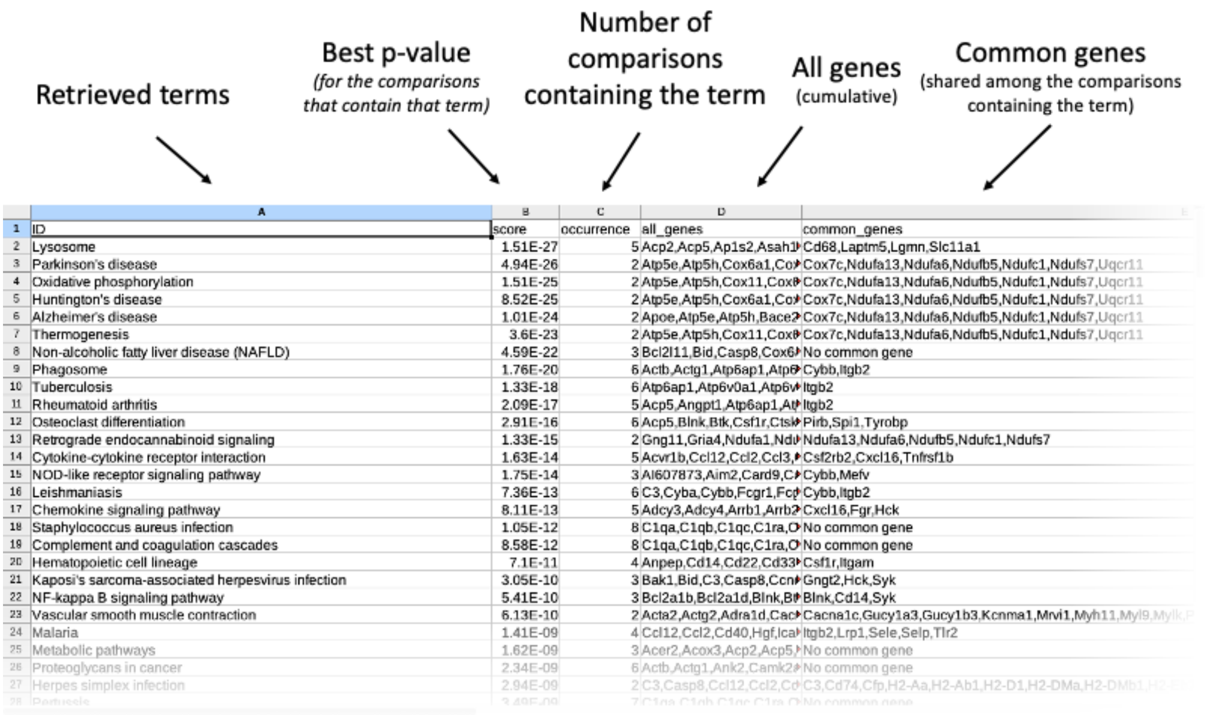

	A	B	C	D	E
1	ID	score	occurrence	all_genes	common_genes
2	Lysosome	1.51E-27	5	Acp2,Acp5,Ap1s2,Asah1	Cd68,Laptm5,Lgmn,Slc11a1
3	Parkinson's disease	4.94E-26	2	Atp5e,Atp5h,Cox6a1,Co	Cox7c,Ndufa13,Ndufa6,Ndufb5,Ndufc1,Ndufs7,Uqcr11
4	Oxidative phosphorylation	1.51E-25	2	Atp5e,Atp5h,Cox11,Cox	Cox7c,Ndufa13,Ndufa6,Ndufb5,Ndufc1,Ndufs7,Uqcr11
5	Huntington's disease	8.52E-25	2	Atp5e,Atp5h,Cox6a1,Co	Cox7c,Ndufa13,Ndufa6,Ndufb5,Ndufc1,Ndufs7,Uqcr11
6	Alzheimer's disease	1.01E-24	2	Apoe,Atp5e,Atp5h,Bace2	Cox7c,Ndufa13,Ndufa6,Ndufb5,Ndufc1,Ndufs7,Uqcr11
7	Thermogenesis	3.6E-23	2	Atp5e,Atp5h,Cox11,Cox	Cox7c,Ndufa13,Ndufa6,Ndufb5,Ndufc1,Ndufs7,Uqcr11
8	Non-alcoholic fatty liver disease (NAFLD)	4.59E-22	3	Bcl2l11,Bid,Casp8,Cox6	No common gene
9	Phagosome	1.76E-20	6	Actb,Actg1,Atp6ap1,Atp6	Cybb,Itgb2
10	Tuberculosis	1.33E-18	6	Atp6ap1,Atp6v0a1,Atp6v	Itgb2
11	Rheumatoid arthritis	2.09E-17	5	Acp5,Angpt1,Atp6ap1,At	Itgb2
12	Osteoclast differentiation	2.91E-16	6	Acp5,Blnk,Btk,Csf1r,Ctsk	Pirb,Spi1,Tyrobp
13	Retrograde endocannabinoid signaling	1.33E-15	2	Gng11,Gria4,Ndufa1,Ndu	Ndufa13,Ndufa6,Ndufb5,Ndufc1,Ndufs7
14	Cytokine-cytokine receptor interaction	1.63E-14	5	Acvr1b,Ccl12,Ccl2,Ccl3,	Csf2rb2,Cxcl16,Tnfrsf1b
15	NOD-like receptor signaling pathway	1.75E-14	3	AI607873,Aim2,Card9,C	Cybb,Mefv
16	Leishmaniasis	7.36E-13	6	C3,Cyba,Cybb,Fcgr1,Fc	Cybb,Itgb2
17	Chemokine signaling pathway	8.11E-13	5	Adcy3,Adcy4,Arrb1,Arrb2	Cxcl16,Fgr,Hck
18	Staphylococcus aureus infection	1.05E-12	8	C1qa,C1qb,C1qc,C1ra,C	No common gene
19	Complement and coagulation cascades	8.58E-12	8	C1qa,C1qb,C1qc,C1ra,C	No common gene
20	Hematopoietic cell lineage	7.1E-11	4	Anpep,Cd14,Cd22,Cd33	Csf1r,Itgam
21	Kaposi's sarcoma-associated herpesvirus infection	3.05E-10	3	Bak1,Bid,C3,Casp8,Ccn	Gngt2,Hck,Syk
22	NF-kappa B signaling pathway	5.41E-10	3	Bcl2a1b,Bcl2a1d,Blnk,Bt	Blnk,Cd14,Syk
23	Vascular smooth muscle contraction	6.13E-10	2	Acta2,Actg2,Adra1d,Cac	Cacna1c,Gucy1a3,Gucy1b3,Kcnma1,Mrvi1,Myh11,Myl9,Mylk,P
24	Malaria	1.41E-09	4	Ccl12,Ccl2,Cd40,Hgf,Ica	Itgb2,Lrp1,Sele,Selp,Tlr2
25	Metabolic pathways	1.62E-09	3	Acer2,Acox3,Acp2,Acp5,	No common gene
26	Proteoglycans in cancer	2.34E-09	6	Actb,Actg1,Ank2,Camk2	No common gene
27	Herpes simplex infection	2.94E-09	2	C3,Casp8,Ccl12,Ccl2,Cd	C3,Cd74,Cfp,H2-Aa,H2-Ab1,H2-D1,H2-DMa,H2-DMb1,H2-[illegible]
28	Pertussis	3.49E-09	7	C1qa,C1qb,C1qc,C1ra,C	No common gene

These can be useful to find the most interesting terms across all comparisons: better p-value, presence in most/selected comparisons), as well as finding the most recurring DE genes for each term.

3 | Visualizing the results with a clustermap

'aggregated'-type tables can be readily transformed into beautiful clustermaps. This is simply done by passing either the `df` object previolsly created with `tableize_aggregated()`, or the file name of the table that was saved from that object to the `draw_clustermap()` function:

```
clus = restring.draw_clustermap("results.csv")
```

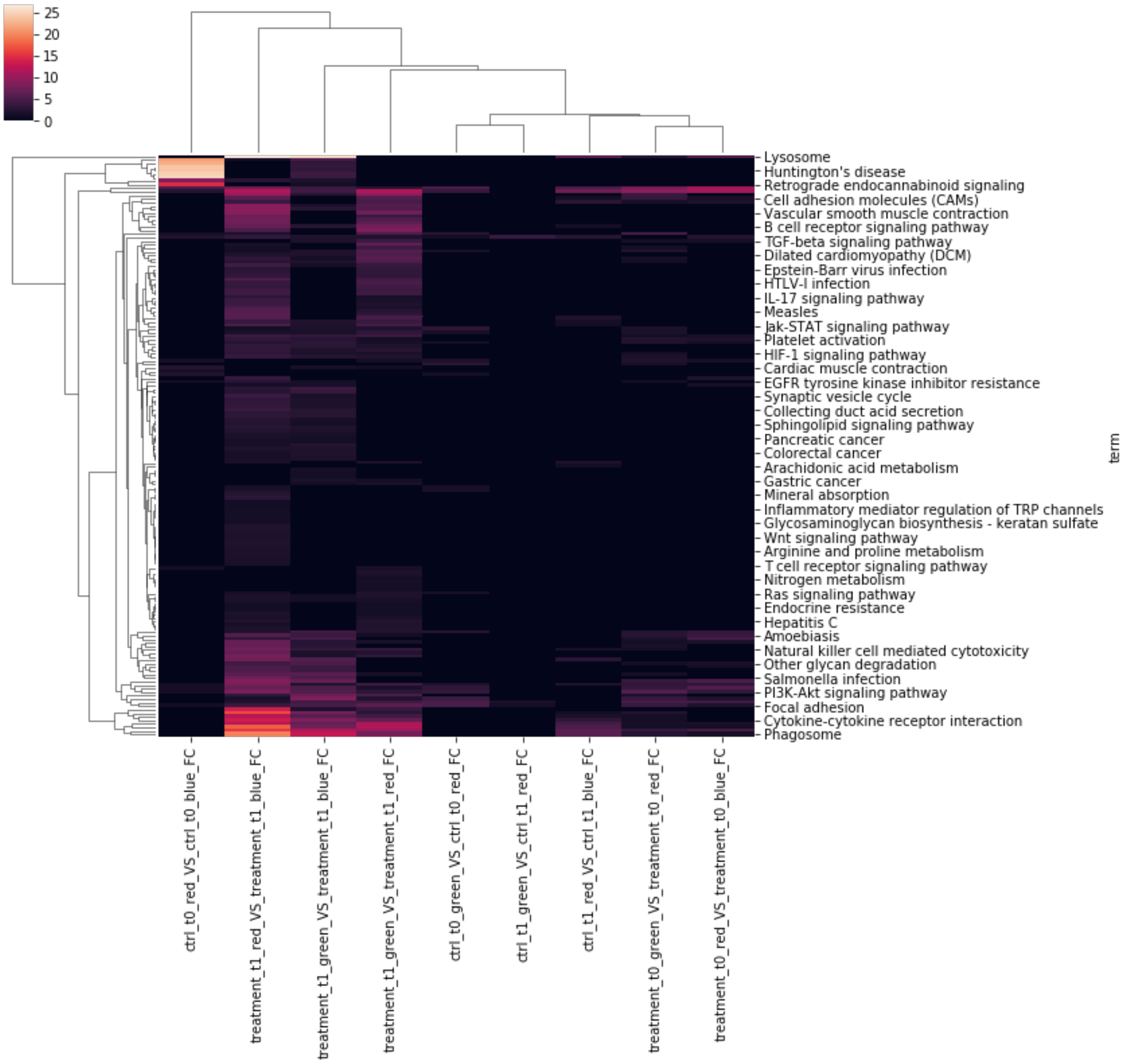

(The output may vary depending on your version of plotting libraries) Note that `draw_clustermap()` actually *returns* the `seaborn.matrix.ClusterGrid` object that it generates internally, this might come handy to retrieve the reordered (clustered) elements (more on that later).

The drawing function is basically a wrapper for `seaborn.clustermap()`, of which retains all the flexible customization options, but allows for an immediate tweaking of the picture. For instance, we might just want to plot the highest-ranking terms and have all of them clearly written on a readable heatmap:

```
clus = restring.draw_clustermap("results.csv", pval_min=6, readable=True)
```

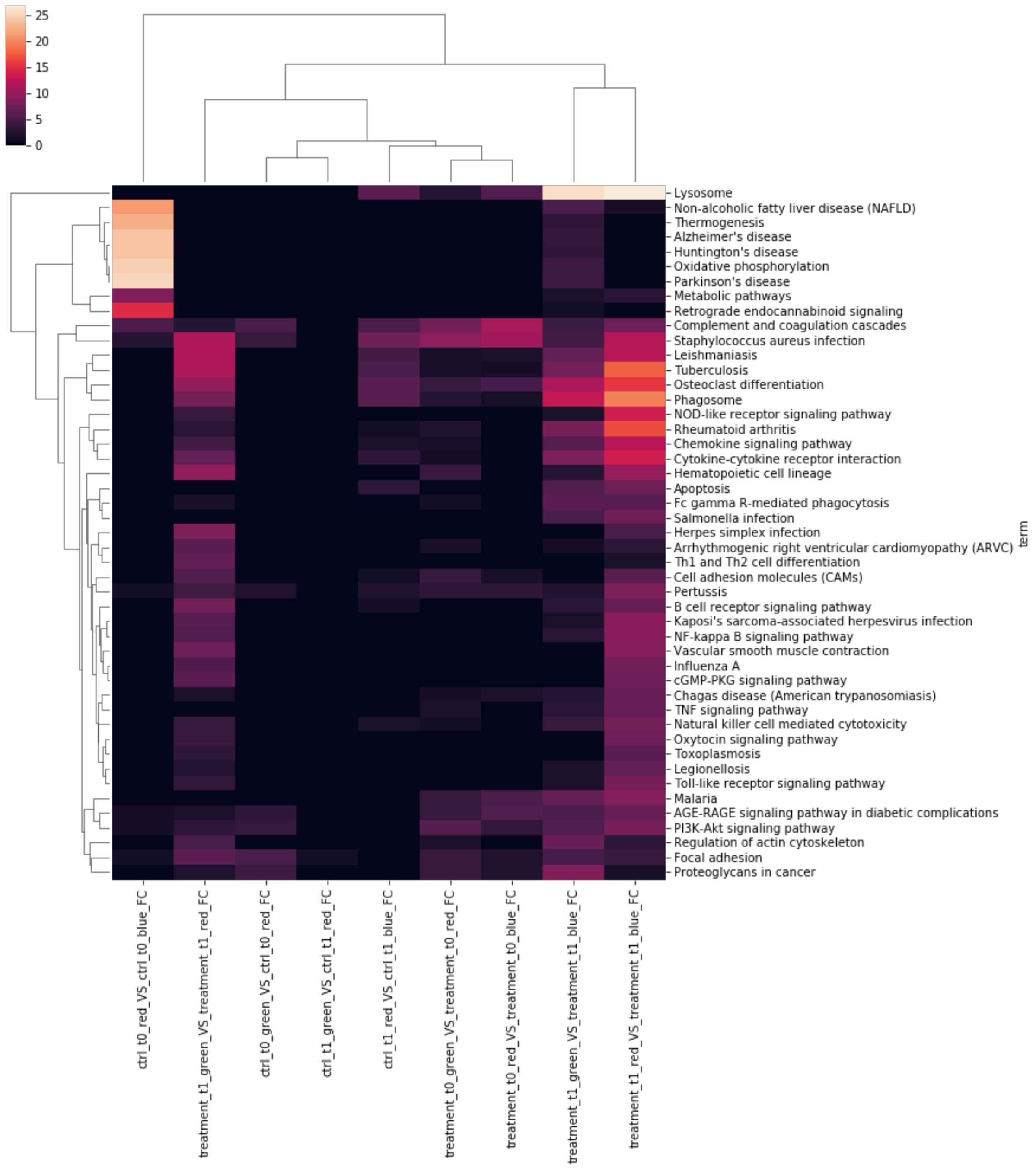

4 | Polishing up

More tweaking is possibile:

```
help(restring.draw_clustermap)
Help on function draw_clustermap in module restring.restring:

draw_clustermap(data, figsize=None, sort_values=None, log_transform=True,
log_base=10,
                   log_na=0, pval_min=None, custom_index=None,
custom_cols=None,
                  unwanted_terms=None, title=None, title_size=24,
savefig=False,
```

```
               outfile_name='aggregated results.png', dpi=300,
readable=False,
               return_table=False, **kwargs)

    Draws a clustermap of an 'aggregated'-type table.

    This functions expects this table layout (example):

    terms │ exp. cond 1 │ exp. cond 2 │ exp cond n ..
    ──────┼─────────────┼─────────────┼────────────..
    term 1│    0.01     │      1      │  0.00023
    ──────┼─────────────┼─────────────┼────────────..
    term 2│      1      │    0.05     │      1
    ──────┼─────────────┼─────────────┼────────────..
      ..  │     ..      │     ..      │     ..

    *terms* must be indices of the table. If present, 'common' column
    will be ignored.

    Params
    ------

    data      A <pandas.DataFrame object>, or a filename. If a filename is
              given, this will try to load the table as if it were
produced
              by tableize_aggregated() and saved with
pandas.DataFrame.to_csv()
              as a .csv file, with ',' as separator.

    sort_values   A <str> or <list> of <str>. Sorts the table accordingly.
Please
              *also* set col_cluster=False (see below,
seaborn.clustermap()
              additional parameters), otherwise columns will still be
clustered.

    log_transform   If True, values will be log-transformed. Defaults to
True.
              note: values are turned into -log values, thus p-value of
0.05
              gets transformed into 1.3 (with default parameters), as
              10^-1.3 ~ 0.05

    log_base  If log_transform, this base will be used as the logarithm
base.
              Defaults to 10

    log_na    When unable to compute the logarithm, this value will be
used
              instead. Defaults to 0 (10^0 == 1)

    pval_min  Trims values to the ones matching *at least* this p value.
If
              log_transform with default values, this needs to be set
accordingly
              For example, if at least p=0.01 is desired, then aim for
              pval_min=2 (10^-2 == 0.01)
```

```
    custom_cols   It is possible to pass a <list> of <str> to draw only
specified
              columns of input table

    custom_index  It is possible to pass a <list> of <str> to draw only
specified
              rows of input table

    unwanted_terms   If a <list> of <str> is supplied,

    readable  If set to False (default), the generated heatmap will be of
reasonable
              size, possibly not showing all term descriptors. Setting
readable=True
              will result into a heatmap where all descriptors are visible
(this might
              result into a very tall heatmap)

    savefig   If True, a picture of the heatmap will be saved in the
current directory.

    outfile_name  The file name of the picture saved.

    dpi       dpi resolution of saved picture. Defaults to 300.

    return_table   If set to True, it also returns the table that was
manipulated
              internally to draw the heatmap (with all modifications
applied).
              Returns: <seaborn.matrix.ClusterGrid>,
<pandas.core.frame.DataFrame>

    **kwargs    The drawing is performed by seaborn.clustermap(). All
additional
              keyword arguments are passed directly to it, so that the
final picture
              can be precisely tuned. More at:
              https://seaborn.pydata.org/generated/seaborn.clustermap.html
```

With a few brush strokes we can obtain we picture we're looking for. Example:

```
bad = [
    "Alzheimer's disease",
    "Huntington's disease",
    "Parkinson's disease",
    "Retrograde endocannabinoid signaling",
    "Staphylococcus aureus infection",
    "Tuberculosis",
    "Leishmaniasis",
    "Herpes simplex infection",
    "Kaposi's sarcoma-associated herpesvirus infection",
    "Proteoglycans in cancer",
    "Pertussis",
    "Malaria",
    "I'm not in the index"
]

clus = restring.draw_clustermap(
    "results.csv", pval_min=8, title="Freakin' good results",
unwanted_terms=bad,
```

```
    sort_values="treatment_t1_wt_vs_DKO", row_cluster=False, annot=True,
)
```

Freakin' good results

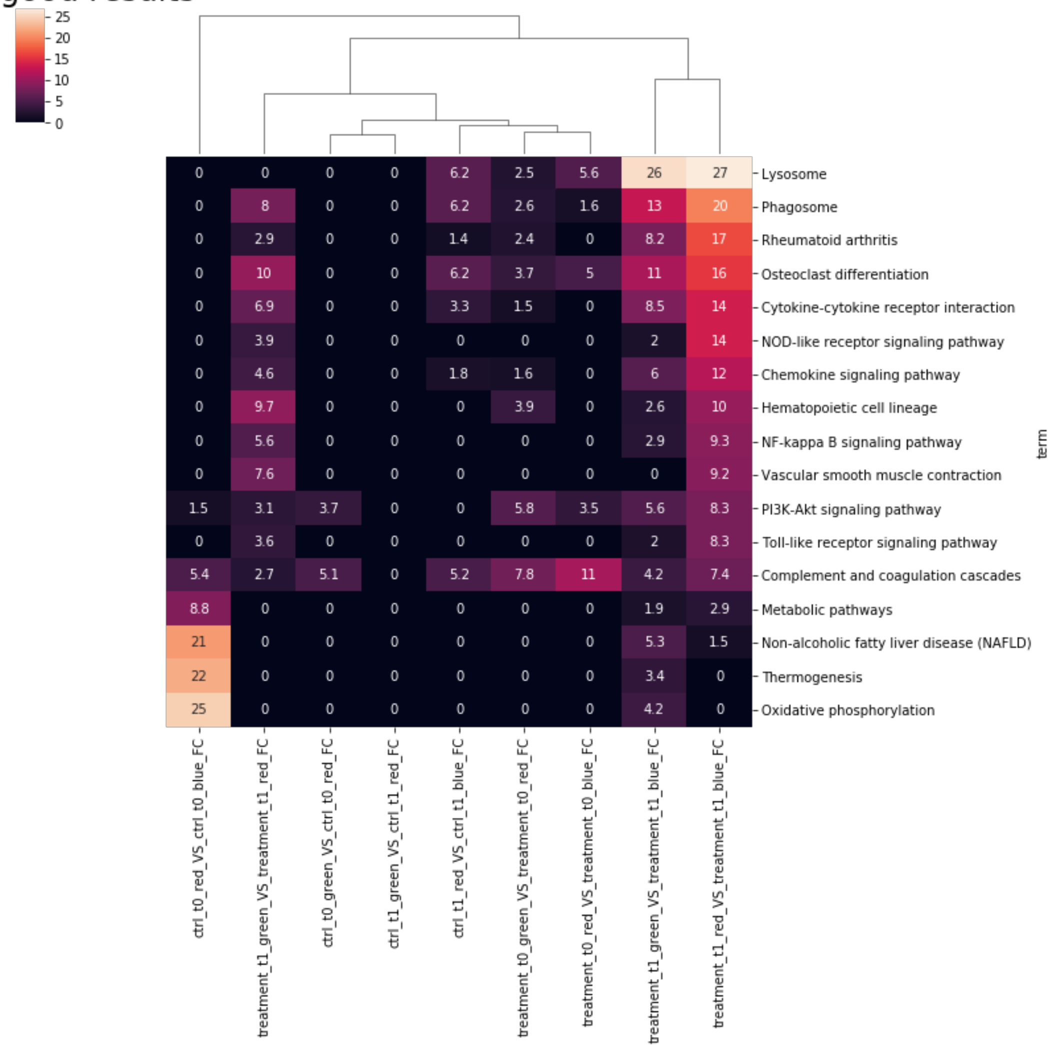

Supplement: Supplementary file 2 — Supplementary Information 2. [file 41598_2021_2528_MOESM2_ESM.pdf]
